# Protocol for a feasibility study and process evaluation of a psychosocially modelled diabetes education programme for young people with type 1 diabetes: the Youth Empowerment Skills (YES) programme

**DOI:** 10.1136/bmjopen-2022-062971

**Published:** 2022-06-08

**Authors:** Dulmini Kariyawasam, Tayana Soukup, Judith Parsons, Nick Sevdalis, Maria Baldellou Lopez, Rita Forde, Khalida Ismail, Marie Jones, Martha Ford-Adams, Nardos Yemane, Siobhan Pender, Stephen Thomas, Trevor Murrells, Alex Silverstien, Angus Forbes

**Affiliations:** 1 Department of Diabetes and Endocrinology, Guy’s and St.Thomas ‘NHS foundation Trust, London, UK; 2 Health Service and Population Research Department, King's College London, London, UK; 3 Division of Care Long-term Conditions, King's College London Florence Nightingale Faculty of Nursing, Midwifery and Palliative Care, London, UK; 4 Paediatric Diabetes Department, King's College Hospital NHS Foundation Trust, London, UK; 5 National Nursing Research Unit, King's College London, London, UK

**Keywords:** diabetes & endocrinology, general diabetes, general endocrinology

## Abstract

**Introduction:**

Adolescence is a challenging period for young people with type 1 diabetes, associated with worsening glycaemia and care disengagement. Educational interventions in this period tend to focus on diabetes-specific skills, with less emphasis on the psychosocial challenges associated with diabetes experienced by young people. To address this limitation, we codesigned with young people a psychosocially modelled programme of diabetes education, named ‘Youth Empowerment Skills’ (YES). The programme aims to facilitate a positive adaptation to life with diabetes and engagement with diabetes care through peer-based learning, immersive simulations and support from an outreach youth worker. Here, we present a protocol for a feasibility study of the YES programme.

**Methods and analysis:**

The study was designed following the Medical Research Council Complex Intervention Evaluation Framework to: test the feasibility (acceptance, implementability, recruitment and completion) of the YES programme; and estimate its efficacy in relation to metabolic and psychosocial outcomes. The study will take place in diabetes centres serving socioculturally diverse populations. We will conduct a feasibility randomised controlled trial (waiting-list design) with integrated process evaluation. Fifty young people with type 1 diabetes (aged 14–19 years) will be randomly allocated to either the YES intervention or a waiting-list control. Randomisation acceptability will be assessed with provision for a preference allocation. Outcomes will be evaluated at 6 months, at which point the waiting list participants will be exposed to the YES programme with further follow-up to 12 months. A simultaneous process evaluation will use a mixed-methods approach collecting qualitative and quantitative data. Study findings will be used to optimise the intervention components, outcome measures and recruitment methods to inform a subsequent definitive trial.

**Ethics and dissemination:**

The protocol has ethical approval from the UK Health Research Authority (approval IRAS project ID: 279877). Findings will be disseminated in multiple formats for lay and professional audiences.

**Protocol date and version:**

7 April 2021, V.1.1.

**Trial registration number:**

NCT04670198.

**Strengths and limitations of this study**

- The study will test a novel codesigned psychoeducational intervention for young people with type 1 diabetes designed to help promote a positive emotional and social adaptation to a life with diabetes.
- An integrated process evaluation within the study will develop our understanding of how to enhance study procedures and gain insights into programme implementation for future studies.
- The randomised waiting-list design allows for the comparison of the intervention and usual care exposures without the ethical implications of not providing the intervention to the control group.
- The target population for this study can be resistant to healthcare interventions and have significant school and social commitments; hence, the study will consider recruitment feasibility and the acceptability of randomisation versus preference for group allocation.

## Introduction

### Impact of type 1 diabetes on young people

Type 1 diabetes (T1DM) in adolescence and early adulthood can be a very challenging period in the lives of young people. During this period, young people can begin to dissociate from their diabetes, leading to a decline in their glycaemic control and an increased risk of acute events such as recurrent diabetic-keto-acidosis (DKA).^1–5^ Data from the most recent National Paediatric Diabetes audit in the UK showed that only 10% of young people aged 14–19 years achieved a glycated haemoglobin (HbA1c) within the current target range of ≤48 mmol/mol (6.5%); and a fifth had an HbA1c>75 mmol/mol (9%) which is associated with a high risk of diabetes complications.^3^

Furthermore, adolescence and early adulthood are also a period when young people are forming their personal and social identities, and diabetes can have a detrimental impact on their psychosocial development.^6 7^ During this period, young people can develop negative emotional constructs about diabetes, as they see their diabetes as an impediment to social relationships and life opportunities.^8^ Consequently, some young people may develop maladaptive coping strategies such as avoidance (omitting insulin and reducing their glucose monitoring), or psychological morbidities (fear, anxiety, depression and disordered eating behaviours).^9–11^ Young people with diabetes can also feel stigmatised,^12^ further driving negative coping strategies (eg, not using insulin in public or running glucose levels high to avoid hypoglycaemia).

During this period, young people are also developing their personal autonomy, which can lead to conflicts with parents who may find allowing the young person to take control over their diabetes self-management behaviours and health management challenging.^13^ Relationships can become emotionally charged with conflict, excess vigilance and distress, difficulties that can exacerbate disengagement and are associated with poorer glycaemic control.^14 15^ Negative interactions with diabetes health professionals have also been identified as contributing to young people disengaging from their care.^16^

### Current care models for young people with T1DM

Developing effective diabetes care for young people with T1DM is challenging. The transition from child to adult care has long been identified as a period when young people can disengage from their care, despite much innovation.^17–22^ In part, this is attributable to the failure of current care provision to adequately integrate the needs or life contexts of young people within that care. Young people with T1DM need support to help them develop the psychosocial skills necessary to transition positively into an adult life with diabetes, and to enable them to engage productively with healthcare providers. However, the main focus in diabetes guidelines is on intensifying glucose levels with diabetes technologies and structured education.^23 24^ Therefore, most current education programmes for young people aim to enhance self-management skills such as carbohydrate counting and insulin-dose adjustment, to help optimise glucose levels; rather than addressing how to integrate living with diabetes in relation to the wider interpersonal and social challenges that they experience in this period. Hence, while these programmes are generally effective in the adult diabetes population, young people are less likely to participate in these programmes. A recent study of 227 young people identified that the main reasons for their non-participation were a lack of time and a preference for managing their diabetes on their own.^25^ This indicates the need to reconsider both the type of educational support provided and how it is delivered to young people. While some programmes have been adapted for younger people, there is still an emphasis on technical aspects of diabetes,^26^ rather than psychosocial adjustment.

Therefore, new educational programmes are required for young people, to ensure that what they learn about managing their diabetes is contextualised with learning to live with diabetes in relation to their social and emotional development. It is also important to explore innovative methods of delivering education to young people with T1DM to make programmes more relevant, attractive and accessible to them.

### The Youth Empowerment Skills programme for young people with T1DM

The Youth Empowerment Skills (YES) programme is a novel psychoeducational intervention, which was developed through a codesign process with young people with the fundamental goal to overcome some of the challenges reviewed above. The codesign process sort to build the programme so that it addressed many of the previously identified challenges experienced by young people with diabetes. The programme uses three well-established psychological theories: social learning theory,^27^ self-regulatory theory^28^ (personal identity) and dual-process theory.^29^ These theories have been integrated into a learning model addressing: cognitive factors (knowledge, experiences, thinking processes and attitudes); environmental factors (exploring social norms and external factors that mediate behaviour); and behavioural factors (developing skills and self-efficiency, with practice, observation and rehearsal). This underpinning theory is delivered using different learning techniques: experiential and group-based learning; immersive simulations with scenarios such as treating an episode of severe hypoglycaemia or ketosis; and learning together in adventurous activities. The programme curriculum comprises the following sessions:

- Diabetes as a part of daily life.
- The psychological impact of diabetes.
- Staying safe while being away from home (parties/alcohol/drugs and diabetes).
- Immersive simulations (hypoglycaemia, DKA, impaired physical function).
- Reverse role-playing of health consultations (how to get more out of a consultation).
- Relationships (peers/partners/family).
- Attitudes towards food, weight and eating out.
- Getting to know your body (foot-care/eye screening/sexual health).
- Exercise and diabetes (activity-based sessions, eg, rock climbing).
- How to prevent diabetes emergencies.

The programme is delivered by a peer educator with T1DM, health professionals and youth workers. The youth workers also do outreach work to identify potential participants and encourage them to attend. The programme is fully manualised and Quality Institute for Self-Management Education accredited.

As part of the programme’s development, we undertook a systematic review to identify studies of psychosocially modelled educational interventions and youth worker deployment in young people with T1DM. We found a limited number of studies that incorporated some elements of the YES programme but none that shared the same theoretical approach with strategies explicitly focused on the same target behaviours.^30–33^ Hence, the YES programme is a distinct, codesigned and theoretically modelled intervention, with novel components. A pilot evaluation of three early implementations of the YES programme that included 26 participants (mean age 18±1.7 years) showed it to be impactful on glycaemic control, with 46% (n=12) of participants achieving a clinically significant reduction in their HbA1c (≥5.5 mmol/mol or 0.5% DCCT/Diabetes Control and Complications Trial units) at the 12-month follow-up. Programme uptake was 34% (n=26) with 96% (n=25) programme completion, which is similar to other programmes targeting young people.^25^ Qualitative findings suggested that the programme had a positive psychosocial impact resulting in increased diabetes engagement and activation. The following active ingredients of the programme were also identified: social learning, peer support, relational care/support and experiential learning. The youth worker was also highlighted by participants as being important in engaging them with the programme.

In the study outlined in this protocol, we propose to undertake a feasibility trial of the YES programme, with an integrated process evaluation. We have modelled the active ingredients of the programme to target outcomes related to psychosocial well-being, diabetes self-management activation and glycaemic control. In the process evaluation, we will examine methodological, procedural and clinical uncertainties around the YES programme,^30–32^ including: (1) estimates of likely recruitment and retention rates; (2) feasibility and acceptability of data collection instruments and data collection procedures; and (3) feasibility and acceptability of the programme. In line with standard feasibility study objectives,^31^ we will not be testing the effect of the programme on outcomes, although we will estimate the impact of the programme on outcomes, to: consider the feasibility of progression to a definitive trial; and to inform a sample size calculation for such a trial. There will also be a mixed method element to the process evaluation,^32^ to explore: (1) intervention acceptability; (2) the intervention experience and its impact on physical, mental and social well-being; and (3) parents’ perspectives on the programme. These findings will be integrated to formulate potential mechanisms of change following programme exposure.

### Aims and objectives

The aims of study are to: test the feasibility of the YES programme in terms of programme acceptance, implementability, recruitment and completion; and to estimate its efficacy in relation to clinical, psychosocial and healthcare outcomes. The study objectives are to:

- Evaluate and optimise the research process: recruitment, willingness to be randomised, consent procedures and data collection.
- Evaluate our ability to assess the programme’s impact on self-management, patient activation and clinical and psychosocial outcomes (optimising outcome selection and estimating the level effect).
- Establish the reach, fidelity, utility, feasibility, appropriateness, unintended consequences and sustainability of the YES intervention.
- Identify and explore the experiences of programme participants and those delivering the intervention.
- Optimise the intervention components, procedures and outcome measures.
- Obtain estimates of outcomes to inform the sample size calculation for a larger clinical evaluation study.

## Methods and analysis

### Study design

**Figure 1:**
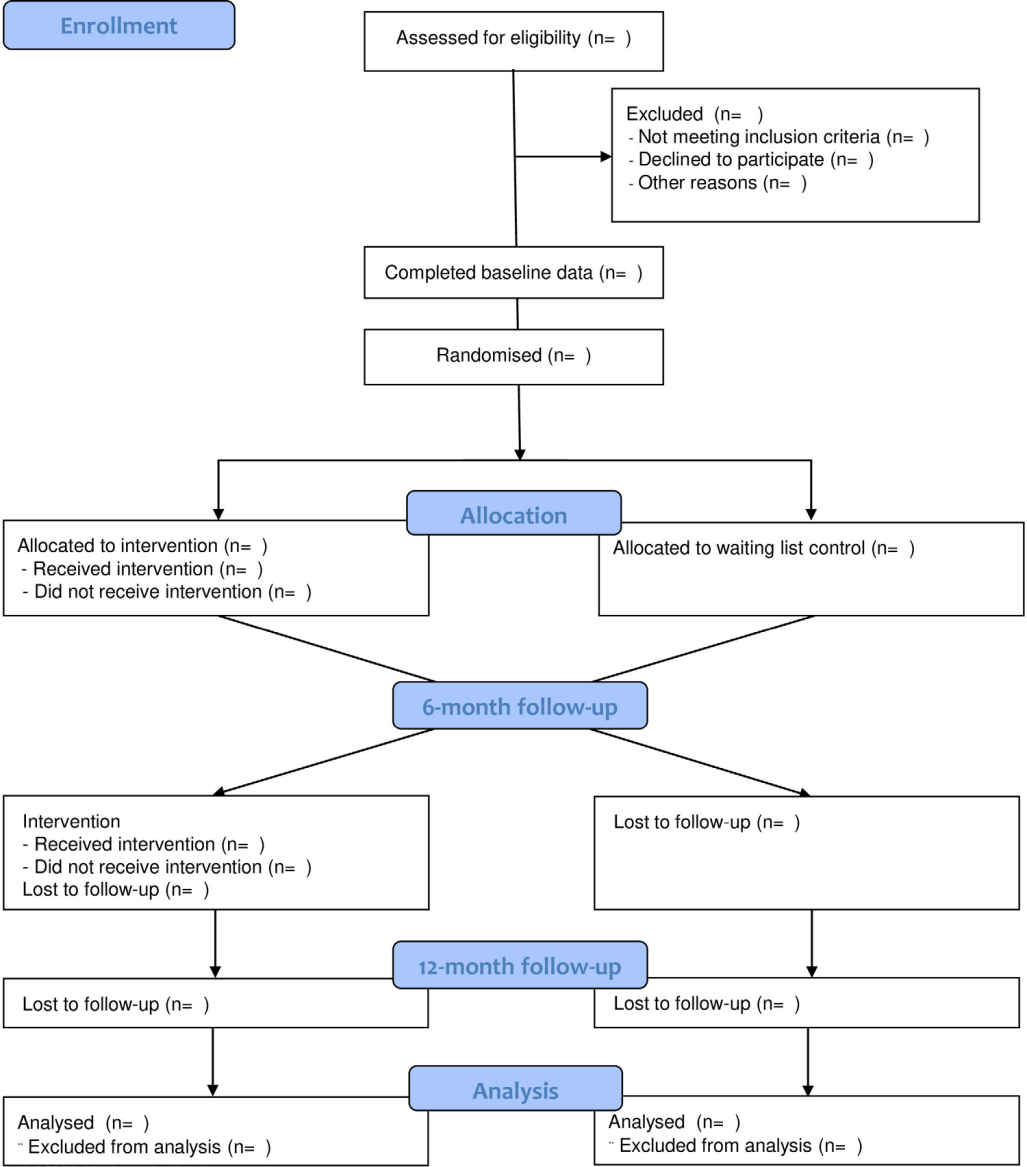
Consort diagram for the Youth Empowerment Skills programme feasibility study.

The study has been designed as a feasibility waiting-list randomised controlled trial (RCT) with an integrated process evaluation. Participants will be randomised to YES exposure (phase 1) or a waiting list control and followed up for 6 months. At the 6-month follow-up point, control participants will then be exposed to YES, in a phase 2 exposure. Both groups will be subsequently followed up for a further 6 months. This will ensure that all eligible young people will have the opportunity to participate in the programme while maintaining a controlled structure to YES exposure that will allow us to explore differences. Conducting an additional 6-month follow-up after phase 1 will also enable us to assess how sustainable the intervention is over a longer time period. The adoption of a waiting list design is important as it overcomes the ethical dilemma of denying control participants the opportunity to benefit from the programme. The study design is presented schematically in figure 1.

We have set the following progression criteria for a subsequent definitive trial: achieve minimum 30% recruitment of eligible participants; and ≥80% completion of final follow-up. We will also assess the impact of the intervention ingredients on target behaviours, including activation of self-management, engagement in diabetes healthcare and improved psychosocial functioning. These effects, alongside impact on glycaemic control, will be used to consider whether there is adequate potency in the programme and will determine whether a definitive trial will be conducted.

### Setting

The study will be conducted in South London, UK, recruiting participants from the diabetes centres of five hospitals, providing care to the target population. These sites have been selected to ensure sociodemographic diversity, with a significant proportion of patients from lower socioeconomic backgrounds and from Black Asian and Minority Ethnic populations. The study is planned to start on 1 June 2021, and finish 31 March 2023.

### Participants and sample size

We aim to recruit 50 young people and randomise half to the phase 1 programme exposure, and half to the phase 2 exposure. The inclusion criteria will involve:

- Age 14–19 years.
- Diagnosis of T1DM.
- Suboptimal glycaemic control (HbA1c>69 mmol/mol or 8.5%).^3^
- No current or planned attendance of other structured education programmes.

Participants will be excluded if they: have a severe physical or mental illness, unstable retinopathy, significant learning difficulties; are unable to communicate in English; or are pregnant. Participants meeting the inclusion criteria will be recruited opportunistically at the participating centres through clinical referrals and patient database searches. Based on recruitment to the pilot study, we estimate that we will be able to recruit >30% of eligible patients. To increase engagement, we will run the full study, including programme occurrences and follow-up sessions, during school holidays as this was identified by young people as the optimal period for running the YES programme.

As a feasibility evaluation, the study is not statistically powered to estimate a clinical effect. Instead, we aim to estimate the effect-size/SD of outcomes to formulate a power calculation for a definitive trial. National Institute for Health Research guidance indicates that samples of 24–50 are adequate for this purpose.^33–35^ We have inflated our sample to allow for an estimated 20% drop-out rate.

### Intervention and control conditions

The intervention is the YES programme as previously described. The programme exposure is sequenced as follows: (1) initial contact from the youth worker introducing the YES intervention; (2) recruitment to the study by researchers; (3) attendance at three all-day YES sessions in non-healthcare settings (groups of 10); (4) postprogramme networking through social media and (5) follow-up events facilitated by the youth worker. Sessions will be led by a peer-educator and a health professional and delivered face to face. Participants in the waiting-list control arm of the study will receive usual care following initial randomisation and will be exposed to the YES intervention at 6 months (phase 2 exposure). Usual care in this patient population typically involves outpatient consultations. To minimise potential external confounding factors, we will monitor any other educational interactions that participants might experience during the follow-up period in both groups.

### Randomisation

Participants will be randomised to either the phase 1 YES exposure or to the waiting list control (for phase 2 exposure), using computer-generated randomisation in blocks of 20. This will be conducted by a member of the study team not involved in recruitment. As the intervention is open, blinding is not possible. The waiting list design will help to maintain young people’s motivation to participate in the phase 1 and 2 allocations. As part of our feasibility work, we will consider how acceptable randomisation is to young people with diabetes. Young people’s lives are quite dynamic in this period with frequent changes such as moving schools starting further or higher education, or employment. Hence, while we will explain why randomisation is helpful to us, but if young people decline randomisation they will be offered a preference for when they join the programme.

### Outcome measures

As a feasibility study, the objective is to optimise outcome selection and determine outcome effects prior to a definitive RCT. The primary outcome to assess the potential clinical effect of the programme will be HbA1c and we will examine: (1) the number of participants achieving clinically significant reductions of 5.5 mmol/mol or more and mean differences between groups over time, and (2) the SD of the mean difference between the intervention and control group in HbA1c at 6 months; for the control group, the SD of the mean difference between HbA1c at 12 months and preexposure (phase 2) and for the phase 1 intervention group, the SD of the mean HbA1c difference between post (6 and 12 months) and pre-exposure. While HbA1c is a proxy measure of complication risk, it is the most commonly adopted measure for determining the short to medium-term impact of an intervention and is the recommended measure from the National Institute for Health and Care Excellence for assessing the clinical and health economic benefits of diabetes interventions. In addition, our preliminary work and logic model show that the programme can mediate health behaviours by impacting on the psychosocial factors that can mediate self-management activation leading to improved glucose control. Hence, we will also assess the psychosocial and behavioural impacts of the programme, using the following secondary outcome measures:

- Number of participants with 20% increase in frequency of blood glucose monitoring.
- Insulin adherence measured by self-report scale.
- Self-Management of Diabetes in Adolescence Scale.^36^
- Confidence in managing own healthcare (Confidence in Diabetes Self-Care Scale.^37^
- Diabetes Quality of Life Instrument-adapted for youth.^38^
- Illness perception (Brief Illness Perception Questionnaire).^39^
- Emergency care events (ambulance call-outs, accident and emergency attendance or hospitalisations).
- Hypoglycaemia (blood glucose<3.5 mmol/L) and severe hypoglycaemic events (requiring third party assistance).
- Weight.
- Intervention Appropriateness Measure.
- Feasibility of Intervention Measure.
- Acceptability of Intervention Measure.

For each outcome, we will assess differences in preexposure and postexposure, as well as differences between control and intervention groups.

### Study procedure

Potential participants will be recruited from the participating diabetes centres. Since it can be challenging to engage young people with T1DM, especially those from deprived and ethnically diverse groups, a youth worker role will be incorporated into the recruitment process to identify potential participants and support them in considering participation in the study. Young people will then be screened for eligibility and consented. Parental consent will also be gained from those aged <16 years of age. We will ask eligible young people who decline participation to give a reason for their decision. The participants will then be randomly allocated as previously described.

### Data collection and storage

Data (questionnaires, body weight and medical-record data) will be collected at baseline, then again at 6 and 12 months. Baseline data will also include: sociodemographics; education/employment; and diabetes self-management behaviours including insulin use and blood glucose testing. Patient records will be examined to collect data on: HbA1c; hospitalisations and emergency care; diabetes education attendance; screening attendance; and attendance at appointments. For baseline, this will be all events in the preceding 12 months, and postexposure will be for the 12 months following exposure (or 6 months’ post exposure for those exposed at phase 2). Analysis will be on-treatment, as the primary aim of this study is to assess feasibility. Data will be coded and cleaned by statisticians and stored on an encrypted shared drive.

### Data analysis

The data will be analysed to determine the proportion of patients achieving the progression criteria; and the target reduction in HbA1c from baseline to follow-up (6 and 12 months), which will be estimated in both intervention (YES) and control groups. We will also estimate the SD of the mean difference between the intervention and control group in HbA1c at 6 months; for the control group, the SD of the mean difference between HbA1c at 12 months and pre-exposure (phase 2) and for the phase 1 intervention group, the SD of the mean HbA1c difference between post (6 and 12 months) and pre-exposure. These data will help inform the sample size calculation for a definitive trial.

### Process evaluation

The process evaluation has been designed in accordance with the Medical Reseach Council's (MRCs) process evaluation model for complex interventions,^32^ aiming to explore the setting, implementation and mechanisms of action of the programme to enable interpretation of the observed outcomes. Our process evaluation will also use an established taxonomy of outcomes to be evaluated for understanding implementation.^40 41^ Following this taxonomy, we will assess the reach, fidelity; its perceived acceptability, appropriateness and feasibility; unintended consequences; and potential sustainability considerations. In addition, we will elicit potential mechanisms of action, including changes in behaviours, and how they relate to programme delivery and outcomes, using the Capability, Opportunity, Motivation to perform Behaviour (COM-B) and Behaviour Change Wheel (BCW) frameworks.^42 43^ Further, we will apply the Consolidated Framework for Implementation Research (CFIR) to study how the programme can be optimally embedded within routine diabetes care (ie, to capture systematically implementation and adoption drivers/barriers); and to optimise the design of a subsequent trial.^44^

The process evaluation will involve a mixed-method approach, including use of validated brief implementation outcome surveys^45^ (to be delivered to both young people with diabetes and health providers involved in YES) and one-to-one interviews with young people, their relatives and healthcare professionals. The data collection tools and analytic models have been designed with reference to the BCW framework^43^ and to provide the data for CFIR^44^ analysis considering the barriers and facilitators to the intervention. To ensure relevance and feasibility, implementation data collection tools will be refined with the study patient and public representative groups. Semistructured interviews exploring the implementation outcomes and programme impact within the first 3 months of the completion of the programme will be conducted with a random subsample of participants (n=12 from each arm of the study, 4 in each centre), including participating young peoples’ relatives (n=10); and the healthcare professionals involved in the delivery of the programme (n=6). All interviews will be digitally recorded and transcribed verbatim in preparation for analysis using thematic analysis,^46^ mapping the data using the BCW^43^ and CFIR^44^ frameworks.

To assess perception of how appropriate, acceptable and feasible the programme is to young people and healthcare professionals, we will use previously validated short pragmatic surveys.^45^ This will allow quantitative assessment against the study end-points. We will also undertake:

- An audit of the number of eligible young people that decline participation, considering their clinical and sociodemographic profiles.
- An audit of intervention fidelity using an adherence checklist based on the YES protocol.
- Exit questionnaires with attending young people to rate programme satisfaction and utility; and to identify three strengths, weaknesses and areas for improvement.

The audit and questionnaire data will be analysed to examine the utilisation and satisfaction with the programme. Intervention fidelity will be assessed from the audit data detailing the delivery of the different intervention components. We will also monitor control conditions in the first 6 months to consider whether any extraneous interventions or clinical changes occurred that might impact on the observed outcomes. While the study will be recruiting participants from five diabetes centres, we do not anticipate significant site level effects on outcomes as the intervention will be delivered by the same clinicians and peer educators throughout, following the YES curriculum. However, we will monitor any variations in the general care for each centre, or any changes in the care provided to the target population during the follow-up period.

### Health economic evaluation

While a full health economic analysis is beyond the scale of this feasibility study, we will collect data as part of the process evaluation to inform health economic analysis for a future definitive trial. The analysis will have three elements:

1. A full economic costing of the programme delivery costs.
2. An audit of medical records to consider healthcare utilisation and in particular impact on high-cost emergency care episodes.
3. Modelling cost benefits by considering the risk of long-term complications based on impact on glycaemic control using established health economic assumptions.^47^

### Review and optimisation event

In the final phase of the study, we will organise a YES review event with a purposively selected group (age and gender) of participants (n=16), their family members or partners, health professionals from participating services and the research team. The aim of the event will be to share study findings and collate feedback about the programme through facilitated round table discussions, in which participants will be encouraged to offer ideas to further improve the YES programme. Following this event, and based on an integrated assessment of the data collected, the intervention protocol and outcome measures will be revised for use in a definitive trial.

### Patient and public involvement

A patient and public involvement (PPI) group consisting of young people who had previously attended the YES programme contributed to the development, design and conduct of the study (01/2018 to date), including: outcome measure selection; topic guide content for interviews; and formulating the information sheets and consent forms for ethics. In terms of outcome measures, the group advised us that only 3–4 questionnaires should be used as any more would be too burdensome and that this would affect quality of responses received. We showed the group 9 questionnaires which we related to the target psychosocial constructs and behaviours. They felt that many of the measures were too personal, confusing or distressing. Hence, we removed measures for: diabetes distress; diabetes stigma; and depression. We may, however, pick-up these themes in the interviews. We have recently expanded the PPI group to include some young people who have not attended the YES programme; and we have set up an additional group of parents of young people with T1DM. These groups will support the research team in the analysis, write-up and dissemination of study findings; and help in the process of optimising the programme and study procedures for a future trial.

PPI has also been important in helping prepare the study in relation to COVID-19 restrictions. Prior to starting the study, we conducted a survey of 74 young people with diabetes to explore whether they would be willing to meet in a group once the restrictions had lifted. Over half of the respondents (n=38) identified, they were likely or very likely to attend a face-to-face education group or activity for young people with diabetes if it were offered to them. Three-quarters of the respondents (n=56) said that their views on attending YES had not been impacted by the pandemic.

### Trial management

The study conduct and procedures will be overseen by an advisory board, comprising clinical stakeholders, the funder and PPI representatives. As a feasibility study of an intervention without hazard or potential for harm, there are no provision for stopping guidelines nor a specific data monitoring committee. Trial participants will receive follow-up support post trial as part of their routine care. Any adverse events will be recorded and reported, and the study will be compliant with the hospital’s safeguarding policy. Any changes to the protocol will be discussed with the study advisory board and reported to the research ethics committee, funder and trial participants. The final trial dataset will be accessed by the trial statisticians and researchers.

## Discussion

In this paper, we have described the protocol for a feasibility study to assess a complex psychoeducational intervention for young people with T1DM. As we have highlighted, there is a need to develop and test new educational approaches for young people with T1DM, which take account of the significant psychological and social challenges they experience in this period of their lives. The YES programme was codesigned with young people and has been theoretically modelled to target the challenges relevant to them, in order that they can adapt to living with diabetes. The study we have outlined here will help us establish whether the active mechanisms within the programme connect with how young people engage with their diabetes self-management as a condition for improving their glycaemic control, thereby reducing the risk of future complications. The study will also assess both quantitatively and qualitatively how the programme effects young people’s orientation to their diabetes (positive or negative) psychologically and socially.

In conducting a trial of a clinical intervention, such as YES, it is important to consider how the intervention is experienced by those receiving and delivering it. Following the MRC model for complex interventions,^32^ the process evaluation will assess the feasibility of the YES programme in terms of acceptance, implementability, recruitment and completion. Application of the COM-B^42^ and BCW^43^ frameworks will allow us to elicit/confirm the potential mechanisms of action and how they relate to programme delivery. This analysis will also help us optimise the intervention components and outcome measures. Further, use of the CFIR framework to delineate barriers and drivers to YES implementation and Proctor *et al*’s^40^ taxonomy of implementation outcomes will allow systematic study of implementation aspects of YES, with both young people, their parents and T1DM care providers. These aspects of the study will be important in understanding how the intervention works in the context of its delivery and how it might be enhanced.^40^ This evidence will be needed for the design of a subsequent definite trial of YES: based on the data that we collect on YES delivery, and if the implementation data collection of the current study, including both scales and qualitative data, is successful, it follows that a definite trial of YES may take a ‘hybrid’ format. Hybrid effectiveness-implementation studies include both clinical and implementation outcomes and evaluate both clinical effectiveness and implementation success of different strategies to deliver YES.^48^ Such designs are efficient, in that they offer both clinical evidence and, simultaneously, evidence on what implementation strategies and support needs to be in place for YES to be scaled successfully outside the trial centres. The current study will provide the early information required for such a hybrid design to be applied.

Given the inherent complexity of the target population for the YES programme, running a clinical trial in this population will be challenging. Hence, the feasibility questions posed by the study in respect of recruitment, retention and data completeness will be important. We also hope that the qualitative work within the process evaluation will also allow us to learn how the research approach can be optimised prior to a full trial. A key consideration in relation to this will be the study design, particularly whether randomisation and the use of a waiting list model will be feasible. There is a growing recognition that conducting clinical trials in adolescents and young adults is challenging, with the suggestion that alternative more adaptive research models are needed.^49^ While we have sort to address these challenges with the waiting list design and including the option for preference should participants decline randomisation, such adaptations can introduce bias in estimating observations.^50^ While we cannot fully assess these biases in this study, it will be important to consider whether, for example, those who decline randomisation and express a preference for either phase 1 or phase 2 exposure show stronger of weaker effects compared with those randomised. Following completion of the study, we will use the YES review event and our PPI groups to help us consider how best to design a definitive study should we meet our progression criteria.

## Ethics and dissemination

The study described in this protocol has been reviewed and given favourable opinion by the London—Camden and Kings Cross Research Ethics Committee (REC number: 21/LO/0231; IRAS number: 279877) on 20 April 2021. Informed consent will be sought from all research participants for this study; a copy of the consent form (for participants >16 years of age) can be found in the online supplemental file 1 and a copy of the assent form (for participants <16 years of age) in the online supplemental file 2. The study will be conducted in accordance with Good Clinical Practice and recommendations for physicians involved in research on human subjects adopted by the 18th World Medical Assembly, Helsinki 1964 and later revisions. The study will produce key information to the stakeholders on the planning, funding and implementation of the interventions under investigation. Hence, the findings will be disseminated through peer-reviewed journals, relevant national and international conferences, as well as educational events at individual hospitals to ensure that they are brought to the appropriate stakeholders. We will also disseminate the findings to young people with diabetes and their families by sharing a lay summary of the findings on the Diabetes UK website and via relevant social media groups.

10.1136/bmjopen-2022-062971.supp1 Supplementary data

10.1136/bmjopen-2022-062971.supp2 Supplementary data

## Supplementary Material

Reviewer comments

Author's manuscript
