# Supplementary material for: Protocol for a feasibility study and process evaluation of a psychosocially modelled diabetes education programme for young people with type 1 diabetes: the Youth Empowerment Skills (YES) programme
Source: BMJ Open. 2022 Jun 8;12(6):e062971. doi: 10.1136/bmjopen-2022-062971 (PMC9185576; doi:10.1136/bmjopen-2022-062971)
Supplement: Supplementary data [file bmjopen-2022-062971supp001.pdf]

NHS
Guy's and St Thomas'
NHS Foundation Trust

KING'S College LONDON

IRAS ID:	279877
Centre Number:	
Participant Identification Number for this study:	
**CONSENT FORM FOR YOUNG PERSON ≥16 (PART A)**	
Title of Project:	A feasibility study and process evaluation of a psychosocial education programme for young people with type 1 diabetes - the Youth Empowerment Skills (YES)
Name of Researcher:	

Initial each box

1. I have read the *Participant Information Sheet* dated................ I **understand what the research project is about**, what will happen if I take part and I have had all my questions answered. ☐
2. I understand that I do not have to take part, and I know that I can stop taking part at any time without giving a reason. I know that **this will not make a difference to the care I receive from my diabetes team.** ☐
3. I give permission for researchers from King's College London to **look at my medical notes**. This will only be to find the information relevant to the above research project (as mentioned in the participant information sheet), and **only for the duration of the research,** which will end in August 2022**.** ☐
4. I agree to fill in **questionnaires** twice during the research project. ☐
5. I agree to take part in an **interview** (if asked) about the YES programme. The interview will be **audio-recorded** but will only be listened to by the researcher. Some of the things I say in the interview may be written about in research reports, but this will be **anonymous** (no one will be able to know who said it). ☐
6. I understand that people will be **chosen at random to have an interview**, and that I may not be offered one. ☐
7. I understand that information I give will be kept **confidential** (private) and stored in a safe place for up to 12 years after the project ends. ☐
8. I understand the **limits to confidentiality** as stated in the *Participant Information Sheet*. For example, if the researchers are worried about my safety or the safety of others they may tell someone else. ☐
9. I understand that if I leave the study early, the researchers will **still use the information** I have already given them. However, this information will **not have my** ☐

Version 1.2 13/04/21 IRAS no. **279877**

NHS Guy's and St Thomas' NHS Foundation Trust

KING'S College LONDON

name on. Any information about me that includes personal details will be removed from the research project and archived (stored).

10. I understand that if I leave the study early, I will be asked if I am willing to take part in an **interview**, so the researchers can develop an understanding of what things might make a young person leave the study. I do not have to participate in this if I do not wish to. ☐

11. I understand that, for **auditing and monitoring purposes**, data collected during the research project may be looked at by people from King's College London, from regulatory authorities, or from the NHS Trust, where it is relevant to my taking part in the study. I give permission to these people to look my records for this reason. ☐

12. I understand that my **usual hospital diabetes team** will know that I am taking part in this research project. ☐

13. I understand that my **general practitioner (GP)** will know that I am taking part in this research project. ☐

14. **I agree to take part in the above research project.** ☐

Please sign and date the following section

______________________	____________	____________________
Name of participant	Date	Signature
______________________	____________	____________________
Name of person taking consent	Date	Signature

One copy for the participant, one copy for the researcher and one copy for the medical notes

Version 1.2 13/04/21 IRAS no. **279877**

IRAS ID:	279877
Centre Number:	
Participant Identification Number for this study:	
Title of Project:	A feasibility study and process evaluation of a psychosocial education programme for young people with type 1 diabetes - the Youth Empowerment Skills (YES)
Name of Researcher:	

ADDITIONAL CONSENT FORM (PART B)

Part B asks if you agree for us to keep your contact details after this research project is finished. This is so we can contact you about future research you might want to take part in. It is fine if you do not want us to keep your contact details for this purpose. You **do not** have to sign Part B to take part in this research project.

If you choose not to sign Part B, we will remove your name and contact details from our contacts list when this research project is finished, and we will not contact you about other research in the future. If you do sign Part B, you can still change your mind and ask us to remove your name and contact details from our contacts list **at any time**.

Initial each box

1. I agree that researchers at King's College London can keep my name and contact details on the project contacts list for_____years after this research project is finished. ☐
2. I understand that my name and contact details will be saved on a computer at King's College London and will be protected with a password. Only the researchers directly involved in this research project will have the password to access this information. ☐
3. I agree that these researchers from King's College London can use this information to contact me in the future. They will only contact me to let me know about other research that I can take part in **if I want to**. ☐
4. I understand that my name and contact details will **not** be used for any reason except that described above (number 3.). ☐
5. I understand that I can ask for my name and contact details to be removed from the ☐

contacts list at King's College London at any time. I know who to contact if I want to remove my details. I understand that researchers will not be able to contact me in the future if I remove my details.

6. I understand that if I do not want King's College London to keep my name and contact details on the contacts list after this project is finished, or if I ask for them to be removed at a later date:
   - This will **not** affect my involvement in the above research project.
   - This will **not** affect the care I receive from my diabetes team.

Please sign and date the following section

___________________________	______________	_________________________
Name of participant	Date	Signature
___________________________	______________	_________________________
Name of person taking consent	Date	Signature

One copy for the participant, one copy for the researcher and one copy for the medical notes
