# Supplementary material for: Protocol for a feasibility study and process evaluation of a psychosocially modelled diabetes education programme for young people with type 1 diabetes: the Youth Empowerment Skills (YES) programme
Source: BMJ Open. 2022 Jun 8;12(6):e062971. doi: 10.1136/bmjopen-2022-062971 (PMC9185576; doi:10.1136/bmjopen-2022-062971)
Supplement: Author's manuscript [file bmjopen-2022-062971.draft_revisions.pdf]

BMJ Open is committed to open peer review. As part of this commitment we make the peer review history of every article we publish publicly available.

When an article is published we post the peer reviewers' comments and the authors' responses online. We also post the versions of the paper that were used during peer review. These are the versions that the peer review comments apply to.

The versions of the paper that follow are the versions that were submitted during the peer review process. They are not the versions of record or the final published versions. They should not be cited or distributed as the published version of this manuscript.

BMJ Open is an open access journal and the full, final, typeset and author-corrected version of record of the manuscript is available on our site with no access controls, subscription charges or pay-per-view fees (http://bmjopen.bmj.com).

If you have any questions on BMJ Open's open peer review process please email info.bmjopen@bmj.com

BMJ Open

Protocol for A Feasibility Study and Process Evaluation of a Psychosocially Modelled Diabetes Education Programme for Young People with Type 1 Diabetes: the Youth Empowerment Skills (YES) Programme

Journal:	*BMJ Open*
Manuscript ID	bmjopen-2022-062971
Article Type:	Protocol
Date Submitted by the Author:	21-Mar-2022

Complete List of Authors:	Kariyawasam, Dulmini; Guy's and Saint Thomas' Hospitals NHS Trust, Diabetes<br>Soukup, Tayana ; King's College London, Health Service and Population Research Department; Imperial College London<br>Parsons, Judith; King's College London Florence Nightingale School of Nursing and Midwifery, Adult Nursing<br>Sevdalis, Nick; King's College London<br>Baldellou Lopez, Maria ; King's College London<br>Forde, Rita; King's College London, Faculty of Nursing, Midwifery and Palliative Care<br>Ismail, Khalida; King's College London<br>Jones, Marie; Guy's Hospital<br>Ford-Adams, Martha; King's College Hospital NHS Foundation Trust, Diabetes Department<br>Yemane, Nardos; Guy's and St Thomas' NHS Foundation Trust, Diabetes Department<br>Pender, Siobhan; Guy's and Saint Thomas' NHS Foundation Trust Neurology Department<br>Thomas, Stephen; Guy's and St Thomas' Hospitals NHS Trust, Diabetes Department<br>Murrells, Trevor; King's College London, National Nursing Research Unit<br>Silverstien, Alex; King's College London<br>Forbes, Angus; King's College London, Division of Care in Long-term Conditions

Keywords:	DIABETES & ENDOCRINOLOGY, General diabetes < DIABETES & ENDOCRINOLOGY, General endocrinology < DIABETES & ENDOCRINOLOGY

SCHOLARONE™
Manuscripts

Protocol for A Feasibility Study and Process Evaluation of a Psychosocially Modelled Diabetes Education Programme for Young People with Type 1 Diabetes: the Youth Empowerment Skills (YES) Programme

Dulmini Kariyawasam,[1] Tayana Soukup,[2-5*] Judith Parsons,[6] Nick Sevdalis,[2] Maria Baldellou Lopez,[2] Rita Forde,[6] Khalida Ismail,[7] Marie Jones,[1] Martha Ford-Adams,[8] Nardos Yemane,[1] Siobhan Pender,[1] Stephen Thomas,[1] Trevor Murrells,[6] Alex Silverstien, Angus Forbes[6]

Author affiliations

[1] Diabetes Department, Guy's and St Thomas' NHS Foundation Trust, London, UK

[2] Centre for Implementation Science, Health Service and Population Research Department, Institute of Psychiatry, Psychology and Neuroscience, King's College London, UK

[3] Institute of Global Health Innovation, Faculty of Medicine, Imperial College London, UK

[4] School of Health & Sport Sciences, University of Suffolk, UK

[5] Department of Epidemiology and Environmental Health, The State University of New York at Buffalo, USA

[6] Division of Care in Long-term Conditions, King's College London, UK

[7] Department of Psychological Medicine, Institute of Psychiatry, King's College London, UK

[8] Diabetes Department, King's College Hospital, London, UK

Full address for correspondence

*Dr Tayana Soukup, Centre for Implementation Science, Health Service & Population Research Department, King's College London, 16 De Crespigny Park, London SE5 8AF. Email: tayana.soukup@kcl.ac.uk Telephone Number: +44207 848 0272

Word count: 4538/4000

For peer review only

ABSTRACT (268/300w)

Introduction. Adolescence is a challenging period for young people with type 1 diabetes, associated with worsening glycaemia and care disengagement. Educational interventions in this period tend to focus on diabetes-specific skills, with less emphasis on the psychosocial challenges associated with diabetes experienced by young people. To address this limitation, we co-designed with young people a psychosocially modelled programme of diabetes education, named 'Youth Empowerment Skills' (YES). The programme aims to facilitate a positive adaptation to life with diabetes and engagement with diabetes care through peer-based learning, immersive simulations, and support from an outreach youth worker. Here we present a protocol for a feasibility study of the YES programme.

Methods and analysis. The study was designed following the Medical Research Council Complex Intervention Evaluation Framework to: test the feasibility (acceptance, implementability, recruitment and completion) of the YES programme; and estimate its efficacy in relation to metabolic and psychosocial outcomes. The study will take place in diabetes centres serving socio-culturally diverse populations. We will conduct a feasibility RCT (waiting-list design) with integrated process evaluation. Fifty young people with type 1 diabetes (aged 14-19 years) will be randomly allocated to either the YES intervention or a waiting-list control. Randomisation acceptability will be assessed with provision for a preference allocation. Outcomes will be evaluated at 6 months, at which point the waiting list participants will be exposed to the YES programme with further follow-up to 12 months. A simultaneous process evaluation will use a mixed-methods approach collecting qualitative and quantitative data. Study findings will be used to optimise the intervention components, outcome measures and recruitment methods to inform a subsequent definitive trial.

Ethics and dissemination. The protocol has ethical approval from the UK Health Research Authority (approval IRAS project ID:279877). Findings will be disseminated in multiple formats for lay and professional audiences.

Key words: Type 1 diabetes, young people, psychoeducational programme

Trial registration number: Clinicaltrials.gov identifier NCT04670198

For peer review only - http://bmjopen.bmj.com/site/about/guidelines.xhtml

Strengths and limitation of the study

- The study will test a novel co-designed psychoeducational intervention for young people with Type 1 diabetes designed to help promote a positive emotional and social adaptation to a life with diabetes.
- An integrated process evaluation within the study will develop our understanding of how to enhance study procedures and gain insights into programme implementation for future studies.
- The randomised waiting-list design allows for the comparison of the intervention and usual care exposures without the ethical implications of not providing the intervention to the control group.
- The target population for this study can be resistant to healthcare interventions and have significant school and social commitments, hence the study will consider recruitment feasibility and the acceptability of randomisation versus preference for group allocation.

For peer review only

For peer review only - http://bmjopen.bmj.com/site/about/guidelines.xhtml

INTRODUCTION

Impact of T1DM on young people

Type 1 diabetes (T1DM) in adolescence and early adulthood can be a very challenging period in the lives of young people. During this period young people can begin to dissociate from their diabetes, leading to a decline in their glycaemic control and an increased risk of acute events such as recurrent diabetic-keto-acidosis (DKA).[1-5] Data from the most recent National Paediatric Diabetes audit in the United Kingdom showed that only 10% of young people aged 14-19 years achieved a glycated haemoglobin (HbA1c) within the current target range of ≤48mmol/mol(6.5%); and a fifth had a HbA1c >75mmol/mol(9%) which is associated with a high risk of diabetes complications.[3]

Furthermore, adolescence and early adulthood is also a period when young people are forming their personal and social identities, and diabetes can have a detrimental impact on their psychosocial development.[6,7] During this period, young people can develop negative emotional constructs about diabetes, as they see their diabetes as an impediment to social relationships and life opportunities.[8] Consequently, some young people may develop maladaptive coping strategies such as avoidance (omitting insulin and reducing their glucose monitoring), or psychological morbidities (fear, anxiety, depression and disordered eating behaviours).[9,10,11] Young people with diabetes can also feel stigmatised,[12] further driving negative coping strategies (e.g., not using insulin in public or running glucose levels high to avoid hypoglycaemia).

During this period young people are also developing their personal autonomy, which can lead to conflicts with parents who may find allowing the young person to take control over their diabetes self-management behaviours and health management challenging.[13] Relationships can become emotionally charged with conflict, excess vigilance and distress, difficulties that can exacerbate disengagement and are associated with poorer glycaemic control.[14,15] Negative interactions with diabetes health professionals have also been identified as contributing to young people disengaging from their care.[16]

For peer review only - http://bmjopen.bmj.com/site/about/guidelines.xhtml

Current care models for young people with T1DM

Developing effective diabetes care for young people with T1DM is challenging. The transition from child to adult care has long been identified as a period when young people can disengage from their care, despite much innovation.[17-22] In part this is attributable to the failure of current care provision to adequately integrate the needs or life contexts of young people within that care. Young people with T1DM need support to help them develop the psychosocial skills necessary to transition positively into an adult life with diabetes, and to enable them to engage productively with health care providers. However, the main focus in diabetes guidelines is on intensifying glucose levels with diabetes technologies and structured education.[23,24] Therefore, most current education programmes for young people aim to enhance self-management skills such as carbohydrate counting and insulin-dose-adjustment, to help optimise glucose levels; rather than addressing how to integrate living with diabetes in relation to the wider interpersonal and social challenges that they experience in this period. Hence, while these programmes are generally effective in the adult diabetes population, young people are less likely to participate in these programmes. A recent study of 227 young people identified that the main reasons for their non-participation was a lack of time and a preference for managing their diabetes on their own.[25] This indicates the need to reconsider both the type of educational support provided and how it is delivered to young people. While some programmes have been adapted for younger people, there is still an emphasis on technical aspects of diabetes,[26] rather than psychosocial adjustment.

Therefore, new educational programmes are required for young people, to ensure that what they learn about managing their diabetes is contextualised with learning to live with diabetes in relation to their social and emotional development. It is also important to explore innovative methods of delivering education to young people with T1DM to make programmes more relevant, attractive and accessible to them.

The Youth Empowerment Skills programme for young people with T1DM

The Youth Empowerment Skills (YES) programme is a novel psychoeducational intervention, which was developed through a co-design process with young people with the fundamental goal to overcome some of the challenges reviewed above. The co-design process sort to build the programme so that it addressed many of the previously identified challenges experienced

For peer review only - http://bmjopen.bmj.com/site/about/guidelines.xhtml

by young people with diabetes. The programme uses 3 well-established psychological theories, social learning theory,[27] self-regulatory theory[28] (personal identity) and dual-process theory.[29] These theories have been integrated into a learning model addressing: cognitive factors (knowledge, experiences, thinking processes and attitudes); environmental factors (exploring social norms and external factors that mediate behaviour); and behavioural factors (developing skills and self-efficiency, with practice, observation and rehearsal). This underpinning theory is delivered using different learning techniques: experiential and group-based learning; immersive simulations with scenarios such as treating an episode of severe hypoglycaemia or ketosis; and learning together in adventurous activities. The programme curriculum comprises the following sessions:

- Diabetes as a part of daily life;
- The psychological impact of diabetes;
- Staying safe while being away from home (parties/alcohol/drugs and diabetes);
- Immersive simulations (hypoglycaemia, DKA, impaired physical function);
- Reverse role-playing of health consultations (how to get more out of a consultation);
- Relationships (peers/partners/family);
- Attitudes towards food, weight and eating out;
- Getting to know your body (foot-care/eye screening/sexual health);
- Exercise and diabetes (activity-based sessions, e.g. rock climbing);
- How to prevent diabetes emergencies.

The programme is delivered by a peer educator with T1DM, health professionals and youth workers. The youth workers also do outreach work to identify potential participants and encourage them to attend. The programme is fully manualised and Quality Institute for Self-Management Education (QISMET) accredited.

As part of the programme's development, we undertook a systematic review to identify studies of psychosocially modelled educational interventions and youth worker deployment in young people with T1DM. We found a limited number of studies that incorporated some elements of the YES programme but none that shared the same theoretical approach with strategies explicitly focussed on the same target behaviours.[30-33] Hence, the YES programme is a distinct, co-designed and theoretically modelled intervention, with novel components. A

For peer review only - http://bmjopen.bmj.com/site/about/guidelines.xhtml

pilot evaluation of three early implementations of the YES programme that included 26 participants (mean age 18±1.7 years) showed it to be impactful on glycaemic control, with 46% (n=12) of participants achieving a clinically significant reduction in their HbA1c (≥5.5mmol/mol or 0.5% DCCT) at the 12-month follow-up. Programme uptake was 34% (n=26) with 96% (n=25) programme completion, which is similar to other programmes targeting young people.[25] Qualitative findings suggested that the programme had a positive psychosocial impact resulting in increased diabetes engagement and activation. The following active ingredients of the programme were also identified: social learning, peer support, relational care/support, and experiential learning. The youth worker was also highlighted by participants as being important in engaging them with the programme.

In the study outlined in this protocol, we propose to undertake a feasibility trial of the YES programme, with an integrated process evaluation. We have modelled the active ingredients of the programme to target outcomes related to psychosocial well-being, diabetes self-management activation and glycaemic control. In the process evaluation we will examine methodological, procedural and clinical uncertainties around the YES programme,[30-32] including: (1) estimates of likely recruitment and retention rates; (2) feasibility and acceptability of data collection instruments and data collection procedures; and (3) feasibility and acceptability of the programme. In line with standard feasibility study objectives,[31] we will not be testing the effect of the programme on outcomes, although we will estimate the impact of the programme on outcomes, to: consider the feasibility of progression to a definitive trial; and to inform a sample size calculation for such a trial. There will also be a mixed method element to the process evaluation,[32] to explore: (1) intervention acceptability; (2) the intervention experience and its impact on physical, mental and social well-being; (3) and parents' perspectives on the programme. These findings will be integrated to formulate potential mechanisms of change following programme exposure.

Aims and objectives

The aims of study are to: test the feasibility of the YES programme in terms of programme acceptance, implementability, recruitment and completion; and to estimate its efficacy in relation to clinical, psychosocial, and health care outcomes. The study objectives are to:

For peer review only - http://bmjopen.bmj.com/site/about/guidelines.xhtml

- Evaluate and optimise the research process: recruitment, willingness to be randomised, consent procedures, and data collection.
- Evaluate our ability to assess the programme's impact on self-management, patient activation, and clinical and psychosocial outcomes (optimising outcome selection and estimating the level effect).
- Establish the reach, fidelity, utility, feasibility, appropriateness, unintended consequences and sustainability of the YES intervention.
- Identify and explore the experiences of programme participants and those delivering the intervention.
- Optimise the intervention components, procedures and outcome measures.
- Obtain estimates of outcomes to inform the sample size calculation for a larger clinical evaluation study.

METHODS AND ANALYSIS

Study Design

The study has been designed as a feasibility waiting-list randomised controlled trial (RCT) with an integrated process evaluation. Participants will be randomised to YES exposure (phase 1) or a waiting list control and followed up for 6 months. At the 6 month follow up point, control participants will then be exposed to YES, in a phase 2 exposure. Both groups will be subsequently followed up for a further 6 months. This will ensure that all eligible young people will have the opportunity to participate in the programme while maintaining a controlled structure to YES exposure that will allow us to explore differences. Conducting an additional 6-month follow-up after phase 1, will also enable us to assess how sustainable the intervention is over a longer time period. The adoption of a waiting list design is important as it overcomes the ethical dilemma of denying control participants the opportunity to benefit from the programme. The study design is presented schematically in Figure 1.

(Figure 1 about here)

We have set the following progression criteria for a subsequent definitive trial: achieve minimum 30% recruitment of eligible participants; and ≥80% completion of final follow-up. We will also assess the impact of the intervention ingredients on target behaviours,

For peer review only - http://bmjopen.bmj.com/site/about/guidelines.xhtml

including activation of self-management, engagement in diabetes health care, and improved psychosocial functioning. These effects, alongside impact on glycaemic control, will be used to consider whether there is adequate potency in the programme and will determine whether a definitive trial will be conducted.

Setting

The study will be conducted in South London, United Kingdom, recruiting participants from the diabetes centres of five hospitals, providing care to the target population. These sites have been selected to ensure sociodemographic diversity, with a significant proportion of patients from lower socio-economic backgrounds and from Black Asian and Minority Ethnic (BAME) populations.

Participants and sample size

We aim to recruit 60 young people and randomise half to the phase 1 programme exposure, and half to the phase 2 exposure. The inclusion criteria will involve:

- Age 14-19 years;
- Diagnosis of T1DM;
- Sub-optimal glycaemic control (HbA1c >69 mmol/mol or 8.5%);[3]
- No current or planned attendance of other structured education programmes.

Participants will be excluded if they: have a severe physical or mental illness, unstable retinopathy, significant learning difficulties; are unable to communicate in English; or are pregnant. Participants meeting the inclusion criteria will be recruited opportunistically at the participating centres through clinical referrals and patient database searches. Based on recruitment to the pilot study we estimate that we will be able to recruit >30% of eligible patients. To increase engagement, we will run the full study, including programme occurrences and follow-up sessions, during school holidays as this was identified by young people as the optimal period for running the YES programme.

As a feasibility evaluation, the study is not statistically powered to estimate a clinical effect. Instead, we aim to estimate the effect-size/standard deviation of outcomes to formulate a power calculation for a definitive trial. National Institute for Health Research (NIHR) guidance

For peer review only - http://bmjopen.bmj.com/site/about/guidelines.xhtml

indicates that samples of 24-50 are adequate for this purpose.[33-35] We have inflated our sample to allow for an estimated 20% drop-out rate.

Intervention and control conditions

The intervention is the YES programme as previously described. The programme exposure is sequenced as follows: (1) initial contact from the youth worker introducing the YES intervention; (2) attendance at three all-day YES sessions in non-healthcare settings (groups of 10); (3) post-programme networking through social media and (4) follow-up events facilitated by the youth worker. Sessions will be led by a peer-educator and a health professional and delivered face-to-face. Participants in the waiting-list control arm of the study will receive usual care following initial randomisation and will be exposed to the YES intervention at 6 months (phase 2 exposure). Usual care in this patient population typically involves outpatient consultations. To minimise potential external confounding factors, we will monitor any other educational interactions that participants might experience during the follow-up period in both groups.

Randomisation

Participants will be randomised to either the phase 1 YES exposure or to the waiting list control (for phase 2 exposure), using computer generated randomisation in blocks of 20. As the intervention is open blinding is not possible. The waiting list design will help to maintain young people's motivation to participate in the phase 1 and 2 allocations. As part of our feasibility work, we will consider how acceptable randomisation is to young people with diabetes. Young people's lives are quite dynamic in this period with frequent changes such as moving schools starting further or higher education, or employment. Hence, while we will explain why randomisation is helpful to us, but if young people decline randomisation they will be offered a preference for when they join the programme.

Outcome measures

As a feasibility study, the objective is to optimise outcome selection and determine outcome effects prior to a definitive RCT. The primary outcome to assess the potential clinical effect of the programme will be HbA1c and we will examine clinically significant reductions of 5.5mmol/mol (0.5% DCCT) and mean differences between groups over time. While HbA1c is

For peer review only - http://bmjopen.bmj.com/site/about/guidelines.xhtml

a proxy measure of complication risk, it is the most commonly adopted measure for determining the short to medium term impact of an intervention and is the recommended measure from the National Institute for Health and Care Excellence (NICE) for assessing the clinical and health economic benefits of diabetes interventions. In addition, our preliminary work and logic model show that the programme can mediate health behaviours by impacting on the psychosocial factors that can mediate self-management activation leading to improved glucose control. Hence, we will also assess the psychosocial and behavioural impacts of the programme, using the following measures:

- Self-management activation (frequency of blood glucose monitoring, insulin adherence);
- Self-Management of Diabetes in Adolescence Scale (SMOD-A);[36]
- Confidence in managing own health care (Confidence in Diabetes Self-Care Scale (CDSS);[37]
- Diabetes Quality of Life Instrument (DQOL)-adapted for youth;[38]
- Illness perception (Brief Illness Perception Questionnaire, IPQ-B);[39]

Our pilot work also showed a potential reduction in diabetes related emergency care events (ambulance call-outs, Accident and Emergency attendance or hospitalisations), hypoglycaemia (blood glucose <3.5 mmol/L) and severe hypoglycaemic events (requiring third party assistance). Therefore, we will also measure the incidents of these events prior to and following YES exposure. The participants weight will be motored, as weight gain can occur as glucose levels improve.

Study procedure

Potential participants will be recruited from the participating diabetes centres. Since it can be challenging to engage young people with T1DM, especially those from deprived and ethnically diverse groups, a youth worker role will be incorporated into the recruitment process to identify potential participants and support them in considering participation in the study. Young people will then be screened for eligibility and consented. Parental consent will also be gained from those aged <16 years of age. We will ask eligible young people who decline participation to give a reason for their decision. The participants will then be randomly allocated as previously described.

For peer review only - http://bmjopen.bmj.com/site/about/guidelines.xhtml

Data collection

Data (questionnaires, body weight and medical-record data) will be collected at baseline, then again at 6 and 12 months. Baseline data will also include: socio-demographics; education/employment; and diabetes self-management behaviours including insulin use and blood glucose testing. Patient records will be examined to collect data on: HbA1c; hospitalisations and emergency care; diabetes education attendance; screening attendance; and attendance at appointments. For baseline this will be all events in the preceding 12 months, and post exposure will be for the 12 months following exposure (or 6 months' post exposure for those exposed at phase 2).

Data analysis

The data will be analysed to determine the proportion of patients achieving the progression criteria; and the target reduction in HbA1c from baseline to follow-up (6 and 12 months), which will be estimated in both intervention (YES) and control groups. We will also estimate the standard deviation (SD) of the mean difference between the intervention and control group in HbA1c at 6 months; for the control group the SD of the mean difference between HbA1c at 12 months and pre-exposure (Phase 2) and for the phase 1 intervention group the SD of the mean HbA1c difference between post (6 and 12 months) and pre-exposure. These data will help inform the sample size calculation for a definitive trial.

Process evaluation

The process evaluation has been designed in accordance with the MRC process evaluation model for complex interventions,[32] aiming to explore the setting, implementation, and mechanisms of action of the programme to enable interpretation of the observed outcomes. Our process evaluation will also use an established taxonomy of outcomes to be evaluated for understanding implementation.[40-41] Following this taxonomy, we will assess the reach, fidelity; its perceived acceptability, appropriateness and feasibility; unintended consequences; and potential sustainability considerations. In addition, we will elicit potential mechanisms of action, including changes in behaviours, and how they relate to programme delivery and outcomes, using the COM-B (capability, opportunity, motivation to perform behaviour) and Behaviour Change Wheel (BCW) frameworks.[42-43] Further, we will apply the

For peer review only - http://bmjopen.bmj.com/site/about/guidelines.xhtml

Consolidated Framework for Implementation Research (CFIR) to study how the programme can be optimally embedded within routine diabetes care (i.e. to capture systematically implementation and adoption drivers/barriers); and to optimise the design of a subsequent trial.[44]

The process evaluation will involve a mixed-method approach, including use of validated brief implementation outcome surveys[45] (to be delivered to both young people with diabetes and health providers involved in YES) and one-to-one interviews with young people, their relatives and healthcare professionals. The data collection tools and analytic models have been designed with reference to the BCW framework[43] and to provide the data for CFIR[44] analysis considering the barriers and facilitators to the intervention. To ensure relevance and feasibility, implementation data collection tools will be refined with the study patient and public representative groups. Semi-structured interviews exploring the implementation outcomes and programme impact within the first 3 months of the completion of the programme will be conducted with a random sub-sample of participants (n=12 from each arm of the study, 4 in each centre), including participating young peoples' relatives (n=10); and the healthcare professionals involved in the delivery of the programme (n=6). All interviews will be digitally recorded and transcribed verbatim in preparation for analysis using thematic analysis,[46] mapping the data using the BCW[43] and CFIR[44] frameworks.

To assess perception of how appropriate, acceptable and feasible the programme is to young people and healthcare professionals, we will use previously validated short pragmatic surveys.[45] This will allow quantitative assessment against the study end-points. We will also undertake:

- An audit of the number of eligible young people that decline participation, considering their clinical and sociodemographic profiles;
- An audit of intervention fidelity using an adherence checklist based on the YES protocol;
- Exit questionnaires with attending young people to rate programme satisfaction and utility; and to identify 3 strengths, weaknesses and areas for improvement.

For peer review only - http://bmjopen.bmj.com/site/about/guidelines.xhtml

The audit and questionnaire data will be analysed to examine the utilisation and satisfaction with the programme. Intervention fidelity will be assessed from the audit data detailing the delivery of the different intervention components. We will also monitor control conditions in the first 6 months to consider whether any extraneous interventions or clinical changes occurred that might impact on the observed outcomes. While the study will be recruiting participants from five diabetes centres, we do not anticipate significant site level effects on outcomes as the intervention will be delivered by the same clinicians and peer educators throughout, following the YES curriculum. However, we will monitor any variations in the general care for each centre, or any changes in the care provided to the target population during the follow-up period.

Health economic evaluation

While a full health economic analysis is beyond the scale of this feasibility study, we will collect data as part of the process evaluation to inform health economic analysis for a future definitive trial. The analysis will have 3 elements:

1. A full economic costing of the programme delivery costs.
2. An audit of medical records to consider health care utilisation and in particular impact on high-cost emergency care episodes.
3. Modelling cost benefits by considering the risk of long-term complications based on impact on glycaemic control using established health economic assumptions.[47]

Review and optimisation event

In the final phase of the study, we will organise a YES review event with a purposively selected group (age and gender) of participants (n=16), their family members or partners, health professionals from participating services and the research team. The aim of the event will be to share study findings and collate feedback about the programme through facilitated round table discussions, in which participants will be encouraged to offer ideas to further improve the YES programme. Following this event, and based on an integrated assessment of the data collected, the intervention protocol and outcome measures will be revised for use in a definitive trial.

For peer review only - http://bmjopen.bmj.com/site/about/guidelines.xhtml

Patient and public involvement

A patient and public involvement (PPI) group consisting of young people who had previously attended the YES programme contributed to the development, design and conduct of the study (01/2018 to date), including: outcome measure selection; topic guide content for interviews; and formulating the information sheets and consent forms for ethics. In terms of outcome measures, the group advised us that only 3-4 questionnaires should be used as any more would be too burdensome and that this would affect quality of responses received. We showed the group 9 questionnaires which we related to the target psychosocial constructs and behaviours. They felt that many of the measures were too personal, confusing or distressing. Hence, we removed measures for: diabetes distress; diabetes stigma; and depression. We may, however, pick-up these themes in the interviews. We have recently expanded the PPI group to include some young people who have not attended the YES programme; and we have set up an additional group of parents of young people with T1DM. These groups will support the research team in the analysis, write-up and dissemination of study findings; and help in the process of optimising the programme and study procedures for a future trial.

PPI has also been important in helping prepare the study in relation to Covid-19 restrictions. Prior to starting the study, we conducted a survey of 74 young people with diabetes to explore whether they would be willing to meet in a group once the restrictions had lifted. Over half of the respondents (n=38) identified they were likely or very likely to attend a face-to-face education group or activity for young people with diabetes if it were offered to them. Three-quarters of the respondents (n=56) said that their views on attending YES had not been impacted by the pandemic.

DISCUSSION

In this paper, we have described the protocol for a feasibility study to assess a complex psychoeducational intervention for young people with T1DM. As we have highlighted, there is a need to develop and test new educational approaches for young people with T1DM, which take account of the significant psychological and social challenges they experience in this period of their lives. The YES programme was co-designed with young people and has been

For peer review only - http://bmjopen.bmj.com/site/about/guidelines.xhtml

theoretically modelled to target the challenges relevant to them, in order that they can adapt to living with diabetes. The study we have outlined here will help us establish whether the active mechanisms within the programme connect with how young people engage with their diabetes self-management as a condition for improving their glycaemic control, thereby reducing the risk of future complications. The study will also assess both quantitatively and qualitatively how the programme effects young people's orientation to their diabetes (positive or negative) psychologically and socially.

In conducting a trial of a clinical intervention, such as YES, it is important to consider how the intervention is experienced by those receiving and delivering it. Following the MRC model for complex interventions,[32] the process evaluation will assess the feasibility of the YES programme in terms of acceptance, implementability, recruitment and completion. Application of the COM-B[42] and BCW[43] frameworks will allow us to elicit/confirm the potential mechanisms of action and how they relate to programme delivery. This analysis will also help us optimise the intervention components and outcome measures. Further, use of the CFIR framework to delineate barriers and drivers to YES implementation and Proctor et al's[40] taxonomy of implementation outcomes will allow systematic study of implementation aspects of YES, with both young people, their parents and T1DM care providers. These aspects of the study will be important in understanding how the intervention works in the context of its delivery and how it might be enhanced.[40] This evidence will be needed for the design of a subsequent definite trial of YES: based on the data that we collect on YES delivery, and if the implementation data collection of the current study, including both scales and qualitative data, is successful, it follows that a definite trial of YES may take a 'hybrid' format. Hybrid effectiveness-implementation studies include both clinical and implementation outcomes and evaluate both clinical effectiveness and implementation success of different strategies to deliver YES.[48] Such designs are efficient, in that they offer both clinical evidence and, simultaneously, evidence on what implementation strategies and support needs to be in place for YES to be scaled successfully outside the trial centres. The current study will provide the early information required for such a hybrid design to be applied.

Given the inherent complexity of the target population for the YES programme, running a clinical trial in this population will be challenging. Hence, the feasibility questions posed by

For peer review only - http://bmjopen.bmj.com/site/about/guidelines.xhtml

the study in respect of recruitment, retention and data completeness will be important. We also hope that the qualitative work within the process evaluation will also allow us to learn how the research approach can be optimised prior to a full trial. A key consideration in relation to this will be the study design, particularly whether randomisation and the use of a waiting list model will be feasible. There is a growing recognition that conducting clinical trials in adolescents and young adults is challenging, with the suggestion that alternative more adaptive research models are needed.[49] While we have sort to address these challenges with the waiting list design and including the option for preference should participants decline randomisation; such adaptations can introduce bias in estimating observations.[50] While we cannot fully assess these biases in this study it will be important to consider whether, for example, those who decline randomisation and express a preference for either phase 1 or phase 2 exposure show stronger of weaker effects compared to those randomised. Following completion of the study, we will use the YES review event and our PPI groups to help us consider how best to design a definitive study should we meet our progression criteria.

ETHICS AND DISSEMINATION

The study described in this protocol has been reviewed and given favourable opinion by the London – Camden and Kings Cross Research Ethics Committee (REC number: 21/LO/0231; IRAS number: 279877) on April 20th 2021. Informed consent will be sought from all research participants for this study. The study will be conducted in accordance with Good Clinical Practice and recommendations for physicians involved in research on human subjects adopted by the 18th World Medical Assembly, Helsinki 1964 and later revisions. The study will produce key information to the stakeholders on the planning, funding and implementation of the interventions under investigation. Hence, the findings will be disseminated through peer-reviewed journals, relevant national and international conferences, as well as educational events at individual hospitals to ensure that they are brought to the appropriate stakeholders. We will also disseminate the findings to young people with diabetes and their families by sharing a lay summary of the findings on the Diabetes UK website and via relevant social media groups.

For peer review only - http://bmjopen.bmj.com/site/about/guidelines.xhtml

DATA SHARING STATEMENT

We will make anonymised data from the study available following a justified request for access.

FUNDING

The funding for this study was supported by the *Diabetes UK* (reference 19/0006055). TS is also supported by the Welcome Trust (219425/Z/19/Z). NS is funded by the *National Institute for Health Research* (NIHR) via the '*Applied Research Collaboration South London*' (ARC South London; grant number not available) at King's College Hospital National Health Service (NHS) Foundation Trust, London, UK. NS is a member of King's Improvement Science, which offers co-funding to the NIHR ARC South London and is funded by King's Health Partners (Guy's and St Thomas' NHS Foundation Trust, King's College Hospital NHS Foundation Trust, King's College London and South London and Maudsley NHS Foundation Trust), and the Guy's and St Thomas' Foundation. The funding agreements ensured the authors' independence in designing the study, interpreting the data, writing, and publishing the report. The views expressed are those of the authors and not necessarily those of the NHS, the NIHR or the Department of Health and Social Care.

ACKNOWLEDGEMENTS

We would like to thank the representatives of the young people with T1DM, as well as the health care professionals involved, for their time and commitment in the design of this study protocol.

COMPETING INTERESTS

NS is the Director of London Safety & Training Solution ltd, which offers training in patient safety, implementation solutions and human factors to healthcare organisations and the pharmaceutical industry. TS received consultancy fees from Roche Diagnostics and Cancer Alliances.

For peer review only - http://bmjopen.bmj.com/site/about/guidelines.xhtml

AUTHORS' CONTRIBUTIONS

As per International Committee of Medical Journal Editors guidelines, all co-authors have contributed as following:

- made substantial contributions to conception and design, or acquisition of data, or analysis and interpretation of data;
- been involved in drafting the manuscript or revising it critically for important intellectual content;
- given final approval of the version to be published. Each author should have participated sufficiently in the work to take public responsibility for appropriate portions of the content;
- agreed to be accountable for all aspects of the work in ensuring that questions related to the accuracy or integrity of any part of the work are appropriately investigated and resolved.

For peer review only

For peer review only - http://bmjopen.bmj.com/site/about/guidelines.xhtml

LIST OF ABBREVIATIONS

BCW = Behaviour Change Wheel

CDSS = Confidence in Diabetes Self-Care Scale

COM-B = Capability, Opportunity, Motivation to Perform Behaviour

DKA = Diabetic-Keto-Acidosis

DQOL = Diabetes Quality of Life Instrument

HbA1c = Glycated Haemoglobin

IPQ-B = Illness Perception (Brief Illness Perception Questionnaire

MRC = Medical Research Council

NICE = National Institute for Health and Care Excellence

NIHR = National Institute for Health Research

NPT = Normalisation Process Theory

PPI = Patient and Public Involvement

RCT = Randomised Controlled Trial

SMOD-A = Self-Management of Diabetes in Adolescence Scale

T1DM = Type 1 Diabetes Mellitus

YES = Youth Empowerment Skills

For peer review only

For peer review only - http://bmjopen.bmj.com/site/about/guidelines.xhtml

REFERENCES

1. Hamilton J, Daneman D. Deteriorating diabetes control during adolescence: physiological or psychological? Journal of Pediatr Endocrinol Metab 2002;15(2):115-126.
2. Northam EA, Lin A, Finch S, Werther GA, Cameron FJ. Psychosocial well-being and functional outcomes in youth with Type 1 diabetes. Diabetes Care 2010;33(7):1430–1437.
3. National Paediatric Diabetes Audit. National Paediatric Diabetes Audit Report 2018-19. London: Royal College of Paediatrics and Child Health; 2019. Available: final_npda_core_report_2018-2019.pdf (rcpch.ac.uk)
4. White M, O'Connell MA, Cameron FJ. Transition to adult endocrine services: the diabetes perspective. Best Pract Res Clin Endocrinol Metab 2015;29(3): 497–504.
5. Gibb FW, Teoh WL, Graham J, Lockman KA. Risk of death following admission to a UK hospital with diabetic ketoacidosis. Diabetologia 2016;59(10):2082–2087.
6. Monaghan M, Helgeson V, Wiebe D. Type 1 diabetes in young adulthood. Curr Diabetes Rev 2015;11(4):239–250.
7. Oris L, Rassart J, Prikken S, Verschueren M, Goubert L Moons P, Berg CA, Weets I, Luyckx K. Illness identity in adolescents and emerging adults with Type 1 diabetes: introducing the Illness Identity Questionnaire. Diabetes Care 2016;39(5):757-763.
8. Luyckx K, Seiffge-Krenke I, Schwartz SJ, Goossens L, Weets I, Hendrieckx C, Groven C. Identity development, coping, and adjustment in emerging adults with a chronic illness: the sample case of Type 1 diabetes. Journal of Adolescent Health 2008;43(5):451-458.
9. Palladino DK, Helgeson VS, Reynolds KA, Becker DJ, Siminerio LM, Escobar O. Emerging adults with type 1 diabetes: a comparison to peers without diabetes. J Pediatr Psychol 2003;38(5):506–517.
10. Herzer M, Hood,K. Anxiety symptoms in adolescents with Type 1 diabetes: association with blood glucose monitoring and glycemic control. J Pediatr Psychol 2010;35(4):415–425.
11. Eating problems in adolescents with Type 1 diabetes: a systematic review with meta-analysis.Young V, Eiser C, Johnson B, Brierley S, Epton T, Elliott J, Heller S Diabet Med. 2013 Feb; 30(2):189-98.
12. Rawshani A, Sattar N, Franzen S, Rawshani A, Hattersley AT, Svensson AM, ELiassion B, Gudbjornsdottir S. Excess mortality and cardiovascular disease in young adults with Type 1

For peer review only - http://bmjopen.bmj.com/site/about/guidelines.xhtml

diabetes in relation to age at onset: a nationwide, register-based cohort study. Lancet 2018; 392:477–486.

13. Hains AA, Berlin KS, Davies WH, Parton EA, Alemzadeh R. Attributions of adolescents with type 1 diabetes in social situations: relationship with expected adherence, diabetes stress, and metabolic control. Diabetes Care 2006;29(4):818–822.

14. Whittemore R, Jaser S, Chao A, Grey M. Psychological experience of parents of children with Type 1 diabetes: a systematic mixed-studies review. Diabetes Educ 2012;38(4):562–579.

15. Eilander MMA, Snoek FJ, Rotteveel J, Aanstoot HJ, Bakker-van Waarde WM, Houdijk ECAM, Nuboer R, Winterdijk P, deWit M. Parental diabetes behaviors and distress are related to glycemic control in youth with Type 1 diabetes: longitudinal data from the DINO Study. J Diabetes Res 2017. Available: https://doi.org/ 10.1155/2017/1462064

16. Whittemore R, Liberti L, Jeon S, Chao A, Jaser S, Grey M. Self-management as a mediator of family functioning and depressive symptoms with health outcomes in youth with type 1 diabetes. West J Nurs Res 2014;36(9):1254–1271.

17. Khanolkar AR, Amin R, Taylor-Robinson D, Viner RM, Warner JT, Stephenson T. Young people with Type 1 diabetes of non-white ethnicity and lower socio-economic status have poorer glycaemic control in England and Wales. Diabet Med 2016;33(11):1508–1515.

18. Elders V, Keen A, Gold A. Adults with type 1 diabetes: what factors are associated with disengagement from health services? Practical Diabetes 2013;31(3):117-118a.

19. Snow R, Fulop N. Understanding issues associated with attending a young adult diabetes clinic: a case study. Diabet Med 2012;29(2):257-259.

20. Peters A, Lafell L, the American Diabetes Association Transitions Working Group. Diabetes care for emerging adults: recommendations for transition from pediatric to adult diabetes care systems: a position statement of the American Diabetes Association Diabetes Care. 2011;34(11):2477-2485.

21. Sheehan AM, While AE, Coyne I. The experiences and impact of transition from child to adult healthcare services for young people with type 1 diabetes: a systematic review. Diabet Med 2015;32(4):440-458.

22. Pyatak EA, Sequeira PA, Whittemore R, Vigen CP, Peters AL, Weigensberg MJ. Disrupted transition from paediatric to adult diabetes care in young adults with Type 1 diabetes. Diabet Med 2014;31(12):1615–1624.

For peer review only - http://bmjopen.bmj.com/site/about/guidelines.xhtml

23. National Institute for Health and Care Excellence (NICE). Type 1 diabetes in adults: diagnosis and management [NG17] London. Available: www.nice.org.uk/guidance/ng17.
24. National Institute for Health and Care Excellence (NICE). Diabetes (Type 1 and Type 2) in children and young people: diagnosis and management [NG18]. Available: https://www.nice.org.uk/guidance/ng18
25. Coates VE, Horigan G, Davies M, Davies MT. Exploring why young people with Type 1 diabetes decline structured education with a view to overcoming barriers. Diabet Med 2017;34(8):1092-1099.
26. Coates V, Chaney D, Bunting B, Shorter GW, Shevlin M. Evaluation of the Effectiveness of a Structured Diabetes Education Programme (CHOICE) on Clinical Outcomes for Adolescents with Type 1 Diabetes: A Randomised Controlled Trial. J Diabetes Metab 2013;4:280.
27. Bandura A. Social Learning Theory. Engelwood Cliffs, N.J.: Prentice Hall; 1977.
28. Leventhal H, Nerenz DR, Steele DJ. Illness Representations and Coping with Health Threats. In: Baum, A, Taylor SE, Singer JE. Eds., Handbook of Psychology and Health Volume IV: Social Psychological Aspects of Health. Hillsdale, NJ: Erlbaum; 1984.p.219-252.
29. Chaiken S, Wood W, Eagly AH. Principles of persuasion. In: Higgins ET, Kruglanskşi AW. Eds., Social Psychology: Handbook of Basic Principles. New York, Guilford Press; 1996.
30. Skivington K, Matthews L, Simpson SA, et al. Framework for the development and evaluation of complex interventions: gap analysis, workshop and consultation-informed update. Health Technol Assess. 2021;25(57):1-132. doi:10.3310/hta25570
31. Eldridge SM, Lancaster GA, Campbell MJ, et al. Defining feasibility and pilot studies in preparation for randomised controlled trials: development of a conceptual framework. PLoSOne 2016;11:e0150205.doi:10.1371/journal.pone.0150205
32. Moore GF, Audrey S, Barker M, Bond L, Bonell C, Hardeman W, Moore L, O'Cathain A, Tinati T, Wight D, Daird J. Process evaluation of complex interventions: Medical Research Council guidance. BMJ (Clinical research ed.) 2015;350:h1258.
33. Sim J, Lewis M. The size of a pilot study for a clinical trial-considerations of precision and efficiency. J Clin Epidemiol 2012;65(3):301-308.
34. Hooper R. Justifying sample size for a feasibility study [Internet] London: National Institute for Health Research; 2019. Available from: https://www.rds-london.nihr.ac.uk/wpcms/wp-

For peer review only - http://bmjopen.bmj.com/site/about/guidelines.xhtml

content/uploads/2019/02/Justifying-sample-size-for-feasibility-study-updated-22-Feb-2019.pdf.

35. Julious SA. Sample size of 12 per group rule of thumb for a pilot study. Pharmaceut Statist 2005;4:287-291.

36. Schilling LS, Dixon JK, Knafl KA, Lynn MR, Murphy K, Dumser S, Grey M. A new self-report measure of self-management of type 1 diabetes for adolescents. Nurs Res 2009;58(4):228-236.

37. Van Der Ven NC, Weinger K, Yi J, Pouwer F, Ader H, Van Der Ploeg HM, Snoek FH. The confidence in diabetes self-care scale: psychometric properties of a new measure of diabetes-specific self-efficacy in Dutch and US patients with Type 1 diabetes. Diabetes Care 2003;26(3):713-718.

38. Ingersoll GM, Marrero DG. A modified quality-of-life measure for youths: psychometric properties. Diabetes Educ 1991;17(2):114–120.

39. Broadbent E, Petrie KJ, Main J, Weinman J. The brief illness perception questionnaire. J Psychosom Res 2006;60(6):631–637.

40. Proctor E, Silmere H, Raghavan R, Hovmand P, Aarons G, Bunger A, Griffey R, Hensley M. Outcomes for implementation research: Conceptual distinctions, measurement challenges, and research agenda. Adm Policy Ment Health 2011;38(2):65-76.

41. Powell BJ, Waltz TJ, Chinman MJ, Damschroder LJ, Smith JL, Matthieu MM, Proctor EK, Kirchner JE. A refined compilation of implementation strategies: results from the Expert Recommendations for Implementing Change (ERIC) project. Implementation Sci 2015;10:21.

42. Barker F, Atkins L, de Lusignan S. Applying the COM-B behaviour model and behaviour change wheel to develop an intervention to improve hearing-aid use in adult auditory rehabilitation. Int J Audiol 2016;55(sup3):S90-S98.

43. Michie S, Atkins L, West R. The Behaviour Change Wheel: a guide to designing interventions. Silverback Publishing; 2014.

44. Kirk MA, Kelley C, Yankey N, Birken SA, Abadie B, Damschroder L. A systematic review of the use of the Consolidated Framework for Implementation Research. Implement Sci 2016;11:72.

For peer review only

For peer review only - http://bmjopen.bmj.com/site/about/guidelines.xhtml

45. Weiner BJ, Lewis CC, Stanick C, Powell BJ, Dorsey CN, Clary AS, Boynton MH, Halko H. Psychometric assessment of three newly developed implementation outcome measures. Implementation Sci 2017;12:108.

46. Ritchie J, Lewis J, Nicholls CM, Ormston R. Qualitative Research Practice: A Guide for Social Science Students and Researchers: London: Sage.

47. National Institute for Health and Care Excellence (NICE). Diabetes (Type 1 and Type 2) in children and young people: diagnosis and management [NG18]. Section 20 Health economics. Available: https://www.ncbi.nlm.nih.gov/books/NBK315806/.

48. Kemp CG, Wagenaar BH, Haroz EE. Expanding hybrid studies for implementation research: Intervention, implementation strategy, and context. Front Public Health 2019;7:325.

49. Baiardi, P., Giaquinto, C., Girotto, S. et al. Innovative study design for paediatric clinical trials. Eur J Clin Pharmacol 67, 109–115 (2011). https://doi.org/10.1007/s00228-011-0990-y

50. Cunningham JA, Kypri K, McCambridge J. Exploratory randomized controlled trial evaluating the impact of a waiting list control design. BMC Med Res Methodol. 2013;13(1):150. doi:10.1186/1471-2288-13-150

For peer review only - http://bmjopen.bmj.com/site/about/guidelines.xhtml

FIGURE CAPTIONS

Figure 1. Consort diagram for the Youth Empowerment Skills (YES) Programme feasibility study

For peer review only

For peer review only - http://bmjopen.bmj.com/site/about/guidelines.xhtml

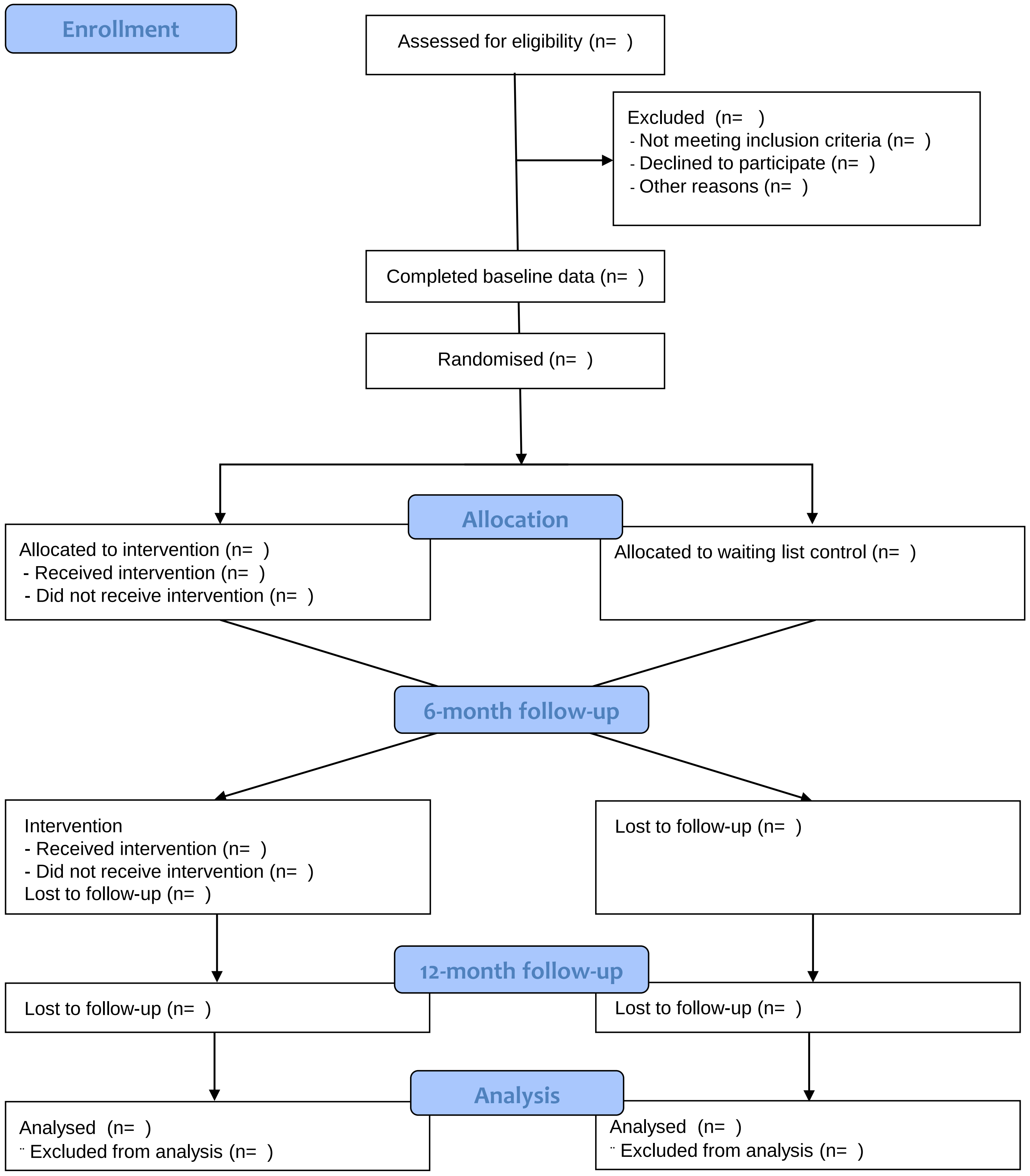
Enrollment
Assessed for eligibility (n=)
Excluded (n=)
- Not meeting inclusion criteria (n=)
- Declined to participate (n=)
- Other reasons (n=)
Completed baseline data (n=)
Randomised (n=)
Allocation
Allocated to intervention (n=)
- Received intervention (n=)
- Did not receive intervention (n=)
Allocated to waiting list control (n=)
6-month follow-up
Intervention
- Received intervention (n=)
- Did not receive intervention (n=)
Lost to follow-up (n=)
Lost to follow-up (n=)
12-month follow-up
Lost to follow-up (n=)
Lost to follow-up (n=)
Analysis
Analysed (n=)
¨ Excluded from analysis (n=)
Analysed (n=)
¨ Excluded from analysis (n=)

Protocol for A Feasibility Study and Process Evaluation of a Psychosocially Modelled Diabetes Education Programme for Young People with Type 1 Diabetes: the Youth Empowerment Skills (YES) Programme

Journal:	*BMJ Open*
Manuscript ID	bmjopen-2022-062971.R1
Article Type:	Protocol
Date Submitted by the Author:	05-May-2022

Complete List of Authors:	Kariyawasam, Dulmini; Guy's and Saint Thomas' Hospitals NHS Trust, Diabetes<br>Soukup, Tayana ; King's College London, Health Service and Population Research Department; King's College London<br>Parsons, Judith; King's College London Florence Nightingale School of Nursing and Midwifery, Adult Nursing<br>Sevdalis, Nick; King's College London<br>Baldellou Lopez, Maria ; King's College London<br>Forde, Rita; King's College London, Faculty of Nursing, Midwifery and Palliative Care<br>Ismail, Khalida; King's College London<br>Jones, Marie; Guy's Hospital<br>Ford-Adams, Martha; King's College Hospital NHS Foundation Trust, Diabetes Department<br>Yemane, Nardos; Guy's and St Thomas' NHS Foundation Trust, Diabetes Department<br>Pender, Siobhan; Guy's and Saint Thomas' NHS Foundation Trust Neurology Department<br>Thomas, Stephen; Guy's and St Thomas' Hospitals NHS Trust, Diabetes Department<br>Murrells, Trevor; King's College London, National Nursing Research Unit<br>Silverstien, Alex; King's College London<br>Forbes, Angus; King's College London, Division of Care in Long-term Conditions

**Primary Subject Heading**:	Diabetes and endocrinology
Secondary Subject Heading:	Diabetes and endocrinology, Evidence based practice, Paediatrics
Keywords:	DIABETES & ENDOCRINOLOGY, General diabetes < DIABETES & ENDOCRINOLOGY, General endocrinology < DIABETES & ENDOCRINOLOGY

For peer review only - http://bmjopen.bmj.com/site/about/guidelines.xhtml

Protocol for A Feasibility Study and Process Evaluation of a Psychosocially Modelled Diabetes Education Programme for Young People with Type 1 Diabetes: the Youth Empowerment Skills (YES) Programme

Dulmini Kariyawasam,[1] Tayana Soukup,[2-5*] Judith Parsons,[6] Nick Sevdalis,[2] Maria Baldellou Lopez,[2] Rita Forde,[6] Khalida Ismail,[7] Marie Jones,[1] Martha Ford-Adams,[8] Nardos Yemane,[1] Siobhan Pender,[1] Stephen Thomas,[1] Trevor Murrells,[6] Alex Silverstien, Angus Forbes[6]

Author affiliations

[1] Diabetes Department, Guy's and St Thomas' NHS Foundation Trust, London, UK

[2] Centre for Implementation Science, Health Service and Population Research Department, Institute of Psychiatry, Psychology and Neuroscience, King's College London, UK

[3]Institute of Global Health Innovation, Faculty of Medicine, Imperial College London, UK

[4]School of Health & Sport Sciences, University of Suffolk, UK

[5]Department of Epidemiology and Environmental Health, The State University of New York at Buffalo, USA

[6] Division of Care in Long-term Conditions, King's College London, UK

[7] Department of Psychological Medicine, Institute of Psychiatry, King's College London, UK

[8] Diabetes Department, King's College Hospital, London, UK

Full address for correspondence

*Dr Tayana Soukup, Centre for Implementation Science, Health Service & Population Research Department, King's College London, 16 De Crespigny Park, London SE5 8AF. Email: tayana.soukup@kcl.ac.uk Telephone Number: +44207 848 0272

Word count: 4538/4000

For peer review only - http://bmjopen.bmj.com/site/about/guidelines.xhtml

ABSTRACT (268/300w)

Introduction. Adolescence is a challenging period for young people with type 1 diabetes, associated with worsening glycaemia and care disengagement. Educational interventions in this period tend to focus on diabetes-specific skills, with less emphasis on the psychosocial challenges associated with diabetes experienced by young people. To address this limitation, we co-designed with young people a psychosocially modelled programme of diabetes education, named 'Youth Empowerment Skills' (YES). The programme aims to facilitate a positive adaptation to life with diabetes and engagement with diabetes care through peer-based learning, immersive simulations, and support from an outreach youth worker. Here we present a protocol for a feasibility study of the YES programme.

Methods and analysis. The study was designed following the Medical Research Council Complex Intervention Evaluation Framework to: test the feasibility (acceptance, implementability, recruitment and completion) of the YES programme; and estimate its efficacy in relation to metabolic and psychosocial outcomes. The study will take place in diabetes centres serving socio-culturally diverse populations. We will conduct a feasibility RCT (waiting-list design) with integrated process evaluation. Fifty young people with type 1 diabetes (aged 14-19 years) will be randomly allocated to either the YES intervention or a waiting-list control. Randomisation acceptability will be assessed with provision for a preference allocation. Outcomes will be evaluated at 6 months, at which point the waiting list participants will be exposed to the YES programme with further follow-up to 12 months. A simultaneous process evaluation will use a mixed-methods approach collecting qualitative and quantitative data. Study findings will be used to optimise the intervention components, outcome measures and recruitment methods to inform a subsequent definitive trial.

Ethics and dissemination. The protocol has ethical approval from the UK Health Research Authority (approval IRAS project ID:279877). Findings will be disseminated in multiple formats for lay and professional audiences.

Key words: Type 1 diabetes, young people, psychoeducational programme

Trial registration number: Clinicaltrials.gov identifier NCT04670198

Protocol date and version: 07 April 2021, v1.1

For peer review only - http://bmjopen.bmj.com/site/about/guidelines.xhtml

Strengths and limitation of the study

- The study will test a novel co-designed psychoeducational intervention for young people with Type 1 diabetes designed to help promote a positive emotional and social adaptation to a life with diabetes.
- An integrated process evaluation within the study will develop our understanding of how to enhance study procedures and gain insights into programme implementation for future studies.
- The randomised waiting-list design allows for the comparison of the intervention and usual care exposures without the ethical implications of not providing the intervention to the control group.
- The target population for this study can be resistant to healthcare interventions and have significant school and social commitments, hence the study will consider recruitment feasibility and the acceptability of randomisation versus preference for group allocation.

For peer review only

For peer review only - http://bmjopen.bmj.com/site/about/guidelines.xhtml

INTRODUCTION

Impact of T1DM on young people

Type 1 diabetes (T1DM) in adolescence and early adulthood can be a very challenging period in the lives of young people. During this period young people can begin to dissociate from their diabetes, leading to a decline in their glycaemic control and an increased risk of acute events such as recurrent diabetic-keto-acidosis (DKA).[1-5] Data from the most recent National Paediatric Diabetes audit in the United Kingdom showed that only 10% of young people aged 14-19 years achieved a glycated haemoglobin (HbA1c) within the current target range of ≤48mmol/mol(6.5%); and a fifth had a HbA1c >75mmol/mol(9%) which is associated with a high risk of diabetes complications.[3]

Furthermore, adolescence and early adulthood is also a period when young people are forming their personal and social identities, and diabetes can have a detrimental impact on their psychosocial development.[6,7] During this period, young people can develop negative emotional constructs about diabetes, as they see their diabetes as an impediment to social relationships and life opportunities.[8] Consequently, some young people may develop maladaptive coping strategies such as avoidance (omitting insulin and reducing their glucose monitoring), or psychological morbidities (fear, anxiety, depression and disordered eating behaviours).[9,10,11] Young people with diabetes can also feel stigmatised,[12] further driving negative coping strategies (e.g., not using insulin in public or running glucose levels high to avoid hypoglycaemia).

During this period young people are also developing their personal autonomy, which can lead to conflicts with parents who may find allowing the young person to take control over their diabetes self-management behaviours and health management challenging.[13] Relationships can become emotionally charged with conflict, excess vigilance and distress, difficulties that can exacerbate disengagement and are associated with poorer glycaemic control.[14,15] Negative interactions with diabetes health professionals have also been identified as contributing to young people disengaging from their care.[16]

For peer review only - http://bmjopen.bmj.com/site/about/guidelines.xhtml

Current care models for young people with T1DM

Developing effective diabetes care for young people with T1DM is challenging. The transition from child to adult care has long been identified as a period when young people can disengage from their care, despite much innovation.[17-22] In part this is attributable to the failure of current care provision to adequately integrate the needs or life contexts of young people within that care. Young people with T1DM need support to help them develop the psychosocial skills necessary to transition positively into an adult life with diabetes, and to enable them to engage productively with health care providers. However, the main focus in diabetes guidelines is on intensifying glucose levels with diabetes technologies and structured education.[23,24] Therefore, most current education programmes for young people aim to enhance self-management skills such as carbohydrate counting and insulin-dose-adjustment, to help optimise glucose levels; rather than addressing how to integrate living with diabetes in relation to the wider interpersonal and social challenges that they experience in this period. Hence, while these programmes are generally effective in the adult diabetes population, young people are less likely to participate in these programmes. A recent study of 227 young people identified that the main reasons for their non-participation was a lack of time and a preference for managing their diabetes on their own.[25] This indicates the need to reconsider both the type of educational support provided and how it is delivered to young people. While some programmes have been adapted for younger people, there is still an emphasis on technical aspects of diabetes,[26] rather than psychosocial adjustment.

Therefore, new educational programmes are required for young people, to ensure that what they learn about managing their diabetes is contextualised with learning to live with diabetes in relation to their social and emotional development. It is also important to explore innovative methods of delivering education to young people with T1DM to make programmes more relevant, attractive and accessible to them.

The Youth Empowerment Skills programme for young people with T1DM

The Youth Empowerment Skills (YES) programme is a novel psychoeducational intervention, which was developed through a co-design process with young people with the fundamental goal to overcome some of the challenges reviewed above. The co-design process sort to build the programme so that it addressed many of the previously identified challenges experienced

For peer review only - http://bmjopen.bmj.com/site/about/guidelines.xhtml

by young people with diabetes. The programme uses 3 well-established psychological theories, social learning theory,[27] self-regulatory theory[28] (personal identity) and dual-process theory.[29] These theories have been integrated into a learning model addressing: cognitive factors (knowledge, experiences, thinking processes and attitudes); environmental factors (exploring social norms and external factors that mediate behaviour); and behavioural factors (developing skills and self-efficiency, with practice, observation and rehearsal). This underpinning theory is delivered using different learning techniques: experiential and group-based learning; immersive simulations with scenarios such as treating an episode of severe hypoglycaemia or ketosis; and learning together in adventurous activities. The programme curriculum comprises the following sessions:

- Diabetes as a part of daily life;
- The psychological impact of diabetes;
- Staying safe while being away from home (parties/alcohol/drugs and diabetes);
- Immersive simulations (hypoglycaemia, DKA, impaired physical function);
- Reverse role-playing of health consultations (how to get more out of a consultation);
- Relationships (peers/partners/family);
- Attitudes towards food, weight and eating out;
- Getting to know your body (foot-care/eye screening/sexual health);
- Exercise and diabetes (activity-based sessions, e.g. rock climbing);
- How to prevent diabetes emergencies.

The programme is delivered by a peer educator with T1DM, health professionals and youth workers. The youth workers also do outreach work to identify potential participants and encourage them to attend. The programme is fully manualised and Quality Institute for Self-Management Education (QISMET) accredited.

As part of the programme's development, we undertook a systematic review to identify studies of psychosocially modelled educational interventions and youth worker deployment in young people with T1DM. We found a limited number of studies that incorporated some elements of the YES programme but none that shared the same theoretical approach with strategies explicitly focussed on the same target behaviours.[30-33] Hence, the YES programme is a distinct, co-designed and theoretically modelled intervention, with novel components. A

For peer review only - http://bmjopen.bmj.com/site/about/guidelines.xhtml

pilot evaluation of three early implementations of the YES programme that included 26 participants (mean age 18±1.7 years) showed it to be impactful on glycaemic control, with 46% (n=12) of participants achieving a clinically significant reduction in their HbA1c (≥5.5mmol/mol or 0.5% DCCT) at the 12-month follow-up. Programme uptake was 34% (n=26) with 96% (n=25) programme completion, which is similar to other programmes targeting young people.[25] Qualitative findings suggested that the programme had a positive psychosocial impact resulting in increased diabetes engagement and activation. The following active ingredients of the programme were also identified: social learning, peer support, relational care/support, and experiential learning. The youth worker was also highlighted by participants as being important in engaging them with the programme.

In the study outlined in this protocol, we propose to undertake a feasibility trial of the YES programme, with an integrated process evaluation. We have modelled the active ingredients of the programme to target outcomes related to psychosocial well-being, diabetes self-management activation and glycaemic control. In the process evaluation we will examine methodological, procedural and clinical uncertainties around the YES programme,[30-32] including: (1) estimates of likely recruitment and retention rates; (2) feasibility and acceptability of data collection instruments and data collection procedures; and (3) feasibility and acceptability of the programme. In line with standard feasibility study objectives,[31] we will not be testing the effect of the programme on outcomes, although we will estimate the impact of the programme on outcomes, to: consider the feasibility of progression to a definitive trial; and to inform a sample size calculation for such a trial. There will also be a mixed method element to the process evaluation,[32] to explore: (1) intervention acceptability; (2) the intervention experience and its impact on physical, mental and social well-being; (3) and parents' perspectives on the programme. These findings will be integrated to formulate potential mechanisms of change following programme exposure.

Aims and objectives

The aims of study are to: test the feasibility of the YES programme in terms of programme acceptance, implementability, recruitment and completion; and to estimate its efficacy in relation to clinical, psychosocial, and health care outcomes. The study objectives are to:

For peer review only - http://bmjopen.bmj.com/site/about/guidelines.xhtml

- Evaluate and optimise the research process: recruitment, willingness to be randomised, consent procedures, and data collection.
- Evaluate our ability to assess the programme's impact on self-management, patient activation, and clinical and psychosocial outcomes (optimising outcome selection and estimating the level effect).
- Establish the reach, fidelity, utility, feasibility, appropriateness, unintended consequences and sustainability of the YES intervention.
- Identify and explore the experiences of programme participants and those delivering the intervention.
- Optimise the intervention components, procedures and outcome measures.
- Obtain estimates of outcomes to inform the sample size calculation for a larger clinical evaluation study.

METHODS AND ANALYSIS

Study Design

The study has been designed as a feasibility waiting-list randomised controlled trial (RCT) with an integrated process evaluation. Participants will be randomised to YES exposure (phase 1) or a waiting list control and followed up for 6 months. At the 6 month follow up point, control participants will then be exposed to YES, in a phase 2 exposure. Both groups will be subsequently followed up for a further 6 months. This will ensure that all eligible young people will have the opportunity to participate in the programme while maintaining a controlled structure to YES exposure that will allow us to explore differences. Conducting an additional 6-month follow-up after phase 1, will also enable us to assess how sustainable the intervention is over a longer time period. The adoption of a waiting list design is important as it overcomes the ethical dilemma of denying control participants the opportunity to benefit from the programme. The study design is presented schematically in Figure 1.

(Figure 1 about here)

We have set the following progression criteria for a subsequent definitive trial: achieve minimum 30% recruitment of eligible participants; and ≥80% completion of final follow-up. We will also assess the impact of the intervention ingredients on target behaviours, including

For peer review only - http://bmjopen.bmj.com/site/about/guidelines.xhtml

activation of self-management, engagement in diabetes health care, and improved psychosocial functioning. These effects, alongside impact on glycaemic control, will be used to consider whether there is adequate potency in the programme and will determine whether a definitive trial will be conducted.

Setting

The study will be conducted in South London, United Kingdom, recruiting participants from the diabetes centres of five hospitals, providing care to the target population. These sites have been selected to ensure sociodemographic diversity, with a significant proportion of patients from lower socio-economic backgrounds and from Black Asian and Minority Ethnic (BAME) populations. The study is planned to start on 01 June 2021, and finish 31 March 2023.

Participants and sample size

We aim to recruit 50 young people and randomise half to the phase 1 programme exposure, and half to the phase 2 exposure. The inclusion criteria will involve:

- Age 14-19 years;
- Diagnosis of T1DM;
- Sub-optimal glycaemic control (HbA1c >69 mmol/mol or 8.5%);[3]
- No current or planned attendance of other structured education programmes.

Participants will be excluded if they: have a severe physical or mental illness, unstable retinopathy, significant learning difficulties; are unable to communicate in English; or are pregnant. Participants meeting the inclusion criteria will be recruited opportunistically at the participating centres through clinical referrals and patient database searches. Based on recruitment to the pilot study we estimate that we will be able to recruit >30% of eligible patients. To increase engagement, we will run the full study, including programme occurrences and follow-up sessions, during school holidays as this was identified by young people as the optimal period for running the YES programme.

As a feasibility evaluation, the study is not statistically powered to estimate a clinical effect. Instead, we aim to estimate the effect-size/standard deviation of outcomes to formulate a power calculation for a definitive trial. National Institute for Health Research (NIHR) guidance

For peer review only - http://bmjopen.bmj.com/site/about/guidelines.xhtml

indicates that samples of 24-50 are adequate for this purpose.[33-35] We have inflated our sample to allow for an estimated 20% drop-out rate.

Intervention and control conditions

The intervention is the YES programme as previously described. The programme exposure is sequenced as follows: (1) initial contact from the youth worker introducing the YES intervention; (2) recruitment to the study by researchers; (3) attendance at three all-day YES sessions in non-healthcare settings (groups of 10); (4) post-programme networking through social media and (5) follow-up events facilitated by the youth worker. Sessions will be led by a peer-educator and a health professional and delivered face-to-face. Participants in the waiting-list control arm of the study will receive usual care following initial randomisation and will be exposed to the YES intervention at 6 months (phase 2 exposure). Usual care in this patient population typically involves outpatient consultations. To minimise potential external confounding factors, we will monitor any other educational interactions that participants might experience during the follow-up period in both groups.

Randomisation

Participants will be randomised to either the phase 1 YES exposure or to the waiting list control (for phase 2 exposure), using computer generated randomisation in blocks of 20. This will be conducted by a member of the study team not involved in recruitment. As the intervention is open blinding is not possible. The waiting list design will help to maintain young people's motivation to participate in the phase 1 and 2 allocations. As part of our feasibility work, we will consider how acceptable randomisation is to young people with diabetes. Young people's lives are quite dynamic in this period with frequent changes such as moving schools starting further or higher education, or employment. Hence, while we will explain why randomisation is helpful to us, but if young people decline randomisation they will be offered a preference for when they join the programme.

Outcome measures

As a feasibility study, the objective is to optimise outcome selection and determine outcome effects prior to a definitive RCT. The primary outcome to assess the potential clinical effect of the programme will be HbA1c and we will examine: a) the number of participants

For peer review only - http://bmjopen.bmj.com/site/about/guidelines.xhtml

achieving clinically significant reductions of 5.5mmol/mol or more and mean differences between groups over time, and b) the standard deviation (SD) of the mean difference between the intervention and control group in HbA1c at 6 months; for the control group the SD of the mean difference between HbA1c at 12 months and pre-exposure (phase 2) and for the phase 1 intervention group the SD of the mean HbA1c difference between post (6 and 12 months) and pre-exposure. While HbA1c is a proxy measure of complication risk, it is the most commonly adopted measure for determining the short to medium term impact of an intervention and is the recommended measure from the National Institute for Health and Care Excellence (NICE) for assessing the clinical and health economic benefits of diabetes interventions. In addition, our preliminary work and logic model show that the programme can mediate health behaviours by impacting on the psychosocial factors that can mediate self-management activation leading to improved glucose control. Hence, we will also assess the psychosocial and behavioural impacts of the programme, using the following measures. For each outcome we will assess differences in pre-and post-exposure, as well as differences between control and intervention groups.

- Number of participants with 20% increase in frequency of blood glucose monitoring
- Insulin adherence measured by self-report scale
- Self-Management of Diabetes in Adolescence Scale (SMOD-A);[36]
- Confidence in managing own health care (Confidence in Diabetes Self-Care Scale (CDSS);[37]
- Diabetes Quality of Life Instrument (DQOL)-adapted for youth;[38]
- Illness perception (Brief Illness Perception Questionnaire, IPQ-B);[39]
- Emergency care events (ambulance call-outs, Accident and Emergency attendance or hospitalisations)
- Hypoglycaemia (blood glucose <3.5 mmol/L) and severe hypoglycaemic events (requiring third party assistance).
- Weight

Study procedure

Potential participants will be recruited from the participating diabetes centres. Since it can be challenging to engage young people with T1DM, especially those from deprived and

For peer review only - http://bmjopen.bmj.com/site/about/guidelines.xhtml

ethnically diverse groups, a youth worker role will be incorporated into the recruitment process to identify potential participants and support them in considering participation in the study. Young people will then be screened for eligibility and consented. Parental consent will also be gained from those aged <16 years of age. We will ask eligible young people who decline participation to give a reason for their decision. The participants will then be randomly allocated as previously described.

Data collection and storage

Data (questionnaires, body weight and medical-record data) will be collected at baseline, then again at 6 and 12 months. Baseline data will also include: socio-demographics; education/employment; and diabetes self-management behaviours including insulin use and blood glucose testing. Patient records will be examined to collect data on: HbA1c; hospitalisations and emergency care; diabetes education attendance; screening attendance; and attendance at appointments. For baseline this will be all events in the preceding 12 months, and post exposure will be for the 12 months following exposure (or 6 months' post exposure for those exposed at phase 2). Analysis will be on-treatment, as the primary aim of this study is to assess feasibility. Data will be coded and cleaned by statisticians and stored on an encrypted shared drive.

Data analysis

The data will be analysed to determine the proportion of patients achieving the progression criteria; and the target reduction in HbA1c from baseline to follow-up (6 and 12 months), which will be estimated in both intervention (YES) and control groups. We will also estimate the standard deviation (SD) of the mean difference between the intervention and control group in HbA1c at 6 months; for the control group the SD of the mean difference between HbA1c at 12 months and pre-exposure (Phase 2) and for the phase 1 intervention group the SD of the mean HbA1c difference between post (6 and 12 months) and pre-exposure. These data will help inform the sample size calculation for a definitive trial.

Process evaluation

The process evaluation has been designed in accordance with the MRC process evaluation model for complex interventions,[32] aiming to explore the setting, implementation, and

For peer review only - http://bmjopen.bmj.com/site/about/guidelines.xhtml

mechanisms of action of the programme to enable interpretation of the observed outcomes. Our process evaluation will also use an established taxonomy of outcomes to be evaluated for understanding implementation.[40-41] Following this taxonomy, we will assess the reach, fidelity; its perceived acceptability, appropriateness and feasibility; unintended consequences; and potential sustainability considerations. In addition, we will elicit potential mechanisms of action, including changes in behaviours, and how they relate to programme delivery and outcomes, using the COM-B (capability, opportunity, motivation to perform behaviour) and Behaviour Change Wheel (BCW) frameworks.[42-43] Further, we will apply the Consolidated Framework for Implementation Research (CFIR) to study how the programme can be optimally embedded within routine diabetes care (i.e. to capture systematically implementation and adoption drivers/barriers); and to optimise the design of a subsequent trial.[44]

The process evaluation will involve a mixed-method approach, including use of validated brief implementation outcome surveys[45] (to be delivered to both young people with diabetes and health providers involved in YES) and one-to-one interviews with young people, their relatives and healthcare professionals. The data collection tools and analytic models have been designed with reference to the BCW framework[43] and to provide the data for CFIR[44] analysis considering the barriers and facilitators to the intervention. To ensure relevance and feasibility, implementation data collection tools will be refined with the study patient and public representative groups. Semi-structured interviews exploring the implementation outcomes and programme impact within the first 3 months of the completion of the programme will be conducted with a random sub-sample of participants (n=12 from each arm of the study, 4 in each centre), including participating young peoples' relatives (n=10); and the healthcare professionals involved in the delivery of the programme (n=6). All interviews will be digitally recorded and transcribed verbatim in preparation for analysis using thematic analysis,[46] mapping the data using the BCW[43] and CFIR[44] frameworks.

To assess perception of how appropriate, acceptable and feasible the programme is to young people and healthcare professionals, we will use previously validated short pragmatic surveys.[45] This will allow quantitative assessment against the study end-points. We will also undertake:

For peer review only - http://bmjopen.bmj.com/site/about/guidelines.xhtml

- An audit of the number of eligible young people that decline participation, considering their clinical and sociodemographic profiles;
- An audit of intervention fidelity using an adherence checklist based on the YES protocol;
- Exit questionnaires with attending young people to rate programme satisfaction and utility; and to identify 3 strengths, weaknesses and areas for improvement.

The audit and questionnaire data will be analysed to examine the utilisation and satisfaction with the programme. Intervention fidelity will be assessed from the audit data detailing the delivery of the different intervention components. We will also monitor control conditions in the first 6 months to consider whether any extraneous interventions or clinical changes occurred that might impact on the observed outcomes. While the study will be recruiting participants from five diabetes centres, we do not anticipate significant site level effects on outcomes as the intervention will be delivered by the same clinicians and peer educators throughout, following the YES curriculum. However, we will monitor any variations in the general care for each centre, or any changes in the care provided to the target population during the follow-up period.

Health economic evaluation

While a full health economic analysis is beyond the scale of this feasibility study, we will collect data as part of the process evaluation to inform health economic analysis for a future definitive trial. The analysis will have 3 elements:

1. A full economic costing of the programme delivery costs.
2. An audit of medical records to consider health care utilisation and in particular impact on high-cost emergency care episodes.
3. Modelling cost benefits by considering the risk of long-term complications based on impact on glycaemic control using established health economic assumptions.[47]

Review and optimisation event

In the final phase of the study, we will organise a YES review event with a purposively selected group (age and gender) of participants (n=16), their family members or partners, health professionals from participating services and the research team. The aim of the event will be

For peer review only - http://bmjopen.bmj.com/site/about/guidelines.xhtml

to share study findings and collate feedback about the programme through facilitated round table discussions, in which participants will be encouraged to offer ideas to further improve the YES programme. Following this event, and based on an integrated assessment of the data collected, the intervention protocol and outcome measures will be revised for use in a definitive trial.

Patient and public involvement

A patient and public involvement (PPI) group consisting of young people who had previously attended the YES programme contributed to the development, design and conduct of the study (01/2018 to date), including: outcome measure selection; topic guide content for interviews; and formulating the information sheets and consent forms for ethics. In terms of outcome measures, the group advised us that only 3-4 questionnaires should be used as any more would be too burdensome and that this would affect quality of responses received. We showed the group 9 questionnaires which we related to the target psychosocial constructs and behaviours. They felt that many of the measures were too personal, confusing or distressing. Hence, we removed measures for: diabetes distress; diabetes stigma; and depression. We may, however, pick-up these themes in the interviews. We have recently expanded the PPI group to include some young people who have not attended the YES programme; and we have set up an additional group of parents of young people with T1DM. These groups will support the research team in the analysis, write-up and dissemination of study findings; and help in the process of optimising the programme and study procedures for a future trial.

PPI has also been important in helping prepare the study in relation to Covid-19 restrictions. Prior to starting the study, we conducted a survey of 74 young people with diabetes to explore whether they would be willing to meet in a group once the restrictions had lifted. Over half of the respondents (n=38) identified they were likely or very likely to attend a face-to-face education group or activity for young people with diabetes if it were offered to them. Three-quarters of the respondents (n=56) said that their views on attending YES had not been impacted by the pandemic.

Trial management

For peer review only - http://bmjopen.bmj.com/site/about/guidelines.xhtml

The study conduct and procedures will be overseen by an advisory board, comprising clinical stakeholders, the funder and PPI representatives. As a feasibility study of an intervention without hazard or potential for harm, there are no provision for stopping guidelines nor a specific data monitoring committee. Trial participants will receive follow-up support post-trial as part of their routine care. Any adverse events will be recorded and reported, and the study will be compliant with the hospital's safeguarding policy. Any changes to the protocol will be discussed with the study advisory board and reported to the research ethics committee, funder and trial participants. The final trial dataset will be accessed by the trial statisticians and researchers.

DISCUSSION

In this paper, we have described the protocol for a feasibility study to assess a complex psychoeducational intervention for young people with T1DM. As we have highlighted, there is a need to develop and test new educational approaches for young people with T1DM, which take account of the significant psychological and social challenges they experience in this period of their lives. The YES programme was co-designed with young people and has been theoretically modelled to target the challenges relevant to them, in order that they can adapt to living with diabetes. The study we have outlined here will help us establish whether the active mechanisms within the programme connect with how young people engage with their diabetes self-management as a condition for improving their glycaemic control, thereby reducing the risk of future complications. The study will also assess both quantitatively and qualitatively how the programme effects young people's orientation to their diabetes (positive or negative) psychologically and socially.

In conducting a trial of a clinical intervention, such as YES, it is important to consider how the intervention is experienced by those receiving and delivering it. Following the MRC model for complex interventions,[32] the process evaluation will assess the feasibility of the YES programme in terms of acceptance, implementability, recruitment and completion. Application of the COM-B[42] and BCW[43] frameworks will allow us to elicit/confirm the potential mechanisms of action and how they relate to programme delivery. This analysis will also help

For peer review only - http://bmjopen.bmj.com/site/about/guidelines.xhtml

us optimise the intervention components and outcome measures. Further, use of the CFIR framework to delineate barriers and drivers to YES implementation and Proctor et al's[40] taxonomy of implementation outcomes will allow systematic study of implementation aspects of YES, with both young people, their parents and T1DM care providers. These aspects of the study will be important in understanding how the intervention works in the context of its delivery and how it might be enhanced.[40] This evidence will be needed for the design of a subsequent definite trial of YES: based on the data that we collect on YES delivery, and if the implementation data collection of the current study, including both scales and qualitative data, is successful, it follows that a definite trial of YES may take a 'hybrid' format. Hybrid effectiveness-implementation studies include both clinical and implementation outcomes and evaluate both clinical effectiveness and implementation success of different strategies to deliver YES.[48] Such designs are efficient, in that they offer both clinical evidence and, simultaneously, evidence on what implementation strategies and support needs to be in place for YES to be scaled successfully outside the trial centres. The current study will provide the early information required for such a hybrid design to be applied.

Given the inherent complexity of the target population for the YES programme, running a clinical trial in this population will be challenging. Hence, the feasibility questions posed by the study in respect of recruitment, retention and data completeness will be important. We also hope that the qualitative work within the process evaluation will also allow us to learn how the research approach can be optimised prior to a full trial. A key consideration in relation to this will be the study design, particularly whether randomisation and the use of a waiting list model will be feasible. There is a growing recognition that conducting clinical trials in adolescents and young adults is challenging, with the suggestion that alternative more adaptive research models are needed.[49] While we have sort to address these challenges with the waiting list design and including the option for preference should participants decline randomisation; such adaptations can introduce bias in estimating observations.[50] While we cannot fully assess these biases in this study it will be important to consider whether, for example, those who decline randomisation and express a preference for either phase 1 or phase 2 exposure show stronger of weaker effects compared to those randomised. Following completion of the study, we will use the YES review event and our PPI groups to help us consider how best to design a definitive study should we meet our progression criteria.

For peer review only - http://bmjopen.bmj.com/site/about/guidelines.xhtml

ETHICS AND DISSEMINATION

The study described in this protocol has been reviewed and given favourable opinion by the London – Camden and Kings Cross Research Ethics Committee (REC number: 21/LO/0231; IRAS number: 279877) on April 20th 2021. Informed consent will be sought from all research participants for this study; a copy of the consent form (for participants >16 years of age) can be found in the Supplemental File 1 and a copy of the assent form (for participants <16 years of age) in the Supplemental File 2. The study will be conducted in accordance with Good Clinical Practice and recommendations for physicians involved in research on human subjects adopted by the 18th World Medical Assembly, Helsinki 1964 and later revisions. The study will produce key information to the stakeholders on the planning, funding and implementation of the interventions under investigation. Hence, the findings will be disseminated through peer-reviewed journals, relevant national and international conferences, as well as educational events at individual hospitals to ensure that they are brought to the appropriate stakeholders. We will also disseminate the findings to young people with diabetes and their families by sharing a lay summary of the findings on the Diabetes UK website and via relevant social media groups.

DATA SHARING STATEMENT

We will make anonymised data from the study available following a justified request for access.

FUNDING

The funding for this study was supported by the *Diabetes UK* (reference 19/0006055). The sponsor for the study is King's College London. TS is also supported by the Welcome Trust (219425/Z/19/Z). NS is funded by the *National Institute for Health Research* (NIHR) via the '*Applied Research Collaboration South London*' (ARC South London; grant number not available) at King's College Hospital National Health Service (NHS) Foundation Trust, London, UK. NS is a member of King's Improvement Science, which offers co-funding to the NIHR ARC South London and is funded by King's Health Partners (Guy's and St Thomas' NHS Foundation Trust, King's College Hospital NHS Foundation Trust, King's College London and South London and Maudsley NHS Foundation Trust), and the Guy's and St Thomas' Foundation. The funding

For peer review only - http://bmjopen.bmj.com/site/about/guidelines.xhtml

agreements ensured the authors' independence in designing the study, interpreting the data, writing, and publishing the report. The views expressed are those of the authors and not necessarily those of the NHS, the NIHR or the Department of Health and Social Care.

ACKNOWLEDGEMENTS

We would like to thank the representatives of the young people with T1DM, as well as the health care professionals involved, for their time and commitment in the design of this study protocol.

COMPETING INTERESTS

NS is the Director of London Safety & Training Solution ltd, which offers training in patient safety, implementation solutions and human factors to healthcare organisations and the pharmaceutical industry. TS received consultancy fees from Roche Diagnostics and Cancer Alliances.

AUTHORS' CONTRIBUTIONS

Dulmini Kariyawasam contributed to the intervention design and input on study design. Tayana Soukup contributed to the design of implementational science components of the study. Judith Parsons contributed to the study design, outcome selection and PPI (patient and public involvement) work. Nick Sevdalis contributed to the design of implementational science components of the study. Maria Baldellou Lopez contributed literature review to support study design. Rita Forde contributed to the study design, outcome selection and PPI work. Khalida Ismail contributed with the specialist advise on mental health aspects of the study and outcome selection. Marie Jones contributed to the intervention development. Martha Ford-Adams provided specialist advise on paediatric care for the study. Nardos Yemane contributed to intervention development. Siobhan Pender contributed to the intervention development. Stephen Thomas contributed to the intervention development. Trevor Murrells contributed with the statistical elements of the study design, data analysis and sample size. Alex Silverstien contributed with the intervention development and leads on the PPI aspect of the study. Angus Forbes led the design of the study and is being responsible for the overall content as guarantor. Tayana Soukup and Angus Forbes lead in the preparation

For peer review only - http://bmjopen.bmj.com/site/about/guidelines.xhtml

and writing of the manuscript with all co-authors involved in reviewing and revising the content.

LIST OF ABBREVIATIONS

BCW = Behaviour Change Wheel

CDSS = Confidence in Diabetes Self-Care Scale

COM-B = Capability, Opportunity, Motivation to Perform Behaviour

DKA = Diabetic-Keto-Acidosis

DQOL = Diabetes Quality of Life Instrument

HbA1c = Glycated Haemoglobin

IPQ-B = Illness Perception (Brief Illness Perception Questionnaire

MRC = Medical Research Council

NICE = National Institute for Health and Care Excellence

NIHR = National Institute for Health Research

NPT = Normalisation Process Theory

PPI = Patient and Public Involvement

RCT = Randomised Controlled Trial

SMOD-A = Self-Management of Diabetes in Adolescence Scale

T1DM = Type 1 Diabetes Mellitus

YES = Youth Empowerment Skills

For peer review only

For peer review only - http://bmjopen.bmj.com/site/about/guidelines.xhtml

REFERENCES

1. Hamilton J, Daneman D. Deteriorating diabetes control during adolescence: physiological or psychological? Journal of Pediatr Endocrinol Metab 2002;15(2):115-126.
2. Northam EA, Lin A, Finch S, Werther GA, Cameron FJ. Psychosocial well-being and functional outcomes in youth with Type 1 diabetes. Diabetes Care 2010;33(7):1430–1437.
3. National Paediatric Diabetes Audit. National Paediatric Diabetes Audit Report 2018-19. London: Royal College of Paediatrics and Child Health; 2019. Available: final_npda_core_report_2018-2019.pdf (rcpch.ac.uk)
4. White M, O'Connell MA, Cameron FJ. Transition to adult endocrine services: the diabetes perspective. Best Pract Res Clin Endocrinol Metab 2015;29(3): 497–504.
5. Gibb FW, Teoh WL, Graham J, Lockman KA. Risk of death following admission to a UK hospital with diabetic ketoacidosis. Diabetologia 2016;59(10):2082–2087.
6. Monaghan M, Helgeson V, Wiebe D. Type 1 diabetes in young adulthood. Curr Diabetes Rev 2015;11(4):239–250.
7. Oris L, Rassart J, Prikken S, Verschueren M, Goubert L Moons P, Berg CA, Weets I, Luyckx K. Illness identity in adolescents and emerging adults with Type 1 diabetes: introducing the Illness Identity Questionnaire. Diabetes Care 2016;39(5):757-763.
8. Luyckx K, Seiffge-Krenke I, Schwartz SJ, Goossens L, Weets I, Hendrieckx C, Groven C. Identity development, coping, and adjustment in emerging adults with a chronic illness: the sample case of Type 1 diabetes. Journal of Adolescent Health 2008;43(5):451-458.
9. Palladino DK, Helgeson VS, Reynolds KA, Becker DJ, Siminerio LM, Escobar O. Emerging adults with type 1 diabetes: a comparison to peers without diabetes. J Pediatr Psychol 2003;38(5):506–517.
10. Herzer M, Hood,K. Anxiety symptoms in adolescents with Type 1 diabetes: association with blood glucose monitoring and glycemic control. J Pediatr Psychol 2010;35(4):415–425.
11. Eating problems in adolescents with Type 1 diabetes: a systematic review with meta-analysis.Young V, Eiser C, Johnson B, Brierley S, Epton T, Elliott J, Heller S Diabet Med. 2013 Feb; 30(2):189-98.
12. Rawshani A, Sattar N, Franzen S, Rawshani A, Hattersley AT, Svensson AM, ELiassion B, Gudbjornsdottir S. Excess mortality and cardiovascular disease in young adults with Type 1

For peer review only - http://bmjopen.bmj.com/site/about/guidelines.xhtml

diabetes in relation to age at onset: a nationwide, register-based cohort study. Lancet 2018; 392:477–486.

13. Hains AA, Berlin KS, Davies WH, Parton EA, Alemzadeh R. Attributions of adolescents with type 1 diabetes in social situations: relationship with expected adherence, diabetes stress, and metabolic control. Diabetes Care 2006;29(4):818–822.
14. Whittemore R, Jaser S, Chao A, Grey M. Psychological experience of parents of children with Type 1 diabetes: a systematic mixed-studies review. Diabetes Educ 2012;38(4):562–579.
15. Eilander MMA, Snoek FJ, Rotteveel J, Aanstoot HJ, Bakker-van Waarde WM, Houdijk ECAM, Nuboer R, Winterdijk P, deWit M. Parental diabetes behaviors and distress are related to glycemic control in youth with Type 1 diabetes: longitudinal data from the DINO Study. J Diabetes Res 2017. Available: https://doi.org/ 10.1155/2017/1462064
16. Whittemore R, Liberti L, Jeon S, Chao A, Jaser S, Grey M. Self-management as a mediator of family functioning and depressive symptoms with health outcomes in youth with type 1 diabetes. West J Nurs Res 2014;36(9):1254–1271.
17. Khanolkar AR, Amin R, Taylor-Robinson D, Viner RM, Warner JT, Stephenson T. Young people with Type 1 diabetes of non-white ethnicity and lower socio-economic status have poorer glycaemic control in England and Wales. Diabet Med 2016;33(11):1508–1515.
18. Elders V, Keen A, Gold A. Adults with type 1 diabetes: what factors are associated with disengagement from health services? Practical Diabetes 2013;31(3):117-118a.
19. Snow R, Fulop N. Understanding issues associated with attending a young adult diabetes clinic: a case study. Diabet Med 2012;29(2):257-259.
20. Peters A, Lafell L, the American Diabetes Association Transitions Working Group. Diabetes care for emerging adults: recommendations for transition from pediatric to adult diabetes care systems: a position statement of the American Diabetes Association Diabetes Care. 2011;34(11):2477-2485.
21. Sheehan AM, While AE, Coyne I. The experiences and impact of transition from child to adult healthcare services for young people with type 1 diabetes: a systematic review. Diabet Med 2015;32(4):440-458.
22. Pyatak EA, Sequeira PA, Whittemore R, Vigen CP, Peters AL, Weigensberg MJ. Disrupted transition from paediatric to adult diabetes care in young adults with Type 1 diabetes. Diabet Med 2014;31(12):1615–1624.

For peer review only - http://bmjopen.bmj.com/site/about/guidelines.xhtml

23. National Institute for Health and Care Excellence (NICE). Type 1 diabetes in adults: diagnosis and management [NG17] London. Available: www.nice.org.uk/guidance/ng17.
24. National Institute for Health and Care Excellence (NICE). Diabetes (Type 1 and Type 2) in children and young people: diagnosis and management [NG18]. Available: https://www.nice.org.uk/guidance/ng18
25. Coates VE, Horigan G, Davies M, Davies MT. Exploring why young people with Type 1 diabetes decline structured education with a view to overcoming barriers. Diabet Med 2017;34(8):1092-1099.
26. Coates V, Chaney D, Bunting B, Shorter GW, Shevlin M. Evaluation of the Effectiveness of a Structured Diabetes Education Programme (CHOICE) on Clinical Outcomes for Adolescents with Type 1 Diabetes: A Randomised Controlled Trial. J Diabetes Metab 2013;4:280.
27. Bandura A. Social Learning Theory. Engelwood Cliffs, N.J.: Prentice Hall; 1977.
28. Leventhal H, Nerenz DR, Steele DJ. Illness Representations and Coping with Health Threats. In: Baum, A, Taylor SE, Singer JE. Eds., Handbook of Psychology and Health Volume IV: Social Psychological Aspects of Health. Hillsdale, NJ: Erlbaum; 1984.p.219-252.
29. Chaiken S, Wood W, Eagly AH. Principles of persuasion. In: Higgins ET, Kruglansksi AW. Eds., Social Psychology: Handbook of Basic Principles. New York, Guilford Press; 1996.
30. Skivington K, Matthews L, Simpson SA, et al. Framework for the development and evaluation of complex interventions: gap analysis, workshop and consultation-informed update. Health Technol Assess. 2021;25(57):1-132. doi:10.3310/hta25570
31. Eldridge SM, Lancaster GA, Campbell MJ, et al. Defining feasibility and pilot studies in preparation for randomised controlled trials: development of a conceptual framework. PLoSOne 2016;11:e0150205.doi:10.1371/journal.pone.0150205
32. Moore GF, Audrey S, Barker M, Bond L, Bonell C, Hardeman W, Moore L, O'Cathain A, Tinati T, Wight D, Daird J. Process evaluation of complex interventions: Medical Research Council guidance. BMJ (Clinical research ed.) 2015;350:h1258.
33. Sim J, Lewis M. The size of a pilot study for a clinical trial-considerations of precision and efficiency. J Clin Epidemiol 2012;65(3):301-308.
34. Hooper R. Justifying sample size for a feasibility study [Internet] London: National Institute for Health Research; 2019. Available from: https://www.rds-london.nihr.ac.uk/wpcms/wp-

For peer review only - http://bmjopen.bmj.com/site/about/guidelines.xhtml

content/uploads/2019/02/Justifying-sample-size-for-feasibility-study-updated-22-Feb-2019.pdf.

35. Julious SA. Sample size of 12 per group rule of thumb for a pilot study. Pharmaceut Statist 2005;4:287-291.

36. Schilling LS, Dixon JK, Knafl KA, Lynn MR, Murphy K, Dumser S, Grey M. A new self-report measure of self-management of type 1 diabetes for adolescents. Nurs Res 2009;58(4):228-236.

37. Van Der Ven NC, Weinger K, Yi J, Pouwer F, Ader H, Van Der Ploeg HM, Snoek FH. The confidence in diabetes self-care scale: psychometric properties of a new measure of diabetes-specific self-efficacy in Dutch and US patients with Type 1 diabetes. Diabetes Care 2003;26(3):713-718.

38. Ingersoll GM, Marrero DG. A modified quality-of-life measure for youths: psychometric properties. Diabetes Educ 1991;17(2):114–120.

39. Broadbent E, Petrie KJ, Main J, Weinman J. The brief illness perception questionnaire. J Psychosom Res 2006;60(6):631–637.

40. Proctor E, Silmere H, Raghavan R, Hovmand P, Aarons G, Bunger A, Griffey R, Hensley M. Outcomes for implementation research: Conceptual distinctions, measurement challenges, and research agenda. Adm Policy Ment Health 2011;38(2):65-76.

41. Powell BJ, Waltz TJ, Chinman MJ, Damschroder LJ, Smith JL, Matthieu MM, Proctor EK, Kirchner JE. A refined compilation of implementation strategies: results from the Expert Recommendations for Implementing Change (ERIC) project. Implementation Sci 2015;10:21.

42. Barker F, Atkins L, de Lusignan S. Applying the COM-B behaviour model and behaviour change wheel to develop an intervention to improve hearing-aid use in adult auditory rehabilitation. Int J Audiol 2016;55(sup3):S90-S98.

43. Michie S, Atkins L, West R. The Behaviour Change Wheel: a guide to designing interventions. Silverback Publishing; 2014.

44. Kirk MA, Kelley C, Yankey N, Birken SA, Abadie B, Damschroder L. A systematic review of the use of the Consolidated Framework for Implementation Research. Implement Sci 2016;11:72.

For peer review only - http://bmjopen.bmj.com/site/about/guidelines.xhtml

45. Weiner BJ, Lewis CC, Stanick C, Powell BJ, Dorsey CN, Clary AS, Boynton MH, Halko H. Psychometric assessment of three newly developed implementation outcome measures. Implementation Sci 2017;12:108.

46. Ritchie J, Lewis J, Nicholls CM, Ormston R. Qualitative Research Practice: A Guide for Social Science Students and Researchers: London: Sage.

47. National Institute for Health and Care Excellence (NICE). Diabetes (Type 1 and Type 2) in children and young people: diagnosis and management [NG18]. Section 20 Health economics. Available: https://www.ncbi.nlm.nih.gov/books/NBK315806/.

48. Kemp CG, Wagenaar BH, Haroz EE. Expanding hybrid studies for implementation research: Intervention, implementation strategy, and context. Front Public Health 2019;7:325.

49. Baiardi, P., Giaquinto, C., Girotto, S. et al. Innovative study design for paediatric clinical trials. Eur J Clin Pharmacol 67, 109–115 (2011). https://doi.org/10.1007/s00228-011-0990-y

50. Cunningham JA, Kypri K, McCambridge J. Exploratory randomized controlled trial evaluating the impact of a waiting list control design. BMC Med Res Methodol. 2013;13(1):150. doi:10.1186/1471-2288-13-150

For peer review only - http://bmjopen.bmj.com/site/about/guidelines.xhtml

FIGURE CAPTIONS

Figure 1. Consort diagram for the Youth Empowerment Skills (YES) Programme feasibility study

For peer review only

For peer review only - http://bmjopen.bmj.com/site/about/guidelines.xhtml

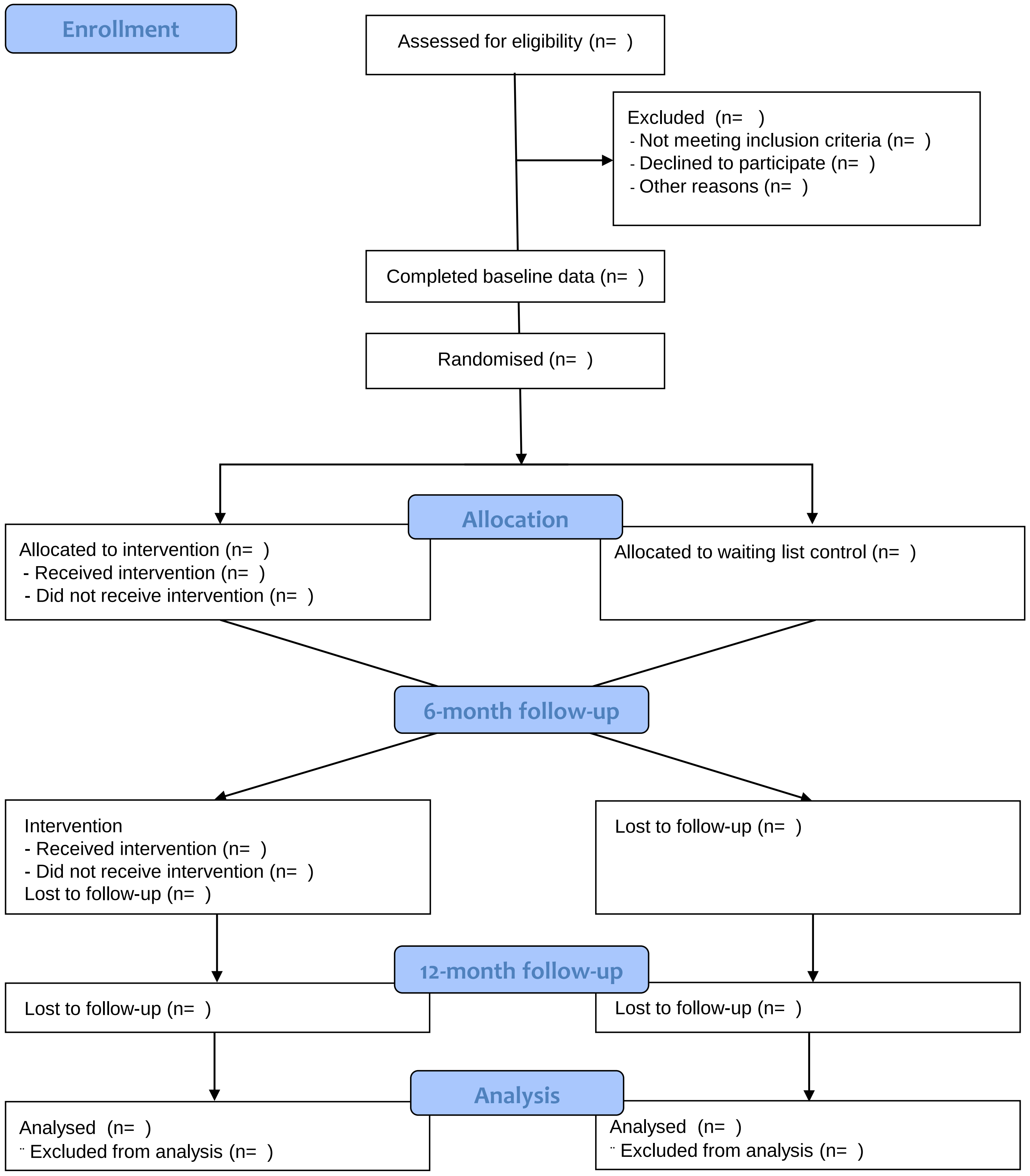
Enrollment
Assessed for eligibility (n=)
Excluded (n=)
- Not meeting inclusion criteria (n=)
- Declined to participate (n=)
- Other reasons (n=)
Completed baseline data (n=)
Randomised (n=)
Allocation
Allocated to intervention (n=)
- Received intervention (n=)
- Did not receive intervention (n=)
Allocated to waiting list control (n=)
6-month follow-up
Intervention
- Received intervention (n=)
- Did not receive intervention (n=)
Lost to follow-up (n=)
Lost to follow-up (n=)
12-month follow-up
Lost to follow-up (n=)
Lost to follow-up (n=)
Analysis
Analysed (n=)
Excluded from analysis (n=)
Analysed (n=)
Excluded from analysis (n=)

Guy's and St Thomas' NHS Foundation Trust

King's College London

IRAS ID:	279877
Centre Number:	
Participant Identification Number for this study:	

CONSENT FORM FOR YOUNG PERSON ≥16 (PART A)

Title of Project:	A feasibility study and process evaluation of a psychosocial education programme for young people with type 1 diabetes - the Youth Empowerment Skills (YES)
Name of Researcher:	

Initial each box

1. I have read the *Participant Information Sheet* dated................ I **understand what the research project is about**, what will happen if I take part and I have had all my questions answered. ☐
2. I understand that I do not have to take part, and I know that I can stop taking part at any time without giving a reason. I know that **this will not make a difference to the care I receive from my diabetes team.** ☐
3. I give permission for researchers from King's College London to **look at my medical notes**. This will only be to find the information relevant to the above research project (as mentioned in the participant information sheet), and **only for the duration of the research,** which will end in August 2022**.** ☐
4. I agree to fill in **questionnaires** twice during the research project. ☐
5. I agree to take part in an **interview** (if asked) about the YES programme. The interview will be **audio-recorded** but will only be listened to by the researcher. Some of the things I say in the interview may be written about in research reports, but this will be **anonymous** (no one will be able to know who said it). ☐
6. I understand that people will be **chosen at random to have an interview**, and that I may not be offered one. ☐
7. I understand that information I give will be kept **confidential** (private) and stored in a safe place for up to 12 years after the project ends. ☐
8. I understand the **limits to confidentiality** as stated in the *Participant Information Sheet*. For example, if the researchers are worried about my safety or the safety of others they may tell someone else. ☐
9. I understand that if I leave the study early, the researchers will **still use the information** I have already given them. However, this information will **not have my** ☐

For peer review only - http://bmjopen.bmj.com/site/about/guidelines.xhtml

Guy's and St Thomas' NHS Foundation Trust

KING'S College LONDON

name on. Any information about me that includes personal details will be removed from the research project and archived (stored).

10. I understand that if I leave the study early, I will be asked if I am willing to take part in an **interview**, so the researchers can develop an understanding of what things might make a young person leave the study. I do not have to participate in this if I do not wish to. ☐

11. I understand that, for **auditing and monitoring purposes**, data collected during the research project may be looked at by people from King's College London, from regulatory authorities, or from the NHS Trust, where it is relevant to my taking part in the study. I give permission to these people to look my records for this reason. ☐

12. I understand that my **usual hospital diabetes team** will know that I am taking part in this research project. ☐

13. I understand that my **general practitioner (GP)** will know that I am taking part in this research project. ☐

14. **I agree to take part in the above research project.** ☐

Please sign and date the following section

___________________________	_______________	_________________________
Name of participant	Date	Signature
___________________________	_______________	_________________________
Name of person taking consent	Date	Signature

One copy for the participant, one copy for the researcher and one copy for the medical notes

For peer review only - http://bmjopen.bmj.com/site/about/guidelines.xhtml

Guy's and St Thomas' NHS Foundation Trust

KING'S College LONDON

IRAS ID:	279877
Centre Number:	
Participant Identification Number for this study:	
Title of Project:	A feasibility study and process evaluation of a psychosocial education programme for young people with type 1 diabetes - the Youth Empowerment Skills (YES)
Name of Researcher:	

ADDITIONAL CONSENT FORM (PART B)

Part B asks if you agree for us to keep your contact details after this research project is finished. This is so we can contact you about future research you might want to take part in. It is fine if you do not want us to keep your contact details for this purpose. You **do not** have to sign Part B to take part in this research project.

If you choose not to sign Part B, we will remove your name and contact details from our contacts list when this research project is finished, and we will not contact you about other research in the future. If you do sign Part B, you can still change your mind and ask us to remove your name and contact details from our contacts list **at any time**.

Initial each box

1. I agree that researchers at King's College London can keep my name and contact details on the project contacts list for_____years after this research project is finished. ☐
2. I understand that my name and contact details will be saved on a computer at King's College London and will be protected with a password. Only the researchers directly involved in this research project will have the password to access this information. ☐
3. I agree that these researchers from King's College London can use this information to contact me in the future. They will only contact me to let me know about other research that I can take part in **if I want to**. ☐
4. I understand that my name and contact details will **not** be used for any reason except that described above (number 3.). ☐
5. I understand that I can ask for my name and contact details to be removed from the ☐

For peer review only - http://bmjopen.bmj.com/site/about/guidelines.xhtml

contacts list at King's College London at any time. I know who to contact if I want to remove my details. I understand that researchers will not be able to contact me in the future if I remove my details.

6. I understand that if I do not want King's College London to keep my name and contact details on the contacts list after this project is finished, or if I ask for them to be removed at a later date:
   - This will **not** affect my involvement in the above research project.
   - This will **not** affect the care I receive from my diabetes team.

Please sign and date the following section

___________________________	______________	_________________________
Name of participant	Date	Signature
___________________________	______________	_________________________
Name of person taking consent	Date	Signature

One copy for the participant, one copy for the researcher and one copy for the medical notes

For peer review only - http://bmjopen.bmj.com/site/about/guidelines.xhtml

Guy's and St Thomas' NHS Foundation Trust

KING'S College LONDON

IRAS ID:	
Centre Number:	
Participant Identification Number for this study:	
**ASSENT FORM FOR YOUNG PERSON <16 (PART A)**	
Title of Project:	A feasibility study and process evaluation of a psychosocial education programme for young people with type 1 diabetes - the Youth Empowerment Skills (YES)
Name of Researcher:	

Initial each box

1. I have read the *Participant Information Sheet* dated................ I **understand what the research project is about**, what will happen if I take part and I have had all my questions answered. ☐

2. I understand that I do not have to take part, and I know that I can stop taking part at any time without giving a reason. I know that **this will not make a difference to the care I receive from my diabetes team.** ☐

3. I give permission for researchers from King's College London to **look at my medical notes**. This will only be to find the information relevant to the above research project (as mentioned in the participant information sheet), and **only for the duration of the research,** which will end in August 2022**.** ☐

4. I agree to fill in **questionnaires** twice during the research project. ☐

5. I agree to take part in an **interview** (if asked) about the YES programme. The interview will be **audio-recorded** but will only be listened to by the researcher. Some of the things I say in the interview may be written about in research reports, but this will be **anonymous** (no one will be able to know who said it). ☐

6. I understand that people will be **chosen at random to have an interview**, and that I may not be offered one. ☐

7. I understand that information I give will be kept **confidential** (private) and stored in a safe place for up to twelve years after the project ends. ☐

8. I understand the **limits to confidentiality** as stated in the *Participant Information Sheet*. For example, if the researchers are worried about my safety or the safety of others they may tell someone else. ☐

9. I understand that if I leave the study early, the researchers will **still use the information** I have already given them. However, this information will **not have my name on**. Any information about me that includes personal details will be removed ☐

For peer review only - http://bmjopen.bmj.com/site/about/guidelines.xhtml

Guy's and St Thomas' NHS Foundation Trust

KING'S College LONDON

from the research project and archived (stored).

10. I understand that if I leave the study early, I will be asked if I am willing to take part in an **interview**, so the researchers can develop an understanding of what things might make a young person leave the study. I do not have to participate in this if I do not wish to. ☐

11. I understand that, for **auditing and monitoring purposes**, data collected during the research project may be looked at by people from King's College London, from regulatory authorities, or from the NHS Trust, where it is relevant to my taking part in the study. I give permission to these people to look my records for this reason. ☐

12. I understand that my **usual diabetes team at ……………………… Hospital** will know that I am taking part in this research project. ☐

13. I understand that my **general practitioner (GP)** will know that I am taking part in this research project. ☐

14. **I agree to take part in the above research project.** ☐

Please sign and date the following section:

___________________________	_______________	_________________________
Name of participant	Date	Signature
___________________________	_______________	_________________________
Name of person taking assent	Date	Signature

One copy for the participant, one copy for the researcher and one copy for the medical notes.

For peer review only - http://bmjopen.bmj.com/site/about/guidelines.xhtml

IRAS ID:	
Centre Number:	
Participant Identification Number for this study:	
Title of Project:	A feasibility study and process evaluation of a psychosocial education programme for young people with type 1 diabetes - the Youth Empowerment Skills (YES)
Name of Researcher:	

ADDITIONAL ASSENT FORM (PART B)

Part B asks if you agree for us to keep your contact details after this research project is finished. This is so we can contact you about future research you might want to take part in. It is fine if you do not want us to keep your contact details for this purpose. You **do not** have to sign Part B to take part in this research project.

If you choose not to sign Part B, we will remove your name and contact details from our contacts list when this research project is finished, and we will not contact you about other research in the future. If you do sign Part B, you can still change your mind and ask us to remove your name and contact details from our contacts list **at any time**.

Initial each box

1. I agree that researchers at King's College London can keep my name and contact details on the project contacts list for_____years after this research project is finished. ☐
2. I understand that my name and contact details will be saved on a computer at King's College London and will be protected with a password. Only the researchers directly involved in this research project will have the password to access this information. ☐
3. I agree that these researchers from King's College London can use this information to contact me in the future. I understand that they will always contact my parents first. They will only contact me to let me know about other research that I can take part in **if I want to**. ☐
4. I understand that my name and contact details will **not** be used for any reason except ☐

For peer review only - http://bmjopen.bmj.com/site/about/guidelines.xhtml

that described above (number 3).

5. I understand that I can ask for my name and contact details to be removed from the contacts list at King's College London at any time. I know who to contact if I want to remove my details. I understand that researchers will not be able to contact me in the future if I remove my details. ☐

6. I understand that if I do not want King's College London to keep my name and contact details on the contacts list after this project is finished, or if I ask for them to be removed at a later date:
   - This will **not** affect my involvement in the above research project. ☐
   - This will **not** affect the care I receive from my diabetes team. ☐

Please sign and date the following section:

___________________________	_______________	_________________________
Name of participant	Date	Signature
___________________________	_______________	_________________________
Name of person taking assent	Date	Signature

One copy for the participant, one copy for the researcher and one copy for the medical notes.

For peer review only - http://bmjopen.bmj.com/site/about/guidelines.xhtml

STANDARD PROTOCOL ITEMS: RECOMMENDATIONS FOR INTERVENTIONAL TRIALS

SPIRIT 2013 Checklist: Recommended items to address in a clinical trial protocol and related documents*

Section/item	Item No	Description	Page No in the MS
**Administrative information**			
Title	1	Descriptive title identifying the study design, population, interventions, and, if applicable, trial acronym	**1**
Trial registration	2a	Trial identifier and registry name. If not yet registered, name of intended registry	**2**
	2b	All items from the World Health Organization Trial Registration Data Set	**1-19**
Protocol version	3	Date and version identifier	**2**
Funding	4	Sources and types of financial, material, and other support	**18**
Roles and responsibilities	5a	Names, affiliations, and roles of protocol contributors	**1, 19**
	5b	Name and contact information for the trial sponsor	**18**
	5c	Role of study sponsor and funders, if any, in study design; collection, management, analysis, and interpretation of data; writing of the report; and the decision to submit the report for publication, including whether they will have ultimate authority over any of these activities	**18**
	5d	Composition, roles, and responsibilities of the coordinating centre, steering committee, endpoint adjudication committee, data management team, and other individuals or groups overseeing the trial, if applicable (see Item 21a for data monitoring committee)	**n/a**

For peer review only

For peer review only - http://bmjopen.bmj.com/site/about/guidelines.xhtml

**Introduction**			
Background and rationale	6a	Description of research question and justification for undertaking the trial, including summary of relevant studies (published and unpublished) examining benefits and harms for each intervention	**4-8**
	6b	Explanation for choice of comparators	**10**
Objectives	7	Specific objectives or hypotheses	**7-8**
Trial design	8	Description of trial design including type of trial (eg, parallel group, crossover, factorial, single group), allocation ratio, and framework (eg, superiority, equivalence, noninferiority, exploratory)	**8-9**
**Methods: Participants, interventions, and outcomes**			
Study setting	9	Description of study settings (eg, community clinic, academic hospital) and list of countries where data will be collected. Reference to where list of study sites can be obtained	**9**
Eligibility criteria	10	Inclusion and exclusion criteria for participants. If applicable, eligibility criteria for study centres and individuals who will perform the interventions (eg, surgeons, psychotherapists)	**9**
Interventions	11a	Interventions for each group with sufficient detail to allow replication, including how and when they will be administered	**9, 10-11**
	11b	Criteria for discontinuing or modifying allocated interventions for a given trial participant (eg, drug dose change in response to harms, participant request, or improving/worsening disease)	**n/a**
	11c	Strategies to improve adherence to intervention protocols, and any procedures for monitoring adherence (eg, drug tablet return, laboratory tests)	**n/a**
	11d	Relevant concomitant care and interventions that are permitted or prohibited during the trial	**n/a**

Outcomes	12	Primary, secondary, and other outcomes, including the specific measurement variable (eg, systolic blood pressure), analysis metric (eg, change from baseline, final value, time to event), method of aggregation (eg, median, proportion), and time point for each outcome. Explanation of the clinical relevance of chosen efficacy and harm outcomes is strongly recommended	**10-11**
Participant timeline	13	Time schedule of enrolment, interventions (including any run-ins and washouts), assessments, and visits for participants. A schematic diagram is highly recommended (see Figure)	**Figure 1**
Sample size	14	Estimated number of participants needed to achieve study objectives and how it was determined, including clinical and statistical assumptions supporting any sample size calculations	**9-10**
Recruitment	15	Strategies for achieving adequate participant enrolment to reach target sample size	**11**
**Methods: Assignment of interventions (for controlled trials)**			
Allocation:			
Sequence generation	16a	Method of generating the allocation sequence (eg, computer-generated random numbers), and list of any factors for stratification. To reduce predictability of a random sequence, details of any planned restriction (eg, blocking) should be provided in a separate document that is unavailable to those who enrol participants or assign interventions	**10**
Allocation concealment mechanism	16b	Mechanism of implementing the allocation sequence (eg, central telephone; sequentially numbered, opaque, sealed envelopes), describing any steps to conceal the sequence until interventions are assigned	**10**
Implementation	16c	Who will generate the allocation sequence, who will enrol participants, and who will assign participants to interventions	**10**
Blinding (masking)	17a	Who will be blinded after assignment to interventions (eg, trial participants, care providers, outcome assessors, data analysts), and how	**10**

For peer review only - http://bmjopen.bmj.com/site/about/guidelines.xhtml

	17b	If blinded, circumstances under which unblinding is permissible, and procedure for revealing a participant's allocated intervention during the trial	**10**
**Methods: Data collection, management, and analysis**			
Data collection methods	18a	Plans for assessment and collection of outcome, baseline, and other trial data, including any related processes to promote data quality (eg, duplicate measurements, training of assessors) and a description of study instruments (eg, questionnaires, laboratory tests) along with their reliability and validity, if known. Reference to where data collection forms can be found, if not in the protocol	**10-12**
	18b	Plans to promote participant retention and complete follow-up, including list of any outcome data to be collected for participants who discontinue or deviate from intervention protocols	**11-14**
Data management	19	Plans for data entry, coding, security, and storage, including any related processes to promote data quality (eg, double data entry; range checks for data values). Reference to where details of data management procedures can be found, if not in the protocol	**12**
Statistical methods	20a	Statistical methods for analysing primary and secondary outcomes. Reference to where other details of the statistical analysis plan can be found, if not in the protocol	**12-14**
	20b	Methods for any additional analyses (eg, subgroup and adjusted analyses)	**12-14**
	20c	Definition of analysis population relating to protocol non-adherence (eg, as randomised analysis), and any statistical methods to handle missing data (eg, multiple imputation)	**12**
**Methods: Monitoring**			
Data monitoring	21a	Composition of data monitoring committee (DMC); summary of its role and reporting structure; statement of whether it is independent from the sponsor and competing interests; and reference to where further details about its charter can be found, if not in the protocol. Alternatively, an explanation of why a DMC is not needed	**16**

For peer review only - http://bmjopen.bmj.com/site/about/guidelines.xhtml

	21b	Description of any interim analyses and stopping guidelines, including who will have access to these interim results and make the final decision to terminate the trial	**16**
Harms	22	Plans for collecting, assessing, reporting, and managing solicited and spontaneously reported adverse events and other unintended effects of trial interventions or trial conduct	**16**
Auditing	23	Frequency and procedures for auditing trial conduct, if any, and whether the process will be independent from investigators and the sponsor	**n/a**
**Ethics and dissemination**			
Research ethics approval	24	Plans for seeking research ethics committee/institutional review board (REC/IRB) approval	**18**
Protocol amendments	25	Plans for communicating important protocol modifications (eg, changes to eligibility criteria, outcomes, analyses) to relevant parties (eg, investigators, REC/IRBs, trial participants, trial registries, journals, regulators)	**16**
Consent or assent	26a	Who will obtain informed consent or assent from potential trial participants or authorised surrogates, and how (see Item 32)	**18**
	26b	Additional consent provisions for collection and use of participant data and biological specimens in ancillary studies, if applicable	**n/a**
Confidentiality	27	How personal information about potential and enrolled participants will be collected, shared, and maintained in order to protect confidentiality before, during, and after the trial	**18**
Declaration of interests	28	Financial and other competing interests for principal investigators for the overall trial and each study site	**19**
Access to data	29	Statement of who will have access to the final trial dataset, and disclosure of contractual agreements that limit such access for investigators	**16**

For peer review only

Ancillary and post-trial care	30	Provisions, if any, for ancillary and post-trial care, and for compensation to those who suffer harm from trial participation	**16**
Dissemination policy	31a	Plans for investigators and sponsor to communicate trial results to participants, healthcare professionals, the public, and other relevant groups (eg, via publication, reporting in results databases, or other data sharing arrangements), including any publication restrictions	**18**
	31b	Authorship eligibility guidelines and any intended use of professional writers	**19**
	31c	Plans, if any, for granting public access to the full protocol, participant-level dataset, and statistical code	**16**
**Appendices**			
Informed consent materials	32	Model consent form and other related documentation given to participants and authorised surrogates	**18**
Biological specimens	33	Plans for collection, laboratory evaluation, and storage of biological specimens for genetic or molecular analysis in the current trial and for future use in ancillary studies, if applicable	**n/a**

*It is strongly recommended that this checklist be read in conjunction with the SPIRIT 2013 Explanation & Elaboration for important clarification on the items. Amendments to the protocol should be tracked and dated. The SPIRIT checklist is copyrighted by the SPIRIT Group under the Creative Commons "Attribution-NonCommercial-NoDerivs 3.0 Unported" license.

For peer review only

BMJ Open

Protocol for A Feasibility Study and Process Evaluation of a Psychosocially Modelled Diabetes Education Programme for Young People with Type 1 Diabetes: the Youth Empowerment Skills (YES) Programme

Journal:	*BMJ Open*
Manuscript ID	bmjopen-2022-062971.R2
Article Type:	Protocol
Date Submitted by the Author:	11-May-2022

Complete List of Authors:	Kariyawasam, Dulmini; Guy's and Saint Thomas' Hospitals NHS Trust, Diabetes<br>Soukup, Tayana ; King's College London, Health Service and Population Research Department; King's College London<br>Parsons, Judith; King's College London Florence Nightingale School of Nursing and Midwifery, Adult Nursing<br>Sevdalis, Nick; King's College London<br>Baldellou Lopez, Maria ; King's College London<br>Forde, Rita; King's College London, Faculty of Nursing, Midwifery and Palliative Care<br>Ismail, Khalida; King's College London<br>Jones, Marie; Guy's Hospital<br>Ford-Adams, Martha; King's College Hospital NHS Foundation Trust, Diabetes Department<br>Yemane, Nardos; Guy's and St Thomas' NHS Foundation Trust, Diabetes Department<br>Pender, Siobhan; Guy's and Saint Thomas' NHS Foundation Trust Neurology Department<br>Thomas, Stephen; Guy's and St Thomas' Hospitals NHS Trust, Diabetes Department<br>Murrells, Trevor; King's College London, National Nursing Research Unit<br>Silverstien, Alex; King's College London<br>Forbes, Angus; King's College London, Division of Care in Long-term Conditions

**Primary Subject Heading**:	Diabetes and endocrinology
Secondary Subject Heading:	Diabetes and endocrinology, Evidence based practice, Paediatrics
Keywords:	DIABETES & ENDOCRINOLOGY, General diabetes < DIABETES & ENDOCRINOLOGY, General endocrinology < DIABETES & ENDOCRINOLOGY

For peer review only - http://bmjopen.bmj.com/site/about/guidelines.xhtml

Protocol for A Feasibility Study and Process Evaluation of a Psychosocially Modelled Diabetes Education Programme for Young People with Type 1 Diabetes: the Youth Empowerment Skills (YES) Programme

Dulmini Kariyawasam,[1] Tayana Soukup,[2-5*] Judith Parsons,[6] Nick Sevdalis,[2] Maria Baldellou Lopez,[2] Rita Forde,[6] Khalida Ismail,[7] Marie Jones,[1] Martha Ford-Adams,[8] Nardos Yemane,[1] Siobhan Pender,[1] Stephen Thomas,[1] Trevor Murrells,[6] Alex Silverstien, Angus Forbes[6]

Author affiliations

[1] Diabetes Department, Guy's and St Thomas' NHS Foundation Trust, London, UK

[2] Centre for Implementation Science, Health Service and Population Research Department, Institute of Psychiatry, Psychology and Neuroscience, King's College London, UK

[3] Institute of Global Health Innovation, Faculty of Medicine, Imperial College London, UK

[4] School of Health & Sport Sciences, University of Suffolk, UK

[5] Department of Epidemiology and Environmental Health, The State University of New York at Buffalo, USA

[6] Division of Care in Long-term Conditions, King's College London, UK

[7] Department of Psychological Medicine, Institute of Psychiatry, King's College London, UK

[8] Diabetes Department, King's College Hospital, London, UK

Full address for correspondence

*Dr Tayana Soukup, Centre for Implementation Science, Health Service & Population Research Department, King's College London, 16 De Crespigny Park, London SE5 8AF. Email: tayana.soukup@kcl.ac.uk Telephone Number: +44207 848 0272

Word count: 4538/4000

For peer review only

For peer review only - http://bmjopen.bmj.com/site/about/guidelines.xhtml

ABSTRACT (268/300w)

Introduction. Adolescence is a challenging period for young people with type 1 diabetes, associated with worsening glycaemia and care disengagement. Educational interventions in this period tend to focus on diabetes-specific skills, with less emphasis on the psychosocial challenges associated with diabetes experienced by young people. To address this limitation, we co-designed with young people a psychosocially modelled programme of diabetes education, named 'Youth Empowerment Skills' (YES). The programme aims to facilitate a positive adaptation to life with diabetes and engagement with diabetes care through peer-based learning, immersive simulations, and support from an outreach youth worker. Here we present a protocol for a feasibility study of the YES programme.

Methods and analysis. The study was designed following the Medical Research Council Complex Intervention Evaluation Framework to: test the feasibility (acceptance, implementability, recruitment and completion) of the YES programme; and estimate its efficacy in relation to metabolic and psychosocial outcomes. The study will take place in diabetes centres serving socio-culturally diverse populations. We will conduct a feasibility RCT (waiting-list design) with integrated process evaluation. Fifty young people with type 1 diabetes (aged 14-19 years) will be randomly allocated to either the YES intervention or a waiting-list control. Randomisation acceptability will be assessed with provision for a preference allocation. Outcomes will be evaluated at 6 months, at which point the waiting list participants will be exposed to the YES programme with further follow-up to 12 months. A simultaneous process evaluation will use a mixed-methods approach collecting qualitative and quantitative data. Study findings will be used to optimise the intervention components, outcome measures and recruitment methods to inform a subsequent definitive trial.

Ethics and dissemination. The protocol has ethical approval from the UK Health Research Authority (approval IRAS project ID:279877). Findings will be disseminated in multiple formats for lay and professional audiences.

Key words: Type 1 diabetes, young people, psychoeducational programme

Trial registration number: Clinicaltrials.gov identifier NCT04670198

Protocol date and version: 07 April 2021, v1.1

For peer review only - http://bmjopen.bmj.com/site/about/guidelines.xhtml

Strengths and limitation of the study

- The study will test a novel co-designed psychoeducational intervention for young people with Type 1 diabetes designed to help promote a positive emotional and social adaptation to a life with diabetes.
- An integrated process evaluation within the study will develop our understanding of how to enhance study procedures and gain insights into programme implementation for future studies.
- The randomised waiting-list design allows for the comparison of the intervention and usual care exposures without the ethical implications of not providing the intervention to the control group.
- The target population for this study can be resistant to healthcare interventions and have significant school and social commitments, hence the study will consider recruitment feasibility and the acceptability of randomisation versus preference for group allocation.

For peer review only

For peer review only - http://bmjopen.bmj.com/site/about/guidelines.xhtml

INTRODUCTION

Impact of T1DM on young people

Type 1 diabetes (T1DM) in adolescence and early adulthood can be a very challenging period in the lives of young people. During this period young people can begin to dissociate from their diabetes, leading to a decline in their glycaemic control and an increased risk of acute events such as recurrent diabetic-keto-acidosis (DKA).[1-5] Data from the most recent National Paediatric Diabetes audit in the United Kingdom showed that only 10% of young people aged 14-19 years achieved a glycated haemoglobin (HbA1c) within the current target range of ≤48mmol/mol(6.5%); and a fifth had a HbA1c >75mmol/mol(9%) which is associated with a high risk of diabetes complications.[3]

Furthermore, adolescence and early adulthood is also a period when young people are forming their personal and social identities, and diabetes can have a detrimental impact on their psychosocial development.[6,7] During this period, young people can develop negative emotional constructs about diabetes, as they see their diabetes as an impediment to social relationships and life opportunities.[8] Consequently, some young people may develop maladaptive coping strategies such as avoidance (omitting insulin and reducing their glucose monitoring), or psychological morbidities (fear, anxiety, depression and disordered eating behaviours).[9,10,11] Young people with diabetes can also feel stigmatised,[12] further driving negative coping strategies (e.g., not using insulin in public or running glucose levels high to avoid hypoglycaemia).

During this period young people are also developing their personal autonomy, which can lead to conflicts with parents who may find allowing the young person to take control over their diabetes self-management behaviours and health management challenging.[13] Relationships can become emotionally charged with conflict, excess vigilance and distress, difficulties that can exacerbate disengagement and are associated with poorer glycaemic control.[14,15] Negative interactions with diabetes health professionals have also been identified as contributing to young people disengaging from their care.[16]

For peer review only - http://bmjopen.bmj.com/site/about/guidelines.xhtml

Current care models for young people with T1DM

Developing effective diabetes care for young people with T1DM is challenging. The transition from child to adult care has long been identified as a period when young people can disengage from their care, despite much innovation.[17-22] In part this is attributable to the failure of current care provision to adequately integrate the needs or life contexts of young people within that care. Young people with T1DM need support to help them develop the psychosocial skills necessary to transition positively into an adult life with diabetes, and to enable them to engage productively with health care providers. However, the main focus in diabetes guidelines is on intensifying glucose levels with diabetes technologies and structured education.[23,24] Therefore, most current education programmes for young people aim to enhance self-management skills such as carbohydrate counting and insulin-dose-adjustment, to help optimise glucose levels; rather than addressing how to integrate living with diabetes in relation to the wider interpersonal and social challenges that they experience in this period. Hence, while these programmes are generally effective in the adult diabetes population, young people are less likely to participate in these programmes. A recent study of 227 young people identified that the main reasons for their non-participation was a lack of time and a preference for managing their diabetes on their own.[25] This indicates the need to reconsider both the type of educational support provided and how it is delivered to young people. While some programmes have been adapted for younger people, there is still an emphasis on technical aspects of diabetes,[26] rather than psychosocial adjustment.

Therefore, new educational programmes are required for young people, to ensure that what they learn about managing their diabetes is contextualised with learning to live with diabetes in relation to their social and emotional development. It is also important to explore innovative methods of delivering education to young people with T1DM to make programmes more relevant, attractive and accessible to them.

The Youth Empowerment Skills programme for young people with T1DM

The Youth Empowerment Skills (YES) programme is a novel psychoeducational intervention, which was developed through a co-design process with young people with the fundamental goal to overcome some of the challenges reviewed above. The co-design process sort to build the programme so that it addressed many of the previously identified challenges experienced

For peer review only - http://bmjopen.bmj.com/site/about/guidelines.xhtml

by young people with diabetes. The programme uses 3 well-established psychological theories, social learning theory,[27] self-regulatory theory[28] (personal identity) and dual-process theory.[29] These theories have been integrated into a learning model addressing: cognitive factors (knowledge, experiences, thinking processes and attitudes); environmental factors (exploring social norms and external factors that mediate behaviour); and behavioural factors (developing skills and self-efficiency, with practice, observation and rehearsal). This underpinning theory is delivered using different learning techniques: experiential and group-based learning; immersive simulations with scenarios such as treating an episode of severe hypoglycaemia or ketosis; and learning together in adventurous activities. The programme curriculum comprises the following sessions:

- Diabetes as a part of daily life;
- The psychological impact of diabetes;
- Staying safe while being away from home (parties/alcohol/drugs and diabetes);
- Immersive simulations (hypoglycaemia, DKA, impaired physical function);
- Reverse role-playing of health consultations (how to get more out of a consultation);
- Relationships (peers/partners/family);
- Attitudes towards food, weight and eating out;
- Getting to know your body (foot-care/eye screening/sexual health);
- Exercise and diabetes (activity-based sessions, e.g. rock climbing);
- How to prevent diabetes emergencies.

The programme is delivered by a peer educator with T1DM, health professionals and youth workers. The youth workers also do outreach work to identify potential participants and encourage them to attend. The programme is fully manualised and Quality Institute for Self-Management Education (QISMET) accredited.

As part of the programme's development, we undertook a systematic review to identify studies of psychosocially modelled educational interventions and youth worker deployment in young people with T1DM. We found a limited number of studies that incorporated some elements of the YES programme but none that shared the same theoretical approach with strategies explicitly focussed on the same target behaviours.[30-33] Hence, the YES programme is a distinct, co-designed and theoretically modelled intervention, with novel components. A

For peer review only - http://bmjopen.bmj.com/site/about/guidelines.xhtml

pilot evaluation of three early implementations of the YES programme that included 26 participants (mean age 18±1.7 years) showed it to be impactful on glycaemic control, with 46% (n=12) of participants achieving a clinically significant reduction in their HbA1c (≥5.5mmol/mol or 0.5% DCCT) at the 12-month follow-up. Programme uptake was 34% (n=26) with 96% (n=25) programme completion, which is similar to other programmes targeting young people.[25] Qualitative findings suggested that the programme had a positive psychosocial impact resulting in increased diabetes engagement and activation. The following active ingredients of the programme were also identified: social learning, peer support, relational care/support, and experiential learning. The youth worker was also highlighted by participants as being important in engaging them with the programme.

In the study outlined in this protocol, we propose to undertake a feasibility trial of the YES programme, with an integrated process evaluation. We have modelled the active ingredients of the programme to target outcomes related to psychosocial well-being, diabetes self-management activation and glycaemic control. In the process evaluation we will examine methodological, procedural and clinical uncertainties around the YES programme,[30-32] including: (1) estimates of likely recruitment and retention rates; (2) feasibility and acceptability of data collection instruments and data collection procedures; and (3) feasibility and acceptability of the programme. In line with standard feasibility study objectives,[31] we will not be testing the effect of the programme on outcomes, although we will estimate the impact of the programme on outcomes, to: consider the feasibility of progression to a definitive trial; and to inform a sample size calculation for such a trial. There will also be a mixed method element to the process evaluation,[32] to explore: (1) intervention acceptability; (2) the intervention experience and its impact on physical, mental and social well-being; (3) and parents' perspectives on the programme. These findings will be integrated to formulate potential mechanisms of change following programme exposure.

Aims and objectives

The aims of study are to: test the feasibility of the YES programme in terms of programme acceptance, implementability, recruitment and completion; and to estimate its efficacy in relation to clinical, psychosocial, and health care outcomes. The study objectives are to:

For peer review only - http://bmjopen.bmj.com/site/about/guidelines.xhtml

- Evaluate and optimise the research process: recruitment, willingness to be randomised, consent procedures, and data collection.
- Evaluate our ability to assess the programme's impact on self-management, patient activation, and clinical and psychosocial outcomes (optimising outcome selection and estimating the level effect).
- Establish the reach, fidelity, utility, feasibility, appropriateness, unintended consequences and sustainability of the YES intervention.
- Identify and explore the experiences of programme participants and those delivering the intervention.
- Optimise the intervention components, procedures and outcome measures.
- Obtain estimates of outcomes to inform the sample size calculation for a larger clinical evaluation study.

METHODS AND ANALYSIS

Study Design

The study has been designed as a feasibility waiting-list randomised controlled trial (RCT) with an integrated process evaluation. Participants will be randomised to YES exposure (phase 1) or a waiting list control and followed up for 6 months. At the 6 month follow up point, control participants will then be exposed to YES, in a phase 2 exposure. Both groups will be subsequently followed up for a further 6 months. This will ensure that all eligible young people will have the opportunity to participate in the programme while maintaining a controlled structure to YES exposure that will allow us to explore differences. Conducting an additional 6-month follow-up after phase 1, will also enable us to assess how sustainable the intervention is over a longer time period. The adoption of a waiting list design is important as it overcomes the ethical dilemma of denying control participants the opportunity to benefit from the programme. The study design is presented schematically in Figure 1.

(Figure 1 about here)

We have set the following progression criteria for a subsequent definitive trial: achieve minimum 30% recruitment of eligible participants; and ≥80% completion of final follow-up. We will also assess the impact of the intervention ingredients on target behaviours, including

For peer review only - http://bmjopen.bmj.com/site/about/guidelines.xhtml

activation of self-management, engagement in diabetes health care, and improved psychosocial functioning. These effects, alongside impact on glycaemic control, will be used to consider whether there is adequate potency in the programme and will determine whether a definitive trial will be conducted.

Setting

The study will be conducted in South London, United Kingdom, recruiting participants from the diabetes centres of five hospitals, providing care to the target population. These sites have been selected to ensure sociodemographic diversity, with a significant proportion of patients from lower socio-economic backgrounds and from Black Asian and Minority Ethnic (BAME) populations. The study is planned to start on 01 June 2021, and finish 31 March 2023.

Participants and sample size

We aim to recruit 50 young people and randomise half to the phase 1 programme exposure, and half to the phase 2 exposure. The inclusion criteria will involve:

- Age 14-19 years;
- Diagnosis of T1DM;
- Sub-optimal glycaemic control (HbA1c >69 mmol/mol or 8.5%);[3]
- No current or planned attendance of other structured education programmes.

Participants will be excluded if they: have a severe physical or mental illness, unstable retinopathy, significant learning difficulties; are unable to communicate in English; or are pregnant. Participants meeting the inclusion criteria will be recruited opportunistically at the participating centres through clinical referrals and patient database searches. Based on recruitment to the pilot study we estimate that we will be able to recruit >30% of eligible patients. To increase engagement, we will run the full study, including programme occurrences and follow-up sessions, during school holidays as this was identified by young people as the optimal period for running the YES programme.

As a feasibility evaluation, the study is not statistically powered to estimate a clinical effect. Instead, we aim to estimate the effect-size/standard deviation of outcomes to formulate a power calculation for a definitive trial. National Institute for Health Research (NIHR) guidance

For peer review only - http://bmjopen.bmj.com/site/about/guidelines.xhtml

indicates that samples of 24-50 are adequate for this purpose.[33-35] We have inflated our sample to allow for an estimated 20% drop-out rate.

Intervention and control conditions

The intervention is the YES programme as previously described. The programme exposure is sequenced as follows: (1) initial contact from the youth worker introducing the YES intervention; (2) recruitment to the study by researchers; (3) attendance at three all-day YES sessions in non-healthcare settings (groups of 10); (4) post-programme networking through social media and (5) follow-up events facilitated by the youth worker. Sessions will be led by a peer-educator and a health professional and delivered face-to-face. Participants in the waiting-list control arm of the study will receive usual care following initial randomisation and will be exposed to the YES intervention at 6 months (phase 2 exposure). Usual care in this patient population typically involves outpatient consultations. To minimise potential external confounding factors, we will monitor any other educational interactions that participants might experience during the follow-up period in both groups.

Randomisation

Participants will be randomised to either the phase 1 YES exposure or to the waiting list control (for phase 2 exposure), using computer generated randomisation in blocks of 20. This will be conducted by a member of the study team not involved in recruitment. As the intervention is open blinding is not possible. The waiting list design will help to maintain young people's motivation to participate in the phase 1 and 2 allocations. As part of our feasibility work, we will consider how acceptable randomisation is to young people with diabetes. Young people's lives are quite dynamic in this period with frequent changes such as moving schools starting further or higher education, or employment. Hence, while we will explain why randomisation is helpful to us, but if young people decline randomisation they will be offered a preference for when they join the programme.

Outcome measures

As a feasibility study, the objective is to optimise outcome selection and determine outcome effects prior to a definitive RCT. The primary outcome to assess the potential clinical effect of the programme will be HbA1c and we will examine: a) the number of participants achieving

For peer review only - http://bmjopen.bmj.com/site/about/guidelines.xhtml

clinically significant reductions of 5.5mmol/mol or more and mean differences between groups over time, and b) the standard deviation (SD) of the mean difference between the intervention and control group in HbA1c at 6 months; for the control group the SD of the mean difference between HbA1c at 12 months and pre-exposure (phase 2) and for the phase 1 intervention group the SD of the mean HbA1c difference between post (6 and 12 months) and pre-exposure. While HbA1c is a proxy measure of complication risk, it is the most commonly adopted measure for determining the short to medium term impact of an intervention and is the recommended measure from the National Institute for Health and Care Excellence (NICE) for assessing the clinical and health economic benefits of diabetes interventions. In addition, our preliminary work and logic model show that the programme can mediate health behaviours by impacting on the psychosocial factors that can mediate self-management activation leading to improved glucose control. Hence, we will also assess the psychosocial and behavioural impacts of the programme, using the following secondary outcome measures:

- Number of participants with 20% increase in frequency of blood glucose monitoring
- Insulin adherence measured by self-report scale
- Self-Management of Diabetes in Adolescence Scale (SMOD-A);[36]
- Confidence in managing own health care (Confidence in Diabetes Self-Care Scale (CDSS);[37]
- Diabetes Quality of Life Instrument (DQOL)-adapted for youth;[38]
- Illness perception (Brief Illness Perception Questionnaire, IPQ-B);[39]
- Emergency care events (ambulance call-outs, Accident and Emergency attendance or hospitalisations)
- Hypoglycaemia (blood glucose <3.5 mmol/L) and severe hypoglycaemic events (requiring third party assistance).
- Weight
- Intervention Appropriateness Measure (IAM)
- Feasibility of Intervention Measure (FIM)
- Acceptability of Intervention Measure (AIM)

For peer review only - http://bmjopen.bmj.com/site/about/guidelines.xhtml

For each outcome we will assess differences in pre-and post-exposure, as well as differences between control and intervention groups.

Study procedure

Potential participants will be recruited from the participating diabetes centres. Since it can be challenging to engage young people with T1DM, especially those from deprived and ethnically diverse groups, a youth worker role will be incorporated into the recruitment process to identify potential participants and support them in considering participation in the study. Young people will then be screened for eligibility and consented. Parental consent will also be gained from those aged <16 years of age. We will ask eligible young people who decline participation to give a reason for their decision. The participants will then be randomly allocated as previously described.

Data collection and storage

Data (questionnaires, body weight and medical-record data) will be collected at baseline, then again at 6 and 12 months. Baseline data will also include: socio-demographics; education/employment; and diabetes self-management behaviours including insulin use and blood glucose testing. Patient records will be examined to collect data on: HbA1c; hospitalisations and emergency care; diabetes education attendance; screening attendance; and attendance at appointments. For baseline this will be all events in the preceding 12 months, and post exposure will be for the 12 months following exposure (or 6 months' post exposure for those exposed at phase 2). Analysis will be on-treatment, as the primary aim of this study is to assess feasibility. Data will be coded and cleaned by statisticians and stored on an encrypted shared drive.

Data analysis

The data will be analysed to determine the proportion of patients achieving the progression criteria; and the target reduction in HbA1c from baseline to follow-up (6 and 12 months), which will be estimated in both intervention (YES) and control groups. We will also estimate the standard deviation (SD) of the mean difference between the intervention and control group in HbA1c at 6 months; for the control group the SD of the mean difference between HbA1c at 12 months and pre-exposure (Phase 2) and for the phase 1 intervention group the

For peer review only - http://bmjopen.bmj.com/site/about/guidelines.xhtml

SD of the mean HbA1c difference between post (6 and 12 months) and pre-exposure. These data will help inform the sample size calculation for a definitive trial.

Process evaluation

The process evaluation has been designed in accordance with the MRC process evaluation model for complex interventions,[32] aiming to explore the setting, implementation, and mechanisms of action of the programme to enable interpretation of the observed outcomes. Our process evaluation will also use an established taxonomy of outcomes to be evaluated for understanding implementation.[40-41] Following this taxonomy, we will assess the reach, fidelity; its perceived acceptability, appropriateness and feasibility; unintended consequences; and potential sustainability considerations. In addition, we will elicit potential mechanisms of action, including changes in behaviours, and how they relate to programme delivery and outcomes, using the COM-B (capability, opportunity, motivation to perform behaviour) and Behaviour Change Wheel (BCW) frameworks.[42-43] Further, we will apply the Consolidated Framework for Implementation Research (CFIR) to study how the programme can be optimally embedded within routine diabetes care (i.e. to capture systematically implementation and adoption drivers/barriers); and to optimise the design of a subsequent trial.[44]

The process evaluation will involve a mixed-method approach, including use of validated brief implementation outcome surveys[45] (to be delivered to both young people with diabetes and health providers involved in YES) and one-to-one interviews with young people, their relatives and healthcare professionals. The data collection tools and analytic models have been designed with reference to the BCW framework[43] and to provide the data for CFIR[44] analysis considering the barriers and facilitators to the intervention. To ensure relevance and feasibility, implementation data collection tools will be refined with the study patient and public representative groups. Semi-structured interviews exploring the implementation outcomes and programme impact within the first 3 months of the completion of the programme will be conducted with a random sub-sample of participants (n=12 from each arm of the study, 4 in each centre), including participating young peoples' relatives (n=10); and the healthcare professionals involved in the delivery of the programme (n=6). All interviews

For peer review only - http://bmjopen.bmj.com/site/about/guidelines.xhtml

will be digitally recorded and transcribed verbatim in preparation for analysis using thematic analysis,[46] mapping the data using the BCW[43] and CFIR[44] frameworks.

To assess perception of how appropriate, acceptable and feasible the programme is to young people and healthcare professionals, we will use previously validated short pragmatic surveys.[45] This will allow quantitative assessment against the study end-points. We will also undertake:

- An audit of the number of eligible young people that decline participation, considering their clinical and sociodemographic profiles;
- An audit of intervention fidelity using an adherence checklist based on the YES protocol;
- Exit questionnaires with attending young people to rate programme satisfaction and utility; and to identify 3 strengths, weaknesses and areas for improvement.

The audit and questionnaire data will be analysed to examine the utilisation and satisfaction with the programme. Intervention fidelity will be assessed from the audit data detailing the delivery of the different intervention components. We will also monitor control conditions in the first 6 months to consider whether any extraneous interventions or clinical changes occurred that might impact on the observed outcomes. While the study will be recruiting participants from five diabetes centres, we do not anticipate significant site level effects on outcomes as the intervention will be delivered by the same clinicians and peer educators throughout, following the YES curriculum. However, we will monitor any variations in the general care for each centre, or any changes in the care provided to the target population during the follow-up period.

Health economic evaluation

While a full health economic analysis is beyond the scale of this feasibility study, we will collect data as part of the process evaluation to inform health economic analysis for a future definitive trial. The analysis will have 3 elements:

1. A full economic costing of the programme delivery costs.
2. An audit of medical records to consider health care utilisation and in particular impact on high-cost emergency care episodes.

For peer review only - http://bmjopen.bmj.com/site/about/guidelines.xhtml

For peer review only

3. Modelling cost benefits by considering the risk of long-term complications based on impact on glycaemic control using established health economic assumptions.[47]

Review and optimisation event

In the final phase of the study, we will organise a YES review event with a purposively selected group (age and gender) of participants (n=16), their family members or partners, health professionals from participating services and the research team. The aim of the event will be to share study findings and collate feedback about the programme through facilitated round table discussions, in which participants will be encouraged to offer ideas to further improve the YES programme. Following this event, and based on an integrated assessment of the data collected, the intervention protocol and outcome measures will be revised for use in a definitive trial.

Patient and public involvement

A patient and public involvement (PPI) group consisting of young people who had previously attended the YES programme contributed to the development, design and conduct of the study (01/2018 to date), including: outcome measure selection; topic guide content for interviews; and formulating the information sheets and consent forms for ethics. In terms of outcome measures, the group advised us that only 3-4 questionnaires should be used as any more would be too burdensome and that this would affect quality of responses received. We showed the group 9 questionnaires which we related to the target psychosocial constructs and behaviours. They felt that many of the measures were too personal, confusing or distressing. Hence, we removed measures for: diabetes distress; diabetes stigma; and depression. We may, however, pick-up these themes in the interviews. We have recently expanded the PPI group to include some young people who have not attended the YES programme; and we have set up an additional group of parents of young people with T1DM. These groups will support the research team in the analysis, write-up and dissemination of study findings; and help in the process of optimising the programme and study procedures for a future trial.

PPI has also been important in helping prepare the study in relation to Covid-19 restrictions. Prior to starting the study, we conducted a survey of 74 young people with diabetes to explore

For peer review only - http://bmjopen.bmj.com/site/about/guidelines.xhtml

whether they would be willing to meet in a group once the restrictions had lifted. Over half of the respondents (n=38) identified they were likely or very likely to attend a face-to-face education group or activity for young people with diabetes if it were offered to them. Three-quarters of the respondents (n=56) said that their views on attending YES had not been impacted by the pandemic.

Trial management

The study conduct and procedures will be overseen by an advisory board, comprising clinical stakeholders, the funder and PPI representatives. As a feasibility study of an intervention without hazard or potential for harm, there are no provision for stopping guidelines nor a specific data monitoring committee. Trial participants will receive follow-up support post-trial as part of their routine care. Any adverse events will be recorded and reported, and the study will be compliant with the hospital's safeguarding policy. Any changes to the protocol will be discussed with the study advisory board and reported to the research ethics committee, funder and trial participants. The final trial dataset will be accessed by the trial statisticians and researchers.

DISCUSSION

In this paper, we have described the protocol for a feasibility study to assess a complex psychoeducational intervention for young people with T1DM. As we have highlighted, there is a need to develop and test new educational approaches for young people with T1DM, which take account of the significant psychological and social challenges they experience in this period of their lives. The YES programme was co-designed with young people and has been theoretically modelled to target the challenges relevant to them, in order that they can adapt to living with diabetes. The study we have outlined here will help us establish whether the active mechanisms within the programme connect with how young people engage with their diabetes self-management as a condition for improving their glycaemic control, thereby reducing the risk of future complications. The study will also assess both quantitatively and qualitatively how the programme effects young people's orientation to their diabetes (positive or negative) psychologically and socially.

For peer review only - http://bmjopen.bmj.com/site/about/guidelines.xhtml

In conducting a trial of a clinical intervention, such as YES, it is important to consider how the intervention is experienced by those receiving and delivering it. Following the MRC model for complex interventions,[32] the process evaluation will assess the feasibility of the YES programme in terms of acceptance, implementability, recruitment and completion. Application of the COM-B[42] and BCW[43] frameworks will allow us to elicit/confirm the potential mechanisms of action and how they relate to programme delivery. This analysis will also help us optimise the intervention components and outcome measures. Further, use of the CFIR framework to delineate barriers and drivers to YES implementation and Proctor et al's[40] taxonomy of implementation outcomes will allow systematic study of implementation aspects of YES, with both young people, their parents and T1DM care providers. These aspects of the study will be important in understanding how the intervention works in the context of its delivery and how it might be enhanced.[40] This evidence will be needed for the design of a subsequent definite trial of YES: based on the data that we collect on YES delivery, and if the implementation data collection of the current study, including both scales and qualitative data, is successful, it follows that a definite trial of YES may take a 'hybrid' format. Hybrid effectiveness-implementation studies include both clinical and implementation outcomes and evaluate both clinical effectiveness and implementation success of different strategies to deliver YES.[48] Such designs are efficient, in that they offer both clinical evidence and, simultaneously, evidence on what implementation strategies and support needs to be in place for YES to be scaled successfully outside the trial centres. The current study will provide the early information required for such a hybrid design to be applied.

Given the inherent complexity of the target population for the YES programme, running a clinical trial in this population will be challenging. Hence, the feasibility questions posed by the study in respect of recruitment, retention and data completeness will be important. We also hope that the qualitative work within the process evaluation will also allow us to learn how the research approach can be optimised prior to a full trial. A key consideration in relation to this will be the study design, particularly whether randomisation and the use of a waiting list model will be feasible. There is a growing recognition that conducting clinical trials in adolescents and young adults is challenging, with the suggestion that alternative more adaptive research models are needed.[49] While we have sort to address these challenges with the waiting list design and including the option for preference should participants decline

For peer review only

randomisation; such adaptations can introduce bias in estimating observations.[50] While we cannot fully assess these biases in this study it will be important to consider whether, for example, those who decline randomisation and express a preference for either phase 1 or phase 2 exposure show stronger of weaker effects compared to those randomised. Following completion of the study, we will use the YES review event and our PPI groups to help us consider how best to design a definitive study should we meet our progression criteria.

ETHICS AND DISSEMINATION

The study described in this protocol has been reviewed and given favourable opinion by the London – Camden and Kings Cross Research Ethics Committee (REC number: 21/LO/0231; IRAS number: 279877) on April 20th 2021. Informed consent will be sought from all research participants for this study; a copy of the consent form (for participants >16 years of age) can be found in the Supplemental File 1 and a copy of the assent form (for participants <16 years of age) in the Supplemental File 2. The study will be conducted in accordance with Good Clinical Practice and recommendations for physicians involved in research on human subjects adopted by the 18th World Medical Assembly, Helsinki 1964 and later revisions. The study will produce key information to the stakeholders on the planning, funding and implementation of the interventions under investigation. Hence, the findings will be disseminated through peer-reviewed journals, relevant national and international conferences, as well as educational events at individual hospitals to ensure that they are brought to the appropriate stakeholders. We will also disseminate the findings to young people with diabetes and their families by sharing a lay summary of the findings on the Diabetes UK website and via relevant social media groups.

DATA SHARING STATEMENT

We will make anonymised data from the study available following a justified request for access.

FUNDING

The funding for this study was supported by the *Diabetes UK* (reference 19/0006055). The sponsor for the study is King's College London. TS is also supported by the Welcome Trust

For peer review only - http://bmjopen.bmj.com/site/about/guidelines.xhtml

(219425/Z/19/Z). NS is funded by the *National Institute for Health Research* (NIHR) via the '*Applied Research Collaboration South London*' (ARC South London; grant number not available) at King's College Hospital National Health Service (NHS) Foundation Trust, London, UK. NS is a member of King's Improvement Science, which offers co-funding to the NIHR ARC South London and is funded by King's Health Partners (Guy's and St Thomas' NHS Foundation Trust, King's College Hospital NHS Foundation Trust, King's College London and South London and Maudsley NHS Foundation Trust), and the Guy's and St Thomas' Foundation. The funding agreements ensured the authors' independence in designing the study, interpreting the data, writing, and publishing the report. The views expressed are those of the authors and not necessarily those of the NHS, the NIHR or the Department of Health and Social Care.

ACKNOWLEDGEMENTS

We would like to thank the representatives of the young people with T1DM, as well as the health care professionals involved, for their time and commitment in the design of this study protocol.

COMPETING INTERESTS

NS is the Director of London Safety & Training Solution ltd, which offers training in patient safety, implementation solutions and human factors to healthcare organisations and the pharmaceutical industry. TS received consultancy fees from Roche Diagnostics and Cancer Alliances.

AUTHORS' CONTRIBUTIONS

Dulmini Kariyawasam contributed to the intervention design and input on study design. Tayana Soukup contributed to the design of implementational science components of the study. Judith Parsons contributed to the study design, outcome selection and PPI (patient and public involvement) work. Nick Sevdalis contributed to the design of implementational science components of the study. Maria Baldellou Lopez contributed literature review to support study design. Rita Forde contributed to the study design, outcome selection and PPI work. Khalida Ismail contributed with the specialist advise on mental health aspects of the study and outcome selection. Marie Jones contributed to the intervention development.

For peer review only

For peer review only - http://bmjopen.bmj.com/site/about/guidelines.xhtml

Martha Ford-Adams provided specialist advise on paediatric care for the study. Nardos Yemane contributed to intervention development. Siobhan Pender contributed to the intervention development. Stephen Thomas contributed to the intervention development. Trevor Murrells contributed with the statistical elements of the study design, data analysis and sample size. Alex Silverstien contributed with the intervention development and leads on the PPI aspect of the study. Angus Forbes led the design of the study and is being responsible for the overall content as guarantor. Tayana Soukup and Angus Forbes lead in the preparation and writing of the manuscript with all co-authors involved in reviewing and revising the content.

LIST OF ABBREVIATIONS

BCW = Behaviour Change Wheel

CDSS = Confidence in Diabetes Self-Care Scale

COM-B = Capability, Opportunity, Motivation to Perform Behaviour

DKA = Diabetic-Keto-Acidosis

DQOL = Diabetes Quality of Life Instrument

HbA1c = Glycated Haemoglobin

IPQ-B = Illness Perception (Brief Illness Perception Questionnaire

MRC = Medical Research Council

NICE = National Institute for Health and Care Excellence

NIHR = National Institute for Health Research

NPT = Normalisation Process Theory

PPI = Patient and Public Involvement

RCT = Randomised Controlled Trial

SMOD-A = Self-Management of Diabetes in Adolescence Scale

T1DM = Type 1 Diabetes Mellitus

YES = Youth Empowerment Skills

For peer review only

For peer review only - http://bmjopen.bmj.com/site/about/guidelines.xhtml

REFERENCES

1. Hamilton J, Daneman D. Deteriorating diabetes control during adolescence: physiological or psychological? Journal of Pediatr Endocrinol Metab 2002;15(2):115-126.
2. Northam EA, Lin A, Finch S, Werther GA, Cameron FJ. Psychosocial well-being and functional outcomes in youth with Type 1 diabetes. Diabetes Care 2010;33(7):1430–1437.
3. National Paediatric Diabetes Audit. National Paediatric Diabetes Audit Report 2018-19. London: Royal College of Paediatrics and Child Health; 2019. Available: final_npda_core_report_2018-2019.pdf (rcpch.ac.uk)
4. White M, O'Connell MA, Cameron FJ. Transition to adult endocrine services: the diabetes perspective. Best Pract Res Clin Endocrinol Metab 2015;29(3): 497–504.
5. Gibb FW, Teoh WL, Graham J, Lockman KA. Risk of death following admission to a UK hospital with diabetic ketoacidosis. Diabetologia 2016;59(10):2082–2087.
6. Monaghan M, Helgeson V, Wiebe D. Type 1 diabetes in young adulthood. Curr Diabetes Rev 2015;11(4):239–250.
7. Oris L, Rassart J, Prikken S, Verschueren M, Goubert L Moons P, Berg CA, Weets I, Luyckx K. Illness identity in adolescents and emerging adults with Type 1 diabetes: introducing the Illness Identity Questionnaire. Diabetes Care 2016;39(5):757-763.
8. Luyckx K, Seiffge-Krenke I, Schwartz SJ, Goossens L, Weets I, Hendrieckx C, Groven C. Identity development, coping, and adjustment in emerging adults with a chronic illness: the sample case of Type 1 diabetes. Journal of Adolescent Health 2008;43(5):451-458.
9. Palladino DK, Helgeson VS, Reynolds KA, Becker DJ, Siminerio LM, Escobar O. Emerging adults with type 1 diabetes: a comparison to peers without diabetes. J Pediatr Psychol 2003;38(5):506–517.
10. Herzer M, Hood,K. Anxiety symptoms in adolescents with Type 1 diabetes: association with blood glucose monitoring and glycemic control. J Pediatr Psychol 2010;35(4):415–425.
11. Eating problems in adolescents with Type 1 diabetes: a systematic review with meta-analysis.Young V, Eiser C, Johnson B, Brierley S, Epton T, Elliott J, Heller S Diabet Med. 2013 Feb; 30(2):189-98.
12. Rawshani A, Sattar N, Franzen S, Rawshani A, Hattersley AT, Svensson AM, ELiassion B, Gudbjornsdottir S. Excess mortality and cardiovascular disease in young adults with Type 1

For peer review only - http://bmjopen.bmj.com/site/about/guidelines.xhtml

diabetes in relation to age at onset: a nationwide, register-based cohort study. Lancet 2018; 392:477–486.

13. Hains AA, Berlin KS, Davies WH, Parton EA, Alemzadeh R. Attributions of adolescents with type 1 diabetes in social situations: relationship with expected adherence, diabetes stress, and metabolic control. Diabetes Care 2006;29(4):818–822.
14. Whittemore R, Jaser S, Chao A, Grey M. Psychological experience of parents of children with Type 1 diabetes: a systematic mixed-studies review. Diabetes Educ 2012;38(4):562–579.
15. Eilander MMA, Snoek FJ, Rotteveel J, Aanstoot HJ, Bakker-van Waarde WM, Houdijk ECAM, Nuboer R, Winterdijk P, deWit M. Parental diabetes behaviors and distress are related to glycemic control in youth with Type 1 diabetes: longitudinal data from the DINO Study. J Diabetes Res 2017. Available: https://doi.org/ 10.1155/2017/1462064
16. Whittemore R, Liberti L, Jeon S, Chao A, Jaser S, Grey M. Self-management as a mediator of family functioning and depressive symptoms with health outcomes in youth with type 1 diabetes. West J Nurs Res 2014;36(9):1254–1271.
17. Khanolkar AR, Amin R, Taylor-Robinson D, Viner RM, Warner JT, Stephenson T. Young people with Type 1 diabetes of non-white ethnicity and lower socio-economic status have poorer glycaemic control in England and Wales. Diabet Med 2016;33(11):1508–1515.
18. Elders V, Keen A, Gold A. Adults with type 1 diabetes: what factors are associated with disengagement from health services? Practical Diabetes 2013;31(3):117-118a.
19. Snow R, Fulop N. Understanding issues associated with attending a young adult diabetes clinic: a case study. Diabet Med 2012;29(2):257-259.
20. Peters A, Lafell L, the American Diabetes Association Transitions Working Group. Diabetes care for emerging adults: recommendations for transition from pediatric to adult diabetes care systems: a position statement of the American Diabetes Association Diabetes Care. 2011;34(11):2477-2485.
21. Sheehan AM, While AE, Coyne I. The experiences and impact of transition from child to adult healthcare services for young people with type 1 diabetes: a systematic review. Diabet Med 2015;32(4):440-458.
22. Pyatak EA, Sequeira PA, Whittemore R, Vigen CP, Peters AL, Weigensberg MJ. Disrupted transition from paediatric to adult diabetes care in young adults with Type 1 diabetes. Diabet Med 2014;31(12):1615–1624.

For peer review only - http://bmjopen.bmj.com/site/about/guidelines.xhtml

23. National Institute for Health and Care Excellence (NICE). Type 1 diabetes in adults: diagnosis and management [NG17] London. Available: www.nice.org.uk/guidance/ng17.
24. National Institute for Health and Care Excellence (NICE). Diabetes (Type 1 and Type 2) in children and young people: diagnosis and management [NG18]. Available: https://www.nice.org.uk/guidance/ng18
25. Coates VE, Horigan G, Davies M, Davies MT. Exploring why young people with Type 1 diabetes decline structured education with a view to overcoming barriers. Diabet Med 2017;34(8):1092-1099.
26. Coates V, Chaney D, Bunting B, Shorter GW, Shevlin M. Evaluation of the Effectiveness of a Structured Diabetes Education Programme (CHOICE) on Clinical Outcomes for Adolescents with Type 1 Diabetes: A Randomised Controlled Trial. J Diabetes Metab 2013;4:280.
27. Bandura A. Social Learning Theory. Engelwood Cliffs, N.J.: Prentice Hall; 1977.
28. Leventhal H, Nerenz DR, Steele DJ. Illness Representations and Coping with Health Threats. In: Baum, A, Taylor SE, Singer JE. Eds., Handbook of Psychology and Health Volume IV: Social Psychological Aspects of Health. Hillsdale, NJ: Erlbaum; 1984.p.219-252.
29. Chaiken S, Wood W, Eagly AH. Principles of persuasion. In: Higgins ET, Kruglansksi AW. Eds., Social Psychology: Handbook of Basic Principles. New York, Guilford Press; 1996.
30. Skivington K, Matthews L, Simpson SA, et al. Framework for the development and evaluation of complex interventions: gap analysis, workshop and consultation-informed update. Health Technol Assess. 2021;25(57):1-132. doi:10.3310/hta25570
31. Eldridge SM, Lancaster GA, Campbell MJ, et al. Defining feasibility and pilot studies in preparation for randomised controlled trials: development of a conceptual framework. PLoSOne 2016;11:e0150205.doi:10.1371/journal.pone.0150205
32. Moore GF, Audrey S, Barker M, Bond L, Bonell C, Hardeman W, Moore L, O'Cathain A, Tinati T, Wight D, Daird J. Process evaluation of complex interventions: Medical Research Council guidance. BMJ (Clinical research ed.) 2015;350:h1258.
33. Sim J, Lewis M. The size of a pilot study for a clinical trial-considerations of precision and efficiency. J Clin Epidemiol 2012;65(3):301-308.
34. Hooper R. Justifying sample size for a feasibility study [Internet] London: National Institute for Health Research; 2019. Available from: https://www.rds-london.nihr.ac.uk/wpcms/wp-

For peer review only - http://bmjopen.bmj.com/site/about/guidelines.xhtml

content/uploads/2019/02/Justifying-sample-size-for-feasibility-study-updated-22-Feb-2019.pdf.

35. Julious SA. Sample size of 12 per group rule of thumb for a pilot study. Pharmaceut Statist 2005;4:287-291.

36. Schilling LS, Dixon JK, Knafl KA, Lynn MR, Murphy K, Dumser S, Grey M. A new self-report measure of self-management of type 1 diabetes for adolescents. Nurs Res 2009;58(4):228-236.

37. Van Der Ven NC, Weinger K, Yi J, Pouwer F, Ader H, Van Der Ploeg HM, Snoek FH. The confidence in diabetes self-care scale: psychometric properties of a new measure of diabetes-specific self-efficacy in Dutch and US patients with Type 1 diabetes. Diabetes Care 2003;26(3):713-718.

38. Ingersoll GM, Marrero DG. A modified quality-of-life measure for youths: psychometric properties. Diabetes Educ 1991;17(2):114–120.

39. Broadbent E, Petrie KJ, Main J, Weinman J. The brief illness perception questionnaire. J Psychosom Res 2006;60(6):631–637.

40. Proctor E, Silmere H, Raghavan R, Hovmand P, Aarons G, Bunger A, Griffey R, Hensley M. Outcomes for implementation research: Conceptual distinctions, measurement challenges, and research agenda. Adm Policy Ment Health 2011;38(2):65-76.

41. Powell BJ, Waltz TJ, Chinman MJ, Damschroder LJ, Smith JL, Matthieu MM, Proctor EK, Kirchner JE. A refined compilation of implementation strategies: results from the Expert Recommendations for Implementing Change (ERIC) project. Implementation Sci 2015;10:21.

42. Barker F, Atkins L, de Lusignan S. Applying the COM-B behaviour model and behaviour change wheel to develop an intervention to improve hearing-aid use in adult auditory rehabilitation. Int J Audiol 2016;55(sup3):S90-S98.

43. Michie S, Atkins L, West R. The Behaviour Change Wheel: a guide to designing interventions. Silverback Publishing; 2014.

44. Kirk MA, Kelley C, Yankey N, Birken SA, Abadie B, Damschroder L. A systematic review of the use of the Consolidated Framework for Implementation Research. Implement Sci 2016;11:72.

For peer review only - http://bmjopen.bmj.com/site/about/guidelines.xhtml

45. Weiner BJ, Lewis CC, Stanick C, Powell BJ, Dorsey CN, Clary AS, Boynton MH, Halko H. Psychometric assessment of three newly developed implementation outcome measures. Implementation Sci 2017;12:108.

46. Ritchie J, Lewis J, Nicholls CM, Ormston R. Qualitative Research Practice: A Guide for Social Science Students and Researchers: London: Sage.

47. National Institute for Health and Care Excellence (NICE). Diabetes (Type 1 and Type 2) in children and young people: diagnosis and management [NG18]. Section 20 Health economics. Available: https://www.ncbi.nlm.nih.gov/books/NBK315806/.

48. Kemp CG, Wagenaar BH, Haroz EE. Expanding hybrid studies for implementation research: Intervention, implementation strategy, and context. Front Public Health 2019;7:325.

49. Baiardi, P., Giaquinto, C., Girotto, S. et al. Innovative study design for paediatric clinical trials. Eur J Clin Pharmacol 67, 109–115 (2011). https://doi.org/10.1007/s00228-011-0990-y

50. Cunningham JA, Kypri K, McCambridge J. Exploratory randomized controlled trial evaluating the impact of a waiting list control design. BMC Med Res Methodol. 2013;13(1):150. doi:10.1186/1471-2288-13-150

For peer review only - http://bmjopen.bmj.com/site/about/guidelines.xhtml

For peer review only

FIGURE CAPTIONS

Figure 1. Consort diagram for the Youth Empowerment Skills (YES) Programme feasibility study

For peer review only

For peer review only - http://bmjopen.bmj.com/site/about/guidelines.xhtml

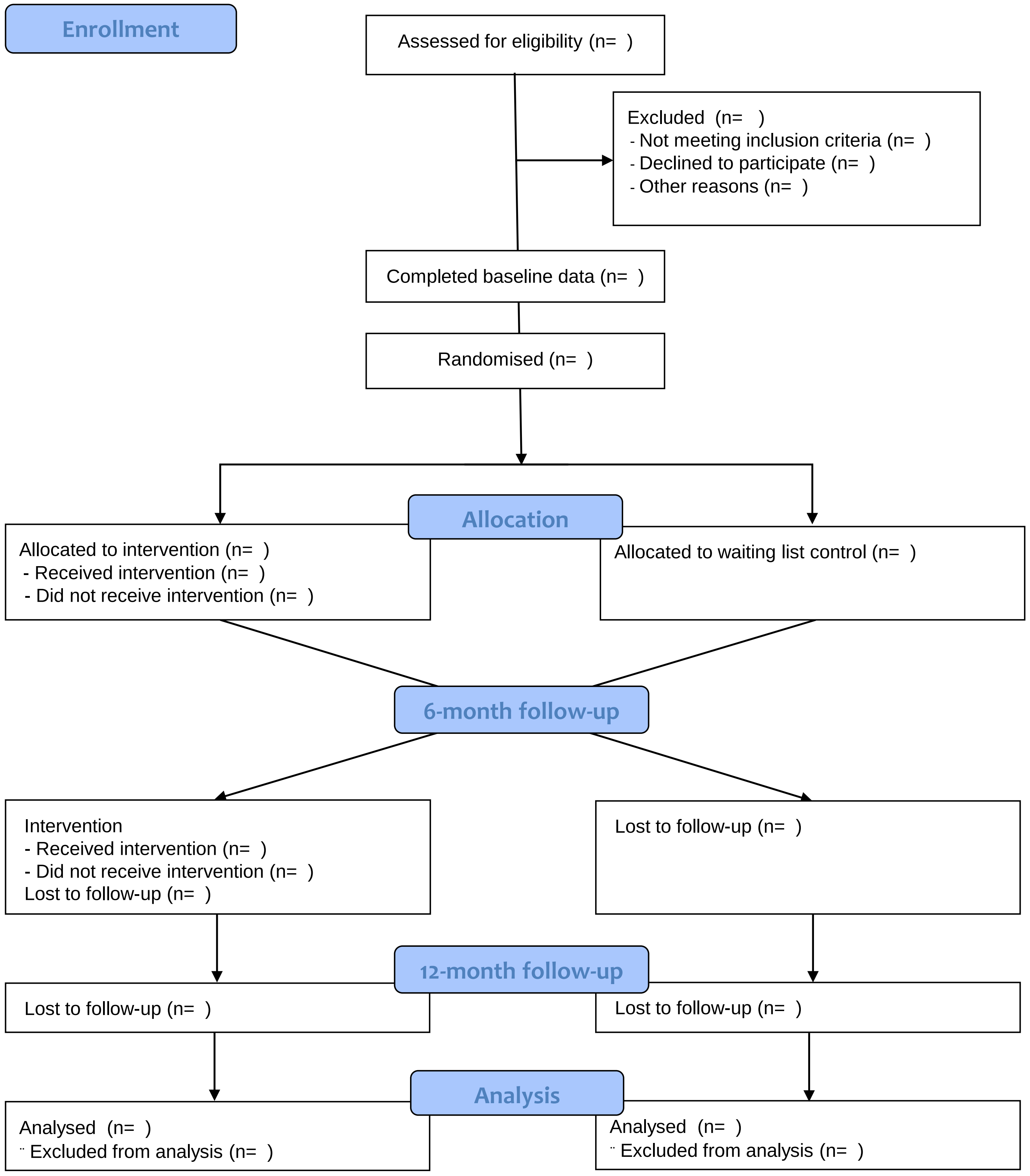
Enrollment
Assessed for eligibility (n=)
Excluded (n=)
- Not meeting inclusion criteria (n=)
- Declined to participate (n=)
- Other reasons (n=)
Completed baseline data (n=)
Randomised (n=)
Allocation
Allocated to intervention (n=)
- Received intervention (n=)
- Did not receive intervention (n=)
Allocated to waiting list control (n=)
6-month follow-up
Intervention
- Received intervention (n=)
- Did not receive intervention (n=)
Lost to follow-up (n=)
Lost to follow-up (n=)
12-month follow-up
Lost to follow-up (n=)
Lost to follow-up (n=)
Analysis
Analysed (n=)
Excluded from analysis (n=)
Analysed (n=)
Excluded from analysis (n=)

NHS
Guy's and St Thomas'
NHS Foundation Trust

KING'S College LONDON

IRAS ID:	279877
Centre Number:	
Participant Identification Number for this study:	
**CONSENT FORM FOR YOUNG PERSON ≥16 (PART A)**	
Title of Project:	A feasibility study and process evaluation of a psychosocial education programme for young people with type 1 diabetes - the Youth Empowerment Skills (YES)
Name of Researcher:	

Initial each box

1. I have read the *Participant Information Sheet* dated…………….. I **understand what the research project is about**, what will happen if I take part and I have had all my questions answered. ☐

2. I understand that I do not have to take part, and I know that I can stop taking part at any time without giving a reason. I know that **this will not make a difference to the care I receive from my diabetes team.** ☐

3. I give permission for researchers from King's College London to **look at my medical notes**. This will only be to find the information relevant to the above research project (as mentioned in the participant information sheet), and **only for the duration of the research,** which will end in August 2022**.** ☐

4. I agree to fill in **questionnaires** twice during the research project. ☐

5. I agree to take part in an **interview** (if asked) about the YES programme. The interview will be **audio-recorded** but will only be listened to by the researcher. Some of the things I say in the interview may be written about in research reports, but this will be **anonymous** (no one will be able to know who said it). ☐

6. I understand that people will be **chosen at random to have an interview**, and that I may not be offered one. ☐

7. I understand that information I give will be kept **confidential** (private) and stored in a safe place for up to 12 years after the project ends. ☐

8. I understand the **limits to confidentiality** as stated in the *Participant Information Sheet*. For example, if the researchers are worried about my safety or the safety of others they may tell someone else. ☐

9. I understand that if I leave the study early, the researchers will **still use the information** I have already given them. However, this information will **not have my** ☐

For peer review only - http://bmjopen.bmj.com/site/about/guidelines.xhtml

Guy's and St Thomas' NHS Foundation Trust

KING'S College LONDON

name on. Any information about me that includes personal details will be removed from the research project and archived (stored).

10. I understand that if I leave the study early, I will be asked if I am willing to take part in an **interview**, so the researchers can develop an understanding of what things might make a young person leave the study. I do not have to participate in this if I do not wish to. ☐

11. I understand that, for **auditing and monitoring purposes**, data collected during the research project may be looked at by people from King's College London, from regulatory authorities, or from the NHS Trust, where it is relevant to my taking part in the study. I give permission to these people to look my records for this reason. ☐

12. I understand that my **usual hospital diabetes team** will know that I am taking part in this research project. ☐

13. I understand that my **general practitioner (GP)** will know that I am taking part in this research project. ☐

14. **I agree to take part in the above research project.** ☐

Please sign and date the following section

___________________________	_______________	_________________________
Name of participant	Date	Signature
___________________________	_______________	_________________________
Name of person taking consent	Date	Signature

One copy for the participant, one copy for the researcher and one copy for the medical notes

For peer review only - http://bmjopen.bmj.com/site/about/guidelines.xhtml

Guy's and St Thomas' NHS Foundation Trust

KING'S College LONDON

IRAS ID:	279877
Centre Number:	
Participant Identification Number for this study:	
Title of Project:	A feasibility study and process evaluation of a psychosocial education programme for young people with type 1 diabetes - the Youth Empowerment Skills (YES)
Name of Researcher:	

ADDITIONAL CONSENT FORM (PART B)

Part B asks if you agree for us to keep your contact details after this research project is finished. This is so we can contact you about future research you might want to take part in. It is fine if you do not want us to keep your contact details for this purpose. You **do not** have to sign Part B to take part in this research project.

If you choose not to sign Part B, we will remove your name and contact details from our contacts list when this research project is finished, and we will not contact you about other research in the future. If you do sign Part B, you can still change your mind and ask us to remove your name and contact details from our contacts list **at any time**.

Initial each box

1. I agree that researchers at King's College London can keep my name and contact details on the project contacts list for_____years after this research project is finished. ☐
2. I understand that my name and contact details will be saved on a computer at King's College London and will be protected with a password. Only the researchers directly involved in this research project will have the password to access this information. ☐
3. I agree that these researchers from King's College London can use this information to contact me in the future. They will only contact me to let me know about other research that I can take part in **if I want to**. ☐
4. I understand that my name and contact details will **not** be used for any reason except that described above (number 3.). ☐
5. I understand that I can ask for my name and contact details to be removed from the ☐

For peer review only - http://bmjopen.bmj.com/site/about/guidelines.xhtml

Guy's and St Thomas' NHS Foundation Trust

KING'S College LONDON

contacts list at King's College London at any time. I know who to contact if I want to remove my details. I understand that researchers will not be able to contact me in the future if I remove my details.

6. I understand that if I do not want King's College London to keep my name and contact details on the contacts list after this project is finished, or if I ask for them to be removed at a later date:
   - This will **not** affect my involvement in the above research project.
   - This will **not** affect the care I receive from my diabetes team.

Please sign and date the following section

___________________________	______________	_________________________
Name of participant	Date	Signature
___________________________	______________	_________________________
Name of person taking consent	Date	Signature

One copy for the participant, one copy for the researcher and one copy for the medical notes

For peer review only - http://bmjopen.bmj.com/site/about/guidelines.xhtml
For peer review only

Guy's and St Thomas' NHS Foundation Trust

KING'S College LONDON

IRAS ID:	
Centre Number:	
Participant Identification Number for this study:	

ASSENT FORM FOR YOUNG PERSON <16 (PART A)

Title of Project:	A feasibility study and process evaluation of a psychosocial education programme for young people with type 1 diabetes - the Youth Empowerment Skills (YES)
Name of Researcher:	

Initial each box

1. I have read the *Participant Information Sheet* dated................ I **understand what the research project is about**, what will happen if I take part and I have had all my questions answered.

2. I understand that I do not have to take part, and I know that I can stop taking part at any time without giving a reason. I know that **this will not make a difference to the care I receive from my diabetes team.**

3. I give permission for researchers from King's College London to **look at my medical notes**. This will only be to find the information relevant to the above research project (as mentioned in the participant information sheet), and **only for the duration of the research,** which will end in August 2022**.**

4. I agree to fill in **questionnaires** twice during the research project.

5. I agree to take part in an **interview** (if asked) about the YES programme. The interview will be **audio-recorded** but will only be listened to by the researcher. Some of the things I say in the interview may be written about in research reports, but this will be **anonymous** (no one will be able to know who said it).

6. I understand that people will be **chosen at random to have an interview**, and that I may not be offered one.

7. I understand that information I give will be kept **confidential** (private) and stored in a safe place for up to twelve years after the project ends.

8. I understand the **limits to confidentiality** as stated in the *Participant Information Sheet*. For example, if the researchers are worried about my safety or the safety of others they may tell someone else.

9. I understand that if I leave the study early, the researchers will **still use the information** I have already given them. However, this information will **not have my name on**. Any information about me that includes personal details will be removed

For peer review only - http://bmjopen.bmj.com/site/about/guidelines.xhtml

Guy's and St Thomas' NHS Foundation Trust

KING'S College LONDON

from the research project and archived (stored).

10. I understand that if I leave the study early, I will be asked if I am willing to take part in an **interview**, so the researchers can develop an understanding of what things might make a young person leave the study. I do not have to participate in this if I do not wish to. ☐

11. I understand that, for **auditing and monitoring purposes**, data collected during the research project may be looked at by people from King's College London, from regulatory authorities, or from the NHS Trust, where it is relevant to my taking part in the study. I give permission to these people to look my records for this reason. ☐

12. I understand that my **usual diabetes team at ……………………… Hospital** will know that I am taking part in this research project. ☐

13. I understand that my **general practitioner (GP)** will know that I am taking part in this research project. ☐

14. **I agree to take part in the above research project.** ☐

Please sign and date the following section:

________________________	______________	______________________
Name of participant	Date	Signature
________________________	______________	______________________
Name of person taking assent	Date	Signature

One copy for the participant, one copy for the researcher and one copy for the medical notes.

For peer review only - http://bmjopen.bmj.com/site/about/guidelines.xhtml

IRAS ID:	
Centre Number:	
Participant Identification Number for this study:	
Title of Project:	A feasibility study and process evaluation of a psychosocial education programme for young people with type 1 diabetes - the Youth Empowerment Skills (YES)
Name of Researcher:	

ADDITIONAL ASSENT FORM (PART B)

Part B asks if you agree for us to keep your contact details after this research project is finished. This is so we can contact you about future research you might want to take part in. It is fine if you do not want us to keep your contact details for this purpose. You **do not** have to sign Part B to take part in this research project.

If you choose not to sign Part B, we will remove your name and contact details from our contacts list when this research project is finished, and we will not contact you about other research in the future. If you do sign Part B, you can still change your mind and ask us to remove your name and contact details from our contacts list **at any time**.

Initial each box

1. I agree that researchers at King's College London can keep my name and contact details on the project contacts list for_____years after this research project is finished. ☐

2. I understand that my name and contact details will be saved on a computer at King's College London and will be protected with a password. Only the researchers directly involved in this research project will have the password to access this information. ☐

3. I agree that these researchers from King's College London can use this information to contact me in the future. I understand that they will always contact my parents first. They will only contact me to let me know about other research that I can take part in **if I want to**. ☐

4. I understand that my name and contact details will **not** be used for any reason except ☐

For peer review only - http://bmjopen.bmj.com/site/about/guidelines.xhtml

that described above (number 3).

5. I understand that I can ask for my name and contact details to be removed from the contacts list at King's College London at any time. I know who to contact if I want to remove my details. I understand that researchers will not be able to contact me in the future if I remove my details.

6. I understand that if I do not want King's College London to keep my name and contact details on the contacts list after this project is finished, or if I ask for them to be removed at a later date:
   - This will **not** affect my involvement in the above research project.
   - This will **not** affect the care I receive from my diabetes team.

Please sign and date the following section:

___________________________	_______________	_________________________
Name of participant	Date	Signature
___________________________	_______________	_________________________
Name of person taking assent	Date	Signature

One copy for the participant, one copy for the researcher and one copy for the medical notes.

For peer review only - http://bmjopen.bmj.com/site/about/guidelines.xhtml

SPIRIT
STANDARD PROTOCOL ITEMS: RECOMMENDATIONS FOR INTERVENTIONAL TRIALS

SPIRIT 2013 Checklist: Recommended items to address in a clinical trial protocol and related documents*

Section/item	Item No	Description	Page No in the MS
**Administrative information**			
Title	1	Descriptive title identifying the study design, population, interventions, and, if applicable, trial acronym	1
Trial registration	2a	Trial identifier and registry name. If not yet registered, name of intended registry	2
	2b	All items from the World Health Organization Trial Registration Data Set	1-19
Protocol version	3	Date and version identifier	2
Funding	4	Sources and types of financial, material, and other support	18
Roles and responsibilities	5a	Names, affiliations, and roles of protocol contributors	1, 19
	5b	Name and contact information for the trial sponsor	18
	5c	Role of study sponsor and funders, if any, in study design; collection, management, analysis, and interpretation of data; writing of the report; and the decision to submit the report for publication, including whether they will have ultimate authority over any of these activities	18
	5d	Composition, roles, and responsibilities of the coordinating centre, steering committee, endpoint adjudication committee, data management team, and other individuals or groups overseeing the trial, if applicable (see Item 21a for data monitoring committee)	n/a

For peer review only

**Introduction**			
Background and rationale	6a	Description of research question and justification for undertaking the trial, including summary of relevant studies (published and unpublished) examining benefits and harms for each intervention	**4-8**
	6b	Explanation for choice of comparators	**10**
Objectives	7	Specific objectives or hypotheses	**7-8**
Trial design	8	Description of trial design including type of trial (eg, parallel group, crossover, factorial, single group), allocation ratio, and framework (eg, superiority, equivalence, noninferiority, exploratory)	**8-9**
**Methods: Participants, interventions, and outcomes**			
Study setting	9	Description of study settings (eg, community clinic, academic hospital) and list of countries where data will be collected. Reference to where list of study sites can be obtained	**9**
Eligibility criteria	10	Inclusion and exclusion criteria for participants. If applicable, eligibility criteria for study centres and individuals who will perform the interventions (eg, surgeons, psychotherapists)	**9**
Interventions	11a	Interventions for each group with sufficient detail to allow replication, including how and when they will be administered	**9, 10-11**
	11b	Criteria for discontinuing or modifying allocated interventions for a given trial participant (eg, drug dose change in response to harms, participant request, or improving/worsening disease)	**n/a**
	11c	Strategies to improve adherence to intervention protocols, and any procedures for monitoring adherence (eg, drug tablet return, laboratory tests)	**n/a**
	11d	Relevant concomitant care and interventions that are permitted or prohibited during the trial	**n/a**

For peer review only - http://bmjopen.bmj.com/site/about/guidelines.xhtml

Outcomes	12	Primary, secondary, and other outcomes, including the specific measurement variable (eg, systolic blood pressure), analysis metric (eg, change from baseline, final value, time to event), method of aggregation (eg, median, proportion), and time point for each outcome. Explanation of the clinical relevance of chosen efficacy and harm outcomes is strongly recommended	**10-11**
Participant timeline	13	Time schedule of enrolment, interventions (including any run-ins and washouts), assessments, and visits for participants. A schematic diagram is highly recommended (see Figure)	**Figure 1**
Sample size	14	Estimated number of participants needed to achieve study objectives and how it was determined, including clinical and statistical assumptions supporting any sample size calculations	**9-10**
Recruitment	15	Strategies for achieving adequate participant enrolment to reach target sample size	**11**
**Methods: Assignment of interventions (for controlled trials)**			
Allocation:			
Sequence generation	16a	Method of generating the allocation sequence (eg, computer-generated random numbers), and list of any factors for stratification. To reduce predictability of a random sequence, details of any planned restriction (eg, blocking) should be provided in a separate document that is unavailable to those who enrol participants or assign interventions	**10**
Allocation concealment mechanism	16b	Mechanism of implementing the allocation sequence (eg, central telephone; sequentially numbered, opaque, sealed envelopes), describing any steps to conceal the sequence until interventions are assigned	**10**
Implementation	16c	Who will generate the allocation sequence, who will enrol participants, and who will assign participants to interventions	**10**
Blinding (masking)	17a	Who will be blinded after assignment to interventions (eg, trial participants, care providers, outcome assessors, data analysts), and how	**10**

For peer review only - http://bmjopen.bmj.com/site/about/guidelines.xhtml

	17b	If blinded, circumstances under which unblinding is permissible, and procedure for revealing a participant's allocated intervention during the trial	**10**
**Methods: Data collection, management, and analysis**			
Data collection methods	18a	Plans for assessment and collection of outcome, baseline, and other trial data, including any related processes to promote data quality (eg, duplicate measurements, training of assessors) and a description of study instruments (eg, questionnaires, laboratory tests) along with their reliability and validity, if known. Reference to where data collection forms can be found, if not in the protocol	**10-12**
	18b	Plans to promote participant retention and complete follow-up, including list of any outcome data to be collected for participants who discontinue or deviate from intervention protocols	**11-14**
Data management	19	Plans for data entry, coding, security, and storage, including any related processes to promote data quality (eg, double data entry; range checks for data values). Reference to where details of data management procedures can be found, if not in the protocol	**12**
Statistical methods	20a	Statistical methods for analysing primary and secondary outcomes. Reference to where other details of the statistical analysis plan can be found, if not in the protocol	**12-14**
	20b	Methods for any additional analyses (eg, subgroup and adjusted analyses)	**12-14**
	20c	Definition of analysis population relating to protocol non-adherence (eg, as randomised analysis), and any statistical methods to handle missing data (eg, multiple imputation)	**12**
**Methods: Monitoring**			
Data monitoring	21a	Composition of data monitoring committee (DMC); summary of its role and reporting structure; statement of whether it is independent from the sponsor and competing interests; and reference to where further details about its charter can be found, if not in the protocol. Alternatively, an explanation of why a DMC is not needed	**16**

	21b	Description of any interim analyses and stopping guidelines, including who will have access to these interim results and make the final decision to terminate the trial	**16**
Harms	22	Plans for collecting, assessing, reporting, and managing solicited and spontaneously reported adverse events and other unintended effects of trial interventions or trial conduct	**16**
Auditing	23	Frequency and procedures for auditing trial conduct, if any, and whether the process will be independent from investigators and the sponsor	**n/a**
**Ethics and dissemination**			
Research ethics approval	24	Plans for seeking research ethics committee/institutional review board (REC/IRB) approval	**18**
Protocol amendments	25	Plans for communicating important protocol modifications (eg, changes to eligibility criteria, outcomes, analyses) to relevant parties (eg, investigators, REC/IRBs, trial participants, trial registries, journals, regulators)	**16**
Consent or assent	26a	Who will obtain informed consent or assent from potential trial participants or authorised surrogates, and how (see Item 32)	**18**
	26b	Additional consent provisions for collection and use of participant data and biological specimens in ancillary studies, if applicable	**n/a**
Confidentiality	27	How personal information about potential and enrolled participants will be collected, shared, and maintained in order to protect confidentiality before, during, and after the trial	**18**
Declaration of interests	28	Financial and other competing interests for principal investigators for the overall trial and each study site	**19**
Access to data	29	Statement of who will have access to the final trial dataset, and disclosure of contractual agreements that limit such access for investigators	**16**

For peer review only

For peer review only - http://bmjopen.bmj.com/site/about/guidelines.xhtml

Ancillary and post-trial care	30	Provisions, if any, for ancillary and post-trial care, and for compensation to those who suffer harm from trial participation	**16**
Dissemination policy	31a	Plans for investigators and sponsor to communicate trial results to participants, healthcare professionals, the public, and other relevant groups (eg, via publication, reporting in results databases, or other data sharing arrangements), including any publication restrictions	**18**
	31b	Authorship eligibility guidelines and any intended use of professional writers	**19**
	31c	Plans, if any, for granting public access to the full protocol, participant-level dataset, and statistical code	**16**
**Appendices**			
Informed consent materials	32	Model consent form and other related documentation given to participants and authorised surrogates	**18**
Biological specimens	33	Plans for collection, laboratory evaluation, and storage of biological specimens for genetic or molecular analysis in the current trial and for future use in ancillary studies, if applicable	**n/a**

*It is strongly recommended that this checklist be read in conjunction with the SPIRIT 2013 Explanation & Elaboration for important clarification on the items. Amendments to the protocol should be tracked and dated. The SPIRIT checklist is copyrighted by the SPIRIT Group under the Creative Commons "Attribution-NonCommercial-NoDerivs 3.0 Unported" license.

For peer review only
