# Supplementary material for: Protocol for a feasibility study and process evaluation of a psychosocially modelled diabetes education programme for young people with type 1 diabetes: the Youth Empowerment Skills (YES) programme
Source: BMJ Open. 2022 Jun 8;12(6):e062971. doi: 10.1136/bmjopen-2022-062971 (PMC9185576; doi:10.1136/bmjopen-2022-062971)
Supplement: Reviewer comments [file bmjopen-2022-062971.reviewer_comments.pdf]

PEER REVIEW HISTORY

BMJ Open publishes all reviews undertaken for accepted manuscripts. Reviewers are asked to complete a checklist review form (**http://bmjopen.bmj.com/site/about/resources/checklist.pdf**) and are provided with free text boxes to elaborate on their assessment. These free text comments are reproduced below.

ARTICLE DETAILS

**TITLE (PROVISIONAL)**	Protocol for A Feasibility Study and Process Evaluation of a Psychosocially Modelled Diabetes Education Programme for Young People with Type 1 Diabetes: the Youth Empowerment Skills (YES) Programme
**AUTHORS**	Kariyawasam, Dulmini; Soukup, Tayana; Parsons, Judith; Sevdalis, Nick; Baldellou Lopez, Maria; Forde, Rita; Ismail, Khalida; Jones, Marie; Ford-Adams, Martha; Yemane, Nardos; Pender, Siobhan; Thomas, Stephen; Murrells, Trevor; Silverstien, Alex; Forbes, Angus

This article was not externally reviewed at BMJ Open. Protocols that have been independently assessed prior to submission to BMJ Open are usually fast-tracked to publication on the grounds that further substantial changes will not be possible. This independent assessment will usually be external, independent review for both a substantial grant award from a non-commercial or government funder and ethics approval.
